# Comparative effectiveness of ten traditional Chinese herbal formulas for acute exacerbation of chronic obstructive pulmonary disease: a systematic review and Bayesian network meta-analysis

**DOI:** 10.3389/fphar.2025.1585150

**Published:** 2026-01-15

**Authors:** Enze Yu, Chunyan Tian, Pengna Jiang, Xingyi Wang, Yuting Yin, Zhuying Li

**Affiliations:** 1 First School of Clinical Medicine, Heilongjiang University of Chinese Medicine, Harbin, China; 2 Respiratory Department, The First Affiliated Hospital of Heilongjiang University of Traditional Chinese Medicine, Harbin, China

**Keywords:** acute exacerbation of chronic obstructive pulmonary disease, certainty of evidence, network meta-analysis, randomized controlled trials, systematic review, traditional Chinese herbal formulas

## Abstract

**Background:**

Traditional Chinese medicine (TCM) has emerged as a valuable adjunctive approach for respiratory diseases, with accumulating evidence supporting its efficacy in Acute Exacerbation of Chronic Obstructive Pulmonary Disease (AECOPD) management. However, most existing randomized controlled trials (RCTs) and meta-analyses have focused on evaluating single traditional Chinese herbal formulas as an add-on to conventional therapy (CT). The comparative efficacy among these formulas remains uncertain, creating a critical evidence gap that hampers clinical decision-making. Network meta-analysis (NMA) provides a methodological framework to address this limitation by enabling simultaneous comparison of multiple interventions.

**Methods:**

RCTs of these ten herbal formulas in combination with CT for the treatment of AECOPD were searched in five English and four Chinese databases. The search covered the period from the date of database inception to 20 August 2024, with a final update performed on 28 November 2025. The risk of bias of the included RCTs was critically assessed using the Cochrane Risk-of-Bias tool for randomized trials, version 2 (RoB 2). Bayesian NMA, pairwise meta-analysis (PMA), meta-regression, subgroup and sensitivity analyses, and publication bias analysis were carried out. The certainty of evidence was evaluated using the Grading of Recommendations Assessment, Development and Evaluation (GRADE) approach.

**Results:**

A total of 132 eligible RCTs involving 13,177 participants were included. Both PMA and NMA demonstrated that all ten herbal formulas combined with CT were more effective than CT alone in treating AECOPD. Based on the ranking results, Qingqihuatan decoction (QQHT), Erchen decoction combined with Sanziyangqin decoction (ECSZYQ), and Weijing decoction (WJ) had the highest probability of being the most efficacious interventions in improving lung function (FEV_1_, FEV_1_%pred, FEV_1_/FVC). For the correction of arterial blood gas abnormalities (PaO_2_, PaCO_2_), Maxingshigan decoction (MXSG), Xiaoqinglong decoction (XQL), and Yuebi Jia Banxia decoction (YBBX) were ranked highest. However, the GRADE framework rated the certainty of evidence for NMA and most PMA results as “very low”.

**Conclusion:**

Based on the current evidence, QQHT and XQL appear to be the most promising herbal formulas. Nevertheless, given the low certainty of evidence, caution is warranted in interpreting these findings. The final selection in clinical practice should be guided by a comprehensive assessment of the patient’s specific pathophysiological profile and individual factors. Further high-quality, head-to-head comparative studies are needed to validate these findings.

**Systematic Review Registration:**

https://www.crd.york.ac.uk/PROSPERO/, identifier CRD42024622734.

## 1 Introduction

Chronic Obstructive Pulmonary Disease (COPD) is a condition with high morbidity and mortality worldwide (Christenson et al., 2022). COPD mortality increased globally by 35.4% between 2009 and 2019, with projections indicating that it will become the world’s third leading cause of death by 2030 (Al Wachami et al., 2024). Acute exacerbation of COPD, characterized by a sudden worsening of respiratory symptoms, leads to progressive lung function decline, reduced quality of life, increased hospitalization rates, and higher mortality (Hatipoglu and Aboussouan, 2016). Despite the effectiveness of pharmacologic regimens recommended by clinical guidelines for COPD management, acute exacerbations remain frequent in patients (Ko et al., 2016). Moreover, long-term use of corticosteroids and bronchodilators as conventional therapy is associated with adverse events, including osteoporosis, hypertension, and increased infection risk (Cave and Hurst, 2011; Bourbeau, 2024). There is a pressing need to identify safer and more effective strategies for the management of AECOPD.

The World Health Organization (WHO) recognizes herbal medicine as an important component in promoting human health (von Schoen-Angerer et al., 2023). In China, the use of herbal formulas for the treatment of respiratory diseases dates back thousands of years and has a deep theoretical and practical foundation. Chinese clinical guidelines for COPD recommend various therapeutic strategies (Li et al., 2012; Li et al., 2023; Wu et al., 2025), which include ten herbal formulas: Dingchuan decoction (DC), Maxingshigan decoction (MXSG), Xiaoqinglong decoction (XQL), Yuebi Jia Banxia decoction (YBBX), Qingqihuatan decoction (QQHT), Weijing decoction (WJ), Xuanbaichengqi decoction (XBCQ), Sangbaipi decoction (SBP), Suzijiangqi decoction (SZJQ), and Erchen decoction combined with Sanziyangqin decoction (ECSZYQ).

Guided by the theory of syndrome differentiation in Traditional Chinese Medicine (TCM), these ten formulas are often used to treat COPD patients presenting with “Excess Syndrome”. Their clinical manifestations, such as a significant increase in sputum volume and exacerbation of respiratory distress within a short period, are closely aligned with the pathological manifestations of AECOPD. These formulas are frequently combined with conventional therapy to treat AECOPD in clinical practice (Wen et al., 2023). Accumulating evidence from clinical trials and meta-analyses suggests that these herbal interventions may play a crucial role in enhancing clinical outcomes, reducing the frequency of exacerbations, and alleviating the overall disease burden (Gao et al., 2020; He et al., 2025; Lu et al., 2025).

Modern pharmacological studies have further confirmed the therapeutic properties of these herbal formulas and their active ingredients, including anti-inflammatory, antioxidant, immunomodulatory, anti-apoptotic, and anti-airway remodeling properties (Cao et al., 2023). Furthermore, metabolomics studies have begun to elucidate the specific pathways involved. For instance, the anti-inflammatory effects of heat-clearing and phlegm-resolving herbal formulas were associated with decreased levels of lysophosphatidylcholines (LPCs). This reduction in LPCs showed significant positive correlations with reductions in C-reactive protein (CRP), white blood cell count (WBC), and neutrophil percentage (NEU%). Similarly, symptom improvement from dampness-drying and phlegm-resolving herbal formulas was linked to alterations in carnitine-related metabolites, which showed positive correlations with both the CAT score and mMRC dyspnea scale (Hailong et al., 2024). These metabolite-level shifts offer objective biomarkers for evaluating the therapeutic efficacy within the TCM diagnosis and treatment system.

Due to the scarcity of direct comparisons between these herbal formulas, it is difficult to determine which ones are more therapeutically advantageous in the treatment of AECOPD. Network meta-analysis (NMA), which is not limited by head-to-head comparisons, is increasingly used to recommend optimal treatment regimens in medical decision-making (Kiefer et al., 2015; Li et al., 2022). Therefore, this analysis employs NMA to explore the efficacy ranking of these formulas.

Previous TCM research has largely overlooked the differences in dosage forms (including but not limited to decoctions, pills, granules, tablets, and capsules). Variations in dosage forms can affect the efficacy of herbal formulas through multiple factors, such as the processing of herbal materials, dosage, and the bioavailability (Duan et al., 2019). Ignoring these differences may significantly compromise the reliability of research conclusions. Despite the emergence of some modern dosage forms, traditional decoctions remain widely used in clinical practice due to their notable therapeutic effects and flexible customization (Luo et al., 2021). By focusing on a single dosage form (traditional decoction), this study not only addresses a gap in existing research but also provides evidence-based support for shared decision-making among patients, caregivers, and clinicians.

In fact, when making clinical decisions, physicians need to weigh the certainty of the efficacy estimates above and beyond the benefits and risks of a treatment regimen (Barry et al., 2023; Jia et al., 2023). The Grading of Recommendations Assessment, Development and Evaluation (GRADE) methodology, developed to assess the certainty of evidence, has been extended to the field of meta-analysis (Neumann et al., 2016; Brignardello-Petersen et al., 2018). Consequently, while ensuring sufficient sample sizes and rigorous statistical methods, it is imperative that NMAs are fully reported in accordance with the PRISMA-NMA statement (Hutton et al., 2015; Page et al., 2021). These analyses may serve to provide the best and most up-to-date evidence for comparing the therapeutic efficacy of herbal formulas used to treat AECOPD.

## 2 Methods

### 2.1 Systematic review registration

This meta-analysis adhered to the Preferred Reporting Items for Systematic Reviews and Meta-Analyses (*PRISMA*) 2020 statement and the *PRISMA* extension statement for network meta-analyses (*PRISMA-NMA*) statement. The study protocol was registered in the International Prospective Register of Systematic Reviews (PROSPERO) database (registration number: CRD42024622734). Details are provided in Supplementary Tables A.1, A.2.

### 2.2 Standard evaluation of herbal formulas

To ensure methodological rigor, the study adhered to the Consensus statement on the Phytochemical Characterisation of Medicinal Plant extracts (ConPhyMP) (Heinrich et al., 2020; Heinrich et al., 2022). The botanical nomenclature for all medicinal plant components was standardized using the Medicinal Plant Names Service (MPNS, http://mpns.kew.org/mpns-portal/) and/or Plants of the World Online (POWO, http://www.plantsoftheworldonline.org). Detailed information on composition, dosage, processing methods, decoction preparation techniques for classical herbal formulas, along with taxonomically validated nomenclature, is documented in Supplementary Tables A.3–A.5.

### 2.3 Literature search

We systematically searched PubMed, EMBASE, Cochrane Central Register of Controlled Trials (CENTRAL), Cumulative Index to Nursing and Allied Health Literature (CINAHL), Web of Science, Chinese Biomedical Database (CBM), China National Knowledge Infrastructure (CNKI), Chongqing VIP Information Chinese Science and Technology Journal Database (VIP Database), and Wanfang Data Knowledge Service Platform (Wanfang Database) from the date of database creation to 20 August 2024, and performed a final update on 28 November 2025. Additionally, the reference lists of retrieved relevant systematic reviews/meta-analyses were screened to ensure the comprehensiveness of the literature search. The search terms are available at Supplementary Tables A.6–A.14.

### 2.4 Inclusion and exclusion criteria

#### 2.4.1 Inclusion criteria

1. Participants: Patients aged ≥ 40 years diagnosed with AECOPD according to the Global Initiative for Chronic Obstructive Lung Disease (GOLD) criteria or the Chinese expert consensus on the diagnosis and treatment of AECOPD, with no restrictions regarding gender or geographic region.
2. Intervention: One of the ten Chinese herbal formulas combined with conventional therapy (CT). The formulas could be modified with appropriate additions or deletions of herbs, provided that the core composition of the standard formula is maintained. The mode of administration is limited to the oral route, with the drug dosage form restricted to aqueous decoctions (traditional decoctions, not ready-to-use granules).
3. Control: CT alone, CT combined with another one of the ten herbal formulas, or CT combined with placebo. In the same randomized controlled trial, the CT regimen must remain consistent across all treatment groups.
4. Outcome measures:

- Primary outcomes:

Pulmonary function indicators and arterial blood gas parameters were selected as the primary outcomes because they are recognized by guidelines as objective measurement tools for clinical assessment that reflect core pathophysiological processes, thereby minimizing subjectivity and bias in the assessment of endpoints.

- Pulmonary function indicators: Forced Expiratory Volume in 1 s (FEV_1_), FEV_1_/predicted (FEV_1_%pred), FEV_1_/Forced Vital Capacity (FEV_1_/FVC).
- Arterial blood gas parameters: Partial Pressure of Arterial Oxygen (PaO_2_), Partial Pressure of Arterial Carbon Dioxide (PaCO_2_).

ii. Secondary outcomes:

- Effective rate. The effective rate was defined as the proportion of participants in each trial arm who achieved any level of positive response (excluding “ineffective” outcomes), encompassing those categorized as “clinically controlled,” “markedly effective,” or “effective.”
- Quality of life (QoL) evaluation. QoL was assessed using the following validated instruments: St. George’s Respiratory Questionnaire (SGRQ), Chronic Obstructive Pulmonary Disease Assessment Test (CAT), and Clinical COPD Questionnaire (CCQ).
- Exacerbation recurrence.
- Safety profile.

Studies were eligible for inclusion if they reported at least one pulmonary function indicator.

5. Study design: Randomized controlled trials (RCTs) published in English or Chinese.

#### 2.4.2 Exclusion criteria

1. Studies involving participants with other serious diseases, such as heart failure or malignant tumors.
2. Dissertations or thesis studies.
3. Non-RCT.
4. Studies with insufficient sample size (fewer than 35 participants in each group) to meet the fundamental requirements for statistical power and normality assumptions.
5. Duplicate publications.
6. Studies with unavailable data or serious errors resulting in implausible data.

### 2.5 Study selection and data extraction

Two researchers (EY and CT) independently conducted the initial screening of the literature using EndNote 20. Titles and abstracts were reviewed to exclude studies that did not meet the inclusion criteria. Subsequently, the full texts of potentially eligible studies were assessed to determine their suitability for inclusion in the quantitative analysis. In cases of disagreement, a third researcher (PJ) was consulted to reach a consensus.

The extracted data included: 1. Basic information of the included studies: title, first author, and year of publication; 2. Baseline characteristics of the study population: sample size, sex, age, diagnosis information, disease severity, disease duration, intervention details, treatment duration, and follow-up period; 3. Prespecified outcome measures; 4. Risk of bias assessment: randomization sequence generation, allocation concealment, and blinding methods.

### 2.6 Risk of bias assessment and certainty of evidence

The methodological quality of the included RCTs was independently assessed by two reviewers (EY and CT) using the Cochrane Risk-of-Bias tool for randomized trials, version 2 (RoB 2) (Sterne et al., 2019). The tool evaluates five aspects: randomization process, deviations from intended interventions, missing outcome data, measurement of the outcome, and selection of reported result. Each aspect was assessed and categorized as “low risk,” “some concerns,” or “high risk.” The overall risk of bias for each RCT was determined based on the assessments across the five domains. Discrepancies were resolved through discussion with a third investigator (PJ).

The certainty of evidence for this meta-analysis was assessed using the GRADE method. The quality of evidence was downgraded based on the five GRADE criteria for limitations: risk of bias, inconsistency, indirectness, imprecision, and publication bias. The outcomes were categorized into four evidence levels: very low, low, moderate, and high quality.

### 2.7 Data synthesis and analysis

A random-effects model was used for all analyses given the anticipated heterogeneity (Makinde et al., 2021). This approach provides a more conservative effect estimate when heterogeneity is present. Continuous variables (such as FEV_1_) and dichotomous variables (such as effective rate) were analyzed as mean difference (MD) and odds ratio (OR), respectively. When outcomes were measured using different scales (such as different quality of life questionnaires), the pooled effect was estimated using the standardized mean difference (SMD), calculated as Hedges' g to adjust for small sample bias. Heterogeneity was quantified using the *I* ^*2*^ statistic, with *I* ^*2*^ > 50% indicating substantial heterogeneity.

For NMA, the Markov chain Monte Carlo method was implemented to compare the therapeutic effects among ten herbal formulas. The posterior distributions were calculated by using 100,000 iterations, where the first burn-in was 20,000 iterations. Based on the Brooks–Gelman–Rubin diagnostic, model convergence was considered achieved when the potential scale reduction factor (PSRF) approached a value of 1.00. Otherwise, iterations were continued until this criterion was met. Adjusted indirect comparisons were estimated for dichotomous and continuous variables using 95% credible intervals (CrIs). The relevant analysis code and raw data are provided in the Supplementary Tables A.34–A.36.

The transitivity assumption was evaluated by assessing the distribution of clinical (such as baseline age, sex distribution, disease severity, and TCM syndrome diagnosis) and methodological variables that could act as effect modifiers across treatment comparisons (Jansen and Naci, 2013; Cipriani et al., 2018). As pre-specified, had any closed loops been present in the evidence network, Bayesian node-splitting methods were utilized to separate direct and indirect evidence for specific comparisons (Dias et al., 2010). The inconsistency factor (IF) was calculated along with its 95% CrIs. Statistical significance of inconsistency was determined based on whether the 95% CrI included zero. In cases of significant inconsistency, inconsistency models were preferentially employed for analysis.

To explore the best interventions, rankings were generated using the Surface Under the Cumulative Ranking Curve (SUCRA). A higher SUCRA value indicates better treatment efficacy for the corresponding intervention. The resulting ranking matrices were further explored using two-dimensional cluster analysis to group interventions with similar efficacy profiles across multiple outcomes.

Additionally, meta-regression analysis was performed based on the following factors: overall risk of bias, sample size (dichotomized by the mean sample size of all included studies), the composition of CT regimens, treatment duration (≤7, 8–14, >14 days), adherence to standard processing of core herbs (yes/no), herbal formula modification (unmodified, 1–3, >3 herbs), disease duration (≤7 vs. >7 years). Subgroup analyses were conducted for comparisons involving more than ten RCTs.

The robustness of the findings was tested through a series of sensitivity analyses that separately applied the following criteria: i. excluding RCTs judged to be at high overall risk of bias, ii. including only RCTs employing a consistent CT regimen, iii. restricting to RCTs using pharmacognostically defined herbal formulas, defined as those with unmodified classical compositions and standard processing of core herbs, and iv. sequential exclusion of each high-risk RCT (leave-one-out analysis).

Publication bias was assessed using Egger’s regression test, complemented by comparison-adjusted funnel plots. A significance threshold of *P* < 0.05 was set for statistical tests.

All these analyses, including PMA and NMA, were performed using WinBUGS 1.4.3 (MRC Biostatistics Unit, Cambridge, United Kingdom) and R 4.2.1 (R Foundation for Statistical Computing, Vienna, Austria). Graphical presentations were generated using Stata/IC 15.0 (StataCorp LLC, College Station, TX, United States).

## 3 Results

### 3.1 Study selection

Following the application of predefined inclusion and exclusion criteria, a total of 3,974 citations were initially retrieved through the database search.

After removing duplicates, 2,023 records were screened by titles and abstracts. After excluding 1,758 records due to reasons such as ineligible study populations, the remaining 265 articles underwent a detailed full-text assessment.

Subsequently, 137 articles were excluded for the following reasons: non-RCT design (36 studies), insufficient sample size per predefined criteria (68 studies), non-conformity or unclear description of interventions (10 studies), outcome measures not meeting inclusion criteria (4 studies), unavailable or critically flawed data (18 studies), and duplicate publication (1 study).

Using the same search strategy and inclusion/exclusion criteria, a repeat search was conducted on 28 November 2025, to incorporate newly published studies (4 studies).

**FIGURE 1:**
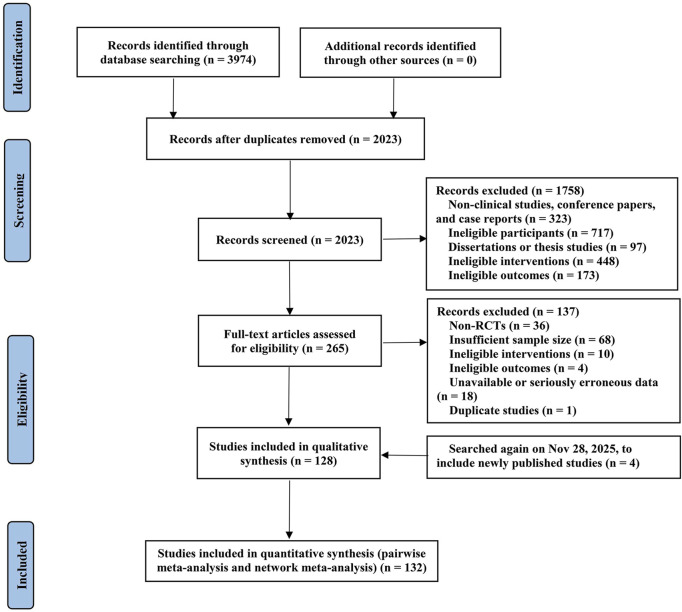
Summary of trial identification and selection.

Ultimately, 132 RCTs were considered eligible and included in the meta-analysis. The detailed literature screening process is illustrated in Figure 1.

### 3.2 Characteristics of included studies

These 132 RCTs involved 13,177 participants, with sample sizes per study ranging from 70 to 316 (mean = 99.83). All RCTs compared the combination of an herbal formula with CT against CT alone. All RCTs were conducted in China and published between 2009 and 2025. The basic characteristics of the included studies are summarized in Supplementary Table A.15.

Among the included trials, XQL was the most frequently investigated formula (28, 21.21%), followed by SZJQ (23, 17.42%), MXSG (21, 15.91%), ECSZYQ (15, 11.36%), WJ (10, 7.58%), SBP (10, 7.58%), DC (8, 6.06%), QQHT (8, 6.06%), XBCQ (5, 3.79%), and YBBX (4, 3.03%).

Syndrome differentiation, a core principle of TCM, was incorporated into the inclusion criteria of 71 RCTs (53.79%). In these trials, the TCM syndrome patterns were theoretically consistent with the applied herbal formulas, adhering to the principle of syndrome differentiation and treatment determination. In clinical practice, practitioners frequently modify classical herbal formulas based on patients’ specific syndrome patterns and clinical manifestations. Detailed information on the application of these ten formulas in the included studies is provided in Supplementary Tables A.16, A.17.

The reported pulmonary function indices included FEV_1_ (89 RCTs, 67.42%), FEV_1_%pred (57 RCTs, 43.18%), and FEV_1_/FVC (101 RCTs, 76.52%). Arterial blood gas parameters PaO_2_ and PaCO_2_, were reported in 38 of these trials (28.79%). The effective rate was reported in 116 RCTs (87.88%), making it the most frequently reported outcome measure. Quality of life was assessed in 10 RCTs (7.58%), and safety profile was reported in 39 RCTs (29.55%). Only 5 RCTs (3.79%) included a follow-up period, with 3 of them investigating the exacerbation recurrence events.

### 3.3 Quality assessment

**FIGURE 2:**
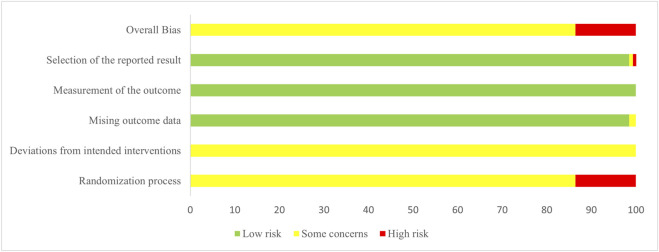
Risk of bias graph.

The methodological quality of the included RCTs was substantially limited by poor reporting practices. Critically, most trials failed to describe allocation concealment, and the implementation of blinding was universally unreported, thereby undermining the robustness of the results. The complete risk of bias assessment is detailed in Figure 2 and Supplementary Figure A.1.

### 3.4 Pairwise meta-analysis

#### 3.4.1 Primary outcomes

PMAs demonstrated that the adjunct use of herbal formulas significantly improved lung function and arterial blood gas parameters in AECOPD patients. Of the 55 analyses performed, 36 showed statistically significant treatment effects (Supplementary Table A.18).

For lung function, all ten combinations significantly improved FEV_1_/FVC (MDs: 4.58 to 10.40). Regarding both FEV_1_ (MDs: 0.31 to 0.56) and FEV_1_%pred (MDs: 3.46 to 8.77), nine combinations conferred significant benefits, with YBBX + CT being the only exception (MD = 0.25, 95% CrI: −0.04 to 0.53; MD = 6.79, 95% CrI: −1.66 to 15.23).

Analysis of eight herbal formulas revealed distinct effects on arterial blood gas parameters. Six combinations significantly improved PaO_2_ (MDs: 5.31 to 12.55) and reduced PaCO_2_ (MDs: −5.17 to −10.24). However, two combinations showed no significant benefit with respect to PaO_2_ and PaCO_2_, respectively. DC + CT and SBP + CT failed to elevate PaO_2_, while SBP + CT and YBBX + CT did not significantly reduce PaCO_2_.

#### 3.4.2 Secondary outcomes

The effective rate was significantly higher with all ten combinations compared with CT alone, with odds ratios (ORs) ranging from 3.53 to 6.50. No significant heterogeneity was observed across these comparisons.

Quality of life (QoL) was assessed in ten RCTs (910 participants) using validated multidimensional instruments. The pooled analysis demonstrated that all five evaluated combination therapies (XQL + CT, DC + CT, SZJQ + CT, MXSG + CT, and ECSZYQ + CT) yielded significantly greater improvements compared with CT alone (SMD = −1.71, 95% CrI: −2.31 to −1.12, *I* ^*2*^ = 93.1%).

Recurrence outcomes were reported in three RCTs. DC + CT significantly reduced the recurrence rate within 6 months post-treatment compared with CT alone, based on one RCT. Similarly, two additional RCTs (on SZJQ + CT and ECSZYQ + CT respectively) reported significant reductions in the number of acute exacerbations.

### 3.5 The results of NMA

The assessment of potential effect modifier distributions revealed no significant imbalances, thereby supporting the validity of the transitivity assumption. Furthermore, the network structure contained no closed loops, precluding statistical evaluation for consistency. Based on these findings and in accordance with the pre-specified analysis plan, the consistency assumption was considered justified, and a consistency model was consequently applied throughout the analysis.

#### 3.5.1 FEV_1_

**FIGURE 3:**
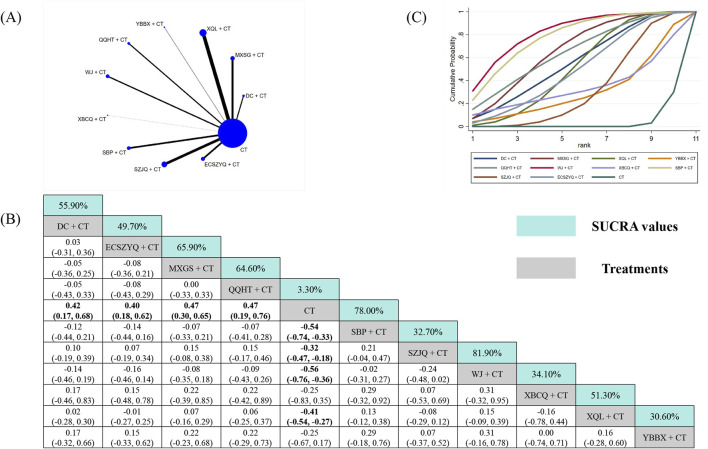
Network meta-analysis of FEV_1_. **(A)** Network evidence plot. **(B)** Summary of relative effect sizes. Numbers in the light blue boxes are the values of SUCRA. Results with statistically significant differences are highlighted in bold. **(C)** Cumulative ranking probability plot.

Data from 89 RCTs were included in the FEV_1_ analysis (Figure 3A). Compared with CT alone, eight of the ten combinations resulted in statistically significant improvements in FEV_1_. The exceptions were XBCQ + CT (MD = 0.25, 95% CrI: −0.35 to 0.83) and YBBX + CT (MD = 0.25, 95% CrI: −0.17 to 0.67). SUCRA analysis identified WJ + CT (81.90%) as the most probable optimal treatment, followed by SBP + CT (78.00%) and MXSG + CT (65.90%) (Figure 3C). However, estimates from the NMA indicated that WJ + CT did not demonstrate superiority over any other combinations (Figure 3B).

#### 3.5.2 FEV_1_%pred

**FIGURE 4:**
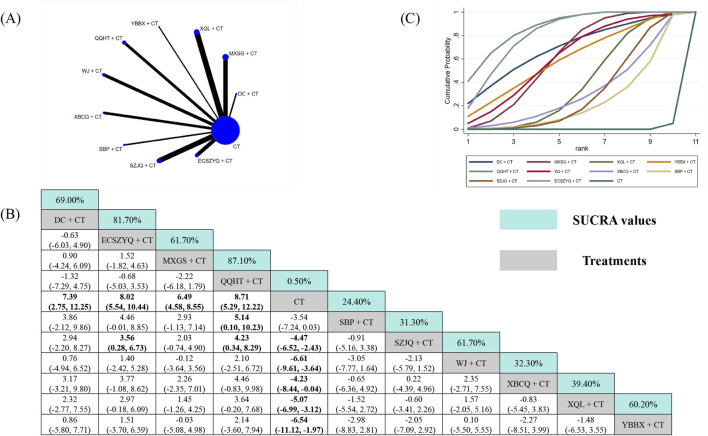
Network meta-analysis of FEV_1_%pred. **(A)** Network evidence plot. **(B)** Summary of relative effect sizes. Numbers in the light blue boxes are the values of SUCRA. Results with statistically significant differences are highlighted in bold. **(C)** Cumulative ranking probability plot.

For FEV_1_%pred, data from 57 RCTs were analyzed (Figure 4A). All ten combinations demonstrated statistically significant superiority over CT alone, except for SBP + CT (MD = 3.54, 95% CrI: −0.03 to 7.24). QQHT + CT (87.10%) was ranked as the most probable optimal intervention, followed by ECSZYQ + CT (81.70%) and DC + CT (69.00%) (Figure 4C). In network comparisons, QQHT + CT showed statistically significant superiority over SBP + CT and SZJQ + CT, but not over the remaining seven combinations (Figure 4B).

#### 3.5.3 FEV_1_/FVC

**FIGURE 5:**
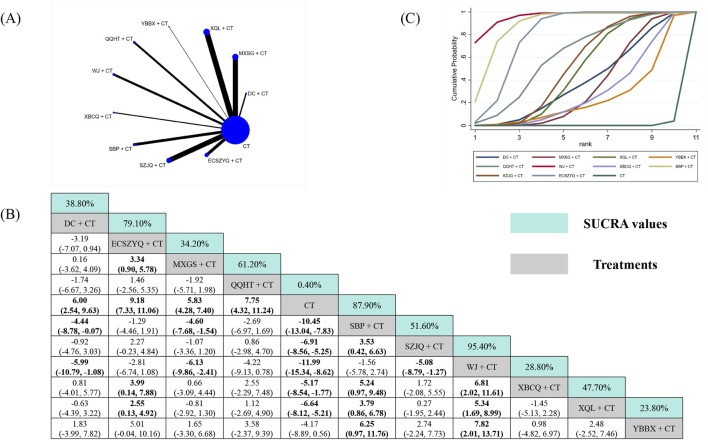
Network meta-analysis of FEV_1_/FVC. **(A)** Network evidence plot. **(B)** Summary of relative effect sizes. Numbers in the light blue boxes are the values of SUCRA. Results with statistically significant differences are highlighted in bold. **(C)** Cumulative ranking probability plot.

The analysis of FEV_1_/FVC included data from 101 RCTs (Figure 5A). Nine of the ten combinations resulted in significant improvements compared with CT alone. The exception was YBBX + CT (MD = 4.17, 95% CrI: −0.56 to 8.89), which did not reach statistical significance. WJ + CT (95.40%) was ranked as the most effective intervention, followed by SBP + CT (87.90%) and ECSZYQ + CT (79.10%) (Figure 5C).

Notably, WJ + CT showed statistically significant superiority over six of the other combinations, excluding ECSZYQ + CT, QQHT + CT, and SBP + CT (Figure 5B). The lack of significant differences among these four top-ranking interventions suggests their effects on FEV_1_/FVC may be comparable.

#### 3.5.4 PaO_2_

**FIGURE 6:**
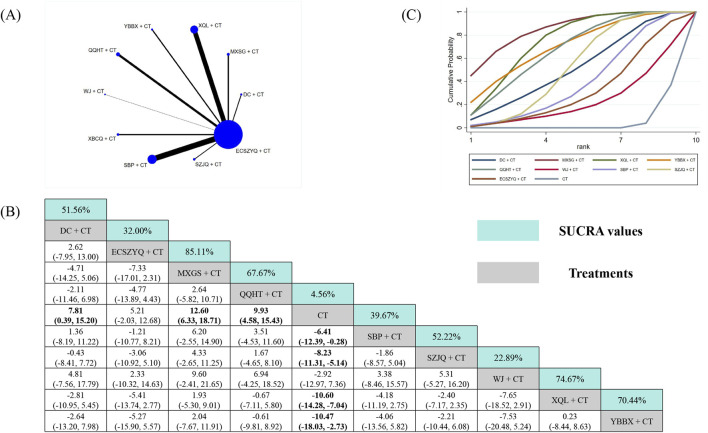
Network meta-analysis of PaO_2_. **(A)** Network evidence plot. **(B)** Summary of relative effect sizes. Numbers in the light blue boxes are the values of SUCRA. Results with statistically significant differences are highlighted in bold. **(C)** Cumulative ranking probability plot.

The PaO_2_ analysis incorporated data from 38 RCTs (Figure 6A). Seven combinations resulted in statistically significant improvements over CT alone. SUCRA ranking identified MXSG + CT (85.11%), XQL + CT (74.67%), and YBBX + CT (70.44%) as the most effective interventions (Figure 6C). Nevertheless, MXSG + CT did not demonstrate superiority over any other combinations in network comparisons (Figure 6B).

Additionally, ECSZYQ + CT (MD = 5.21, 95% CrI: −2.03 to 12.68) and WJ + CT (MD = 2.92, 95% CrI: −7.36 to 12.97) showed a trend toward improvement without statistical significance.

#### 3.5.5 PaCO_2_

**FIGURE 7:**
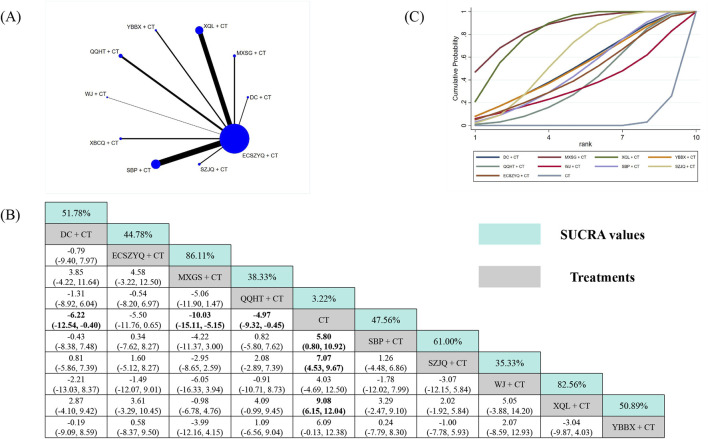
Network meta-analysis of PaCO_2_. **(A)** Network evidence plot. **(B)** Summary of relative effect sizes. Numbers in the light blue boxes are the values of SUCRA. Results with statistically significant differences are highlighted in bold. **(C)** Cumulative ranking probability plot.

The PaCO_2_ analysis included data from 38 RCTs (Figure 7A). Six combinations resulted in statistically significant reductions versus CT alone. SUCRA ranking positioned MXSG + CT (86.11%), XQL + CT (82.56%), and SZJQ + CT (61.00%) as the top interventions (Figure 7C). Mirroring the PaO_2_ findings, the highest-ranked MXSG + CT did not demonstrate superiority over other combinations (Figure 7B).

Conversely, three combinations failed to show significant reductions versus CT alone. Among them was YBBX + CT (MD = −6.09, 95% CrI: −12.38 to 0.13), which was ranked five overall. This discrepancy highlights that the SUCRA ranking is primarily driven by the point estimate. For YBBX + CT, the point estimate suggested a substantial effect, albeit with imprecision.

#### 3.5.6 Effective rate

**FIGURE 8:**
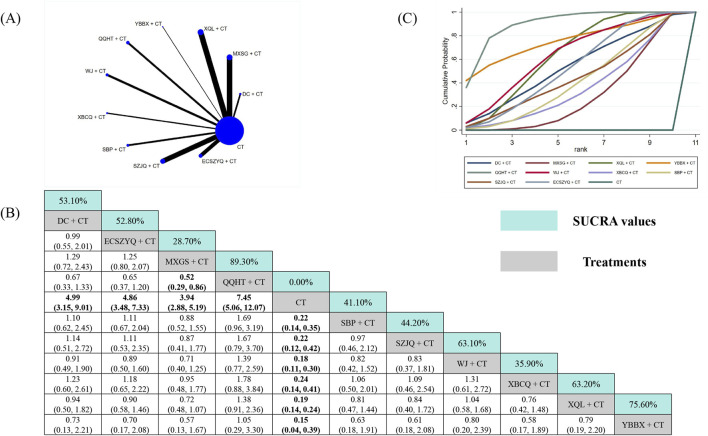
Network meta-analysis of effective rate. **(A)** Network evidence plot. **(B)** Summary of relative effect sizes. Numbers in the light blue boxes are the values of SUCRA. Results with statistically significant differences are highlighted in bold. **(C)** Cumulative ranking probability plot.

The effective rate analysis utilized data from 116 RCTs (Figure 8A). All ten combinations were significantly more effective than CT alone. SUCRA ranking identified QQHT + CT (89.30%) as the most effective intervention (Figure 8C). Based on the NMA estimates, it was statistically superior only to SBP + CT, but not over the other nine combinations, including the second-ranked YBBX + CT (75.60%) and third-ranked XQL + CT (63.20%) (Figure 8B).

### 3.6 Safety profile

Safety data were available from 39 RCTs involving 3,461 participants, which evaluated various herbal formulas (DC, ECSZYQ, MXSG, QQHT, SBP, SZJQ, WJ, XQL) combined with CT versus CT alone. Of these, 16 explicitly reported the absence of adverse events, while the remaining 23 documented at least one.

The most frequently reported adverse events involved gastrointestinal disorders (nausea, vomiting, diarrhea) and nervous system symptoms (headache, dizziness, sleep disturbances). Other reported events included palpitations, fatigue, rash, pruritus, dry mouth, and mild abnormalities in hepatic or renal function parameters. Most adverse events were mild in severity and resolved spontaneously or with minimal symptomatic intervention. A comprehensive summary of adverse events is provided in Supplementary Table A.19.

Meta-analysis of safety data revealed no statistically significant difference in overall adverse event incidence between combination therapy and CT alone (OR = 0.82, 95% CrI: 0.60 to 1.10). These findings suggest that the addition of herbal medicine to CT regimens does not significantly alter the safety profile.

### 3.7 Cluster analysis

To systematically characterize the multidimensional efficacy profiles of the interventions, we performed cluster analysis based on SUCRA values for key outcome pairs: FEV_1_ and FEV_1_%pred, FEV_1_ and FEV_1_/FVC, FEV_1_%pred and FEV_1_/FVC, as well as PaO_2_ and PaCO_2_. This analytic approach allowed us to identify interventions with similar response patterns across different physiological domains.

**FIGURE 9:**
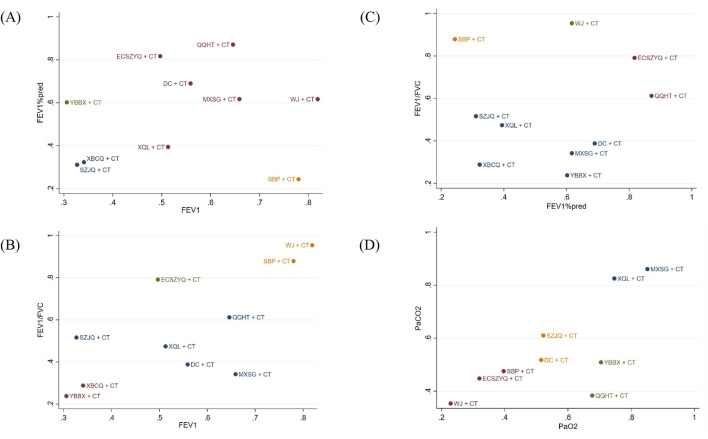
Clustering results for primary outcome indicators. **(A)** FEV_1_ and FEV_1_%pred. **(B)** FEV_1_ and FEV_1_/FVC. **(C)** FEV_1_%pred and FEV_1_/FVC. **(D)** PaO_2_ and PaCO_2_.

In the resulting cluster analysis diagram (Figure 9), interventions were grouped into four distinct clusters, each represented by a different color, illustrating their similarities and differences. According to the results, QQHT + CT, ECSZYQ + CT, and WJ + CT ranked highest for improving lung function. For the correction of arterial blood gas parameters, MXSG + CT, XQL + CT, and YBBX + CT were identified as the top-ranked interventions.

These findings indicate that a subset of interventions (such as QQHT + CT) demonstrate broad-spectrum efficacy across both lung function and arterial blood gas parameters, while some others exhibit more specialized therapeutic profiles.

### 3.8 Meta-regression

To explore potential sources of heterogeneity, univariate meta-regression was fitted with FEV_1_ as the dependent variable against the following pre-specified covariates: overall risk of bias, sample size (≤100 vs. >100), the composition of CT regimens, treatment duration (≤7, 8–14, >14 days), adherence to standard processing of core herbs (yes/no), herbal formula modification (unmodified, 1–3, >3 herbs), disease duration (≤7 vs. >7 years).

The results demonstrated no statistically significant association between the improvement in FEV_1_ and the following covariates: overall risk of bias, CT regimen, treatment duration, adherence to standard processing of core herbs, herbal formula modifications, and disease duration (P > 0.05). However, sample size may have been significantly associated with the FEV_1_ (P < 0.05) (Supplementary Figures A.2–A.8).

### 3.9 Subgroup analyses

Subgroup analyses were performed using the same variables as those employed in the meta-regression (Supplementary Tables A.20, A.21). The results demonstrated that for the comparison between SZJQ + CT and CT regarding FEV_1_/FVC, the subgroup of RCTs with a high overall risk of bias showed a smaller effect size (MD = 3.36), and the result was not statistically significant.

Evidence of effect modification by disease duration was observed. XQL + CT demonstrated greater improvement in FEV_1_/FVC among patients with longer disease duration (>7 years, MD = 7.41) compared to those with shorter duration (≤7 years, MD = 4.56). Conversely, MXSG + CT showed superior improvement in FEV_1_/FVC in the subgroup with shorter disease duration (≤7 years, MD = 5.95) relative to the longer duration subgroup (>7 years, MD = 3.84), with heterogeneity substantially reduced in both subgroups (I^2^ < 50%).

Analysis by sample size indicated small-study effects, as the subgroup of larger RCTs (>100 participants) yielded more conservative estimates for SZJQ + CT on both FEV_1_ (MD = 0.10) and FEV_1_/FVC (MD = 2.94).

Compared to the subgroup without standard processing (MD = 4.56), the subgroup that adopted standard processing of core herbs demonstrated a larger effect size (MD = 6.69) for XQL + CT, and the difference was statistically significant.

### 3.10 Sensitivity analyses

Sensitivity analyses were conducted to test robustness through four separate approaches: i. exclusion of high-risk RCTs; ii. restriction to RCTs with a consistent CT regimen (bronchodilators, antibiotics, and corticosteroids used concurrently); iii. restriction to RCTs using pharmacognostically defined herbal formulas, and iv. sequential exclusion of each high-risk RCT (leave-one-out analysis).

After excluding high-risk RCTs, NMA demonstrated minor changes in pooled effect sizes for all primary outcomes compared to the main analysis. The SUCRA rankings remained largely consistent, with the top-performing interventions unchanged across all endpoints (Supplementary Tables A.23–A.28).

Restricting to a consistent CT regimen, only pulmonary function outcomes could be assessed due to insufficient data on blood gas parameters. The top-three-ranked interventions for FEV_1_, FEV_1_%pred, and FEV_1_/FVC identified in the main analysis maintained their superior positions (Supplementary Tables A.29–A.31).

Restricting to pharmacognostically defined formulas limited the analysis to FEV_1_ data from 19 RCTs covering seven formulas (ECSZYQ, WJ, XBCQ were excluded due to insufficient studies). The intervention rankings from this subset analysis were largely consistent with those of the main analysis (Supplementary Table A.32).

Furthermore, after leave-one-out exclusion of high-risk RCTs, no significant inconsistency with the overall analysis was found.

### 3.11 Publication bias

Publication bias was assessed for FEV_1_, FEV_1_%pred, FEV_1_/FVC, PaO_2_, PaCO_2_, and the effective rate through Egger’s test. The results indicated significant publication bias for FEV_1_ (t = 7.461, P < 0.001), FEV_1_%pred (t = 2.268, P = 0.025), FEV_1_/FVC (t = 7.373, P < 0.001), PaCO_2_ (t = 4.605, P < 0.001), and the effective rate (t = −5.740, P < 0.001). PaO_2_ showed no significant publication bias (t = 1.335, P = 0.186). Funnel plots were generated to visually complement the statistical assessment of publication bias (Supplementary Figures A.9–A.14).

### 3.12 Certainty of evidence

The certainty of evidence for all PMAs was assessed using the GRADE method, with results showing a range of low to very low (Supplementary Table A.22). All 55 PMAs were downgraded by at least one level due to the risk of bias inherent in the included RCTs. Further downgrades were applied for heterogeneity (*I* ^*2*^ > 50%), imprecision (small sample sizes or wide CrIs), and publication bias. For FEV_1_, FEV_1_%pred, FEV_1_/FVC, PaO_2_, PaCO_2_, and effective rate, the proportions with “very low” certainty of evidence were 88.89% (8/9), 90.00% (9/10), 90.00% (9/10), 87.50% (7/8), 100% (8/8), and 10.00% (1/10), respectively.

For the NMA, the findings rely predominantly on indirect evidence due to the absence of head-to-head RCTs. This network structure precludes a statistical evaluation of inconsistency. As assessed by the GRADE-NMA methodology, the overall certainty of evidence was rated as “very low”, primarily due to the moderate to high risk of bias in the included RCTs, observable heterogeneity within the majority of direct comparisons, and imprecision in the effect estimates between herbal formulas.

## 4 Discussion

### 4.1 Summary of key findings

This comprehensive meta-analysis, integrating data from 132 RCTs involving 13,177 patients, demonstrates that the ten commonly used herbal formulas, when used as adjuncts to conventional therapy, are associated with superior clinical outcomes in AECOPD. Among them, QQHT and XQL were identified as the most promising candidates, owing to the superior efficacy of QQHT in improving lung function, the distinct advantage of XQL in correcting blood gas abnormalities, and the high rankings both achieved in the effective rate.

Specifically, QQHT, ECSZYQ and WJ exhibited the most pronounced effects in improving lung function, while MXSG, XQL, and YBBX ranked as the most promising for correcting blood gas abnormalities. Despite substantial heterogeneity, which may be attributable to methodological and clinical variations, the findings’ robustness was confirmed through extensive sensitivity analyses. It is critical to note, however, that the certainty of evidence for the NMA and most PMA outcomes was graded as “very low”.

The differential performance of certain herbal formulas across distinct clinical outcome domains warrants further elaboration. For example, WJ demonstrated a significant advantage on improving lung function, but its effect in improving arterial blood gas parameters was comparatively less favorable. This observation may be partly explained by imbalanced reporting frequencies of specific outcomes. Alternatively, it could reflect genuine differences in pharmacological composition and mechanisms of action. Although all formulas are indicated for AECOPD, they may target different pathophysiological pathways. This mechanistic diversity underscores the importance of tailoring therapy to individual patient presentation. While some formulas appear most suitable for broad-spectrum improvement, others may also be worth considering for patients with specific pathophysiological needs.

The distinct mechanistic profile of XBCQ exemplifies this principle and illustrates how TCM theory aligns with modern scientific understanding. As a representative treatment for COPD with lung-intestine heat excess syndrome, XBCQ has demonstrated significant efficacy in ameliorating both pulmonary inflammation and constipation. This dual effect is mediated by the modulation of gut microbiota (particularly a reduction in Escherichia-Shigella) and the regulation of key metabolic pathways, including linoleic acid metabolism and bile acid biosynthesis (Jiao et al., 2022). This finding provides a tangible molecular and microbiological basis for the TCM concept of “Exterior-Interior Relationship between the Lung and the Large Intestine,” positioning it within a modern context of a microbial-metabolic axis mechanism.

Another interesting finding was that the majority of comparisons between the herbal formulas yielded no statistically significant differences. This lack of differentiation may primarily be attributed to the similarities in the composition of these formulas. A prime example is Ephedra, which is a common ingredient in several formulas, including DC, MXSG, XQL, and YBBX. Despite this, the ranking capability of the NMA still provided valuable insights into their relative efficacy, revealing trends and highlighting the most promising candidates.

### 4.2 Comparison with previous studies

A previous network meta-analysis (Liu et al., 2019) evaluated the clinical efficacy of six herbal formulas for treating AECOPD patients. It is noteworthy that this study identified QQHT as a recommended intervention, which aligns with our findings. Detailed comparative data are available in Supplementary Table A.33.

It should be noted that this study did not account for variations in CT regimens or the potential influences of herbal processing methods and formula modifications. The quality of evidence reflects the credibility of treatment effect estimates (Huang et al., 2021). The study also suffered from insufficient statistical analysis and the absence of evidence quality assessment. These limitations introduced bias and undermined the reliability of the reported findings and conclusions.

In contrast, our study rigorously adhered to PRISMA guidelines and a predefined protocol to conduct an impartial, valid, and reliable network meta-analysis. Through meta-regression, subgroup analysis, and sensitivity analysis, we explored the sources of heterogeneity and validated the robustness of the results. The GRADE approach was employed to assess evidence quality, thereby providing a foundation for determining the strength of clinical recommendations.

### 4.3 Strengths and limitations

To our knowledge, this is the first systematic review to assess the therapeutic effects of the commonly used herbal formulas (traditional decoctions) for AECOPD. The analysis identified the most effective herbal formulas for improving pulmonary function, correcting blood gas abnormalities, and enhancing overall treatment outcomes. These findings address critical gaps in current research to a certain extent and highlight potential candidates that may guide clinical decision-making.

Furthermore, a comprehensive literature search and rigorous statistical methods were employed in this study, ensuring a robust synthesis and evaluation of the available evidence. Using the GRADE method, we revealed for the first time that the low certainty of evidence for the combination of herbal formulas with CT for the treatment of AECOPD may be related to the methodological flaws in RCTs and substantial publication bias. This finding characterizes the quality of the evidence supporting these intervention effects.

Notwithstanding these strengths, several limitations warrant consideration. Risk of bias assessment identified issues such as inadequate allocation concealment and insufficient blinding. Empirical studies have demonstrated that deficiencies in allocation concealment may inflate perceived treatment benefits by approximately 7%, while failure to maintain proper blinding has been associated with effect size exaggerations approaching 0.56 standard deviations (Yin et al., 2022; Kim et al., 2025). These biases may have contributed to a discernible overestimation of effects for observational clinical outcomes such as effective rates, whereas their influence on objective physiological measures like FEV_1_ is likely to be more modest. Although sensitivity analyses confirmed these methodological limitations did not substantially alter primary conclusions, they may nevertheless introduce non-negligible bias.

Despite an extensive literature search, all eligible studies were conducted in China. Therefore, the external validity of our findings may be limited by differences in healthcare systems, ethnic demographic profiles, and rates of adherence to conventional therapy (Al Wachami et al., 2024).

The observed publication bias may be attributed to multiple factors. Primarily and most fundamentally, the prevailing tendency in academia to preferentially publish positive results has directly resulted in a dearth of negative or null outcome data available for synthesis (Curry et al., 2025). Furthermore, the paucity of large-scale RCTs results in an evidence base dominated by smaller studies, which are particularly susceptible to publication bias (Yang et al., 2023). Additionally, our pre-defined inclusion criterion that excluded very small-sample studies, while methodologically justified for stability, may have further constrained the comprehensiveness of the analyzed evidence body, potentially interacting with publication bias. Consequently, the pooled efficacy estimates derived from this analysis should be interpreted with considerable caution, as they likely overestimate the true treatment effects.

The effective rate is a commonly used outcome measure in TCM clinical research in China. However, variability in reference standards across RCTs has led to inconsistent evaluation criteria, with some RCTs assessing efficacy using symptom score improvement rates and others employing composite indices incorporating additional laboratory parameters. This indicator is particularly susceptible to subjective influence from both investigators and participants, with its wide CIs indicating substantial measurement uncertainty (Zhang et al., 2025).

This NMA was primarily based on direct comparisons between herbal formulas combined with CT and the use of CT alone. Indirect comparisons of herbal formulas may result in imprecisions and non-transitivity of the findings (Ahn and Kang, 2021). This NMA employed Bayesian methods to compare the ten herbal formulas for the treatment of AECOPD. We will update this NMA using both frequentist and Bayesian approaches when better-designed RCTs become available, featuring larger sample sizes, higher quality, and direct comparisons of herbal formulas for efficacy and safety assessment.

### 4.4 Implication

In clinical trials of TCM, especially those involving herbal decoctions, blinding is often neglected due to practical difficulties, leading to methodological limitations and reduced study quality (Su et al., 2023). For future research, particular emphasis should be placed on the design of blinding procedures. Rigorous methodological design can effectively reduce bias and enhance the reliability and credibility of the results (Butcher et al., 2022).

The insufficient characterization of herbal preparations from a pharmacognostic perspective remains a significant obstacle in clinical research (Heinrich and Jalil, 2023). Key issues include incomplete reporting of material origin, inconsistent application of processing methods, and substantial variations in formula modifications and dosage regimens across studies, often reflecting the practice of pattern-based individualized treatments. These inconsistencies, stemming from a lack of authoritative standards for both starting materials and final preparations, fundamentally undermine the quality control and reproducibility of herbal formula research (Lu et al., 2022). To advance the field, a critical next step is the establishment of internationally accepted, consensus-based standards that provide precise pharmacognostic specifications for material origin, processing, modifications, and dosage ranges.

The decoction process is one of the key factors influencing the pharmacological efficacy of herbal formulas. However, due to the long-standing absence of standardized decoction protocols, this lack introduces the risk of variations in parameters (duration, heat intensity, water-to-herb ratio) across studies. Of the 132 included studies, only 34 (25.76%) reported decoction preparation details. Recently, a Chinese expert panel established the “Technical Specification for Preparation of Standard Decoction of Chinese Medicines” (No. T/CACM 1572-2024). This specification sets, for the first time, clear and detailed quantifiable standards for these critical parameters. Future studies should rigorously adhere to this protocol and explicitly report decoction conditions to enhance reproducibility and scientific rigor of research findings.

Beyond surrogate laboratory markers, future trials should prioritize severely underreported, patient-centered outcomes, including exacerbation frequency, hospitalization length, readmission rates, and quality of life. Concurrently, safety reporting requires major improvement, as evidenced by the absence of data in over 70% of the included studies. Notably, among those that did, more than 40% reported zero adverse events. This finding raises concerns about potential inconsistencies in the monitoring or reporting of adverse events. It must be emphasized that safety and long-term therapeutic effects are vital components of a comprehensive intervention assessment.

Given the differences in physiological characteristics and disease manifestations across regions and populations, large-scale, multicenter research in diverse populations is crucial (Editors and Rubin, 2021). Furthermore, there is a critical need for head-to-head trials to directly compare the most promising herbal formulas identified in our analysis, thereby providing clinicians with definitive evidence for optimal treatment selection.

## 5 Conclusion

Based on their favorable rankings, this NMA indicates that QQHT and XQL could be the preferred therapeutic options among the evaluated herbal formulas. However, given the low certainty of evidence, clinical application of these findings should be cautious and take into account the patient’s specific pathophysiological profile and individual factors. Ultimately, the relative efficacy and safety of these formulas require validation through future rigorously designed, large-scale, head-to-head randomized trials. Such trials should prioritize patient-centered outcomes and adhere to standardized preparation protocols, especially when focusing on the higher-ranked candidates.

## Data Availability

The original contributions presented in the study are included in the article/Supplementary Material, further inquiries can be directed to the corresponding author.
