# Supplementary material for: Comparative effectiveness of ten traditional Chinese herbal formulas for acute exacerbation of chronic obstructive pulmonary disease: a systematic review and Bayesian network meta-analysis
Source: Front Pharmacol. 2026 Jan 15;16:1585150. doi: 10.3389/fphar.2025.1585150 (PMC12852464; doi:10.3389/fphar.2025.1585150)

Appendix

Appendix Table A.1–A.2: PRISMA checklist 3
Appendix Table A.1: PRISMA 2020 checklist 3
Appendix Table A.2: PRISMA-NMA checklist 6
Appendix Table A.3: Basic information of drug composition in ten herbal formulas 10
Appendix Table A.4: Standard processing methods of herbs 15
Appendix Table A.5 Taxonomically verified scientific nomenclature of all cited species 16
Appendix Table A.6–A.14: Search Strategy 18
Table A.6: Search Strategy: PubMed 18
Table A.7: Search Strategy: Embase 18
Table A.8: Search Strategy: Cochrane Central Register of Controlled Trials (CENTRAL) 19
Table A.9: Search Strategy: Cumulative Index to Nursing and Allied Health Literature (CINAHL) 19
Table A.10: Search Strategy: Web of Science 19
Table A.11: Search Strategy: Chinese Biomedical Database (CBM) 20
Table A.12: Search Strategy: China National Knowledge Infrastructure (CNKI) 20
Table A.13: Search Strategy: Wanfang Data Knowledge Service Platform (Wanfang Database) 20
Table A.14: Search Strategy: Chongqing VIP Information Chinese Science and Technology Journal Database (VIP Database) 20
Appendix Table A.15: Characteristics of included studies 21
Appendix Table A.16: Herb details in included studies 31
Appendix Table A.17: Decoction protocols for herbal formulas in the included studies 38
Appendix Table A.18: Pairwise random-effects meta-analyses for all outcomes 39
Appendix Table A.19: Summary of adverse events 40
Appendix Table A.20–A.21: Subgroup analysis 42
Appendix Table A.20: Summary of subgroups with significant between-group effect size difference 42
Appendix Table A.21: Summary of subgroups where stratification reduced overall heterogeneity 43
Appendix Table A.22: Evidence strengths of pairwise meta-analyses with the GRADE approach 44
Appendix Table A.23–A.28: Sensitivity analysis by excluding all high-risk RCTs 47
Appendix Table A.23: FEV_1 47
Appendix Table A.24: FEV_1%pred 48
Appendix Table A.25: FEV_1/FVC 49
Appendix Table A.26: PaO_2 50
Appendix Table A.27: $PaCO_2$ 51
Appendix Table A.28: Effective rate 52
Appendix Table A.29–A.31: Sensitivity analysis by protocol-defined CT regimen 53

Appendix Table A.29: FEV_1 53
Appendix Table A.30: FEV_1%pred 54
Appendix Table A.31: FEV_1/FVC 55
Appendix Table A.32: Sensitivity analysis of FEV_1 by consistent pharmacognostic definition 56
Appendix Table A.33: Comparison with Previous network meta-analysis 57
Appendix Table A.34–A.35: Raw data of outcome measures after treatment 59
Appendix Table A.34. FEV_1, FEV_1%pred, and FEV_1/FVC data 59
Appendix Table A.35. PaO_2, $PaCO_2$, and effective rate data 62
Appendix Table A.36. Program code for network meta-analysis using WinBUGS 65
Appendix Fig A.1 Bias risk assessment of included studies 74
Appendix Fig A.2–A.9 Meta-regression analysis 75
Appendix Fig A.2 Overall risk of bias 75
Appendix Fig A.3 Sample size 75
Appendix Fig A.4 Composition of the CT regimen 76
Appendix Fig A.5 Treatment duration 76
Appendix Fig A.6 Adherence to standard processing of core herbs 77
Appendix Fig A.7 Herbal formula modification 77
Appendix Fig A.8 Disease duration 78
Appendix Fig A.9–A.14 Funnel plots 79
Appendix Fig A.9 FEV_1 79
Appendix Fig A.10 FEV_1%pred 79
Appendix Fig A.11 FEV_1/FVC 80
Appendix Fig A.12 PaO_2 80
Appendix Fig A.13 $PaCO_2$ 81
Appendix Fig A.14 Effective rate 81

Abbreviation explanation of traditional Chinese herbal formulas in this appendix:
DC: Dingchuan decoction; MXSG: Maxingshigan decoction; XQL: Xiaoqinglong decoction; YBBX: Yuebi Jia Banxia decoction; QQHT: Qingqihuatan decoction; WJ: Weijing decoction; XBCQ: Xuanbaichengqi decoction; SBP: Sangbaipi decoction; SZJQ: Suzijiangqi decoction; ECSZYQ: Erchen decoction combined with Sanziyangqin decoction.

Appendix Table A.1–A.2: PRISMA checklist

Appendix Table A.1: PRISMA 2020 checklist

Section and Topic	Item #	Checklist item	Location where item is reported
**TITLE**			
Title	1	Identify the report as a systematic review.	Title
**ABSTRACT**			
Abstract	2	See the PRISMA 2020 for Abstracts checklist.	Abstract
**INTRODUCTION**			
Rationale	3	Describe the rationale for the review in the context of existing knowledge.	Introduction
Objectives	4	Provide an explicit statement of the objective(s) or question(s) the review addresses.	Introduction
**METHODS**			
Eligibility criteria	5	Specify the inclusion and exclusion criteria for the review and how studies were grouped for the syntheses.	2.4 Inclusion and exclusion criteria
Information sources	6	Specify all databases, registers, websites, organisations, reference lists and other sources searched or consulted to identify studies. Specify the date when each source was last searched or consulted.	2.3 Literature search
Search strategy	7	Present the full search strategies for all databases, registers and websites, including any filters and limits used.	2.3 Literature search
Selection process	8	Specify the methods used to decide whether a study met the inclusion criteria of the review, including how many reviewers screened each record and each report retrieved, whether they worked independently, and if applicable, details of automation tools used in the process.	2.5 Study selection and data extraction
Data collection process	9	Specify the methods used to collect data from reports, including how many reviewers collected data from each report, whether they worked independently, any processes for obtaining or confirming data from study investigators, and if applicable, details of automation tools used in the process.	2.5 Study selection and data extraction
Data items	10a	List and define all outcomes for which data were sought. Specify whether all results that were compatible with each outcome domain in each study were sought (e.g. for all measures, time points, analyses), and if not, the methods used to decide which results to collect.	2.5 Study selection and data extraction
	10b	List and define all other variables for which data were sought (e.g. participant and intervention characteristics, funding sources). Describe any assumptions made about any missing or unclear information.	2.5 Study selection and data extraction
Study risk of bias assessment	11	Specify the methods used to assess risk of bias in the included studies, including details of the tool(s) used, how many reviewers assessed each study and whether they worked independently, and if applicable, details of automation tools used in the process.	2.6 Risk of bias assessment and certainty of evidence
Effect measures	12	Specify for each outcome the effect measure(s) (e.g. risk ratio, mean difference) used in the synthesis or presentation of results.	2.7 Data synthesis and analysis

Section and Topic	Item #	Checklist item	Location where item is reported
Synthesis methods	13a	Describe the processes used to decide which studies were eligible for each synthesis (e.g. tabulating the study intervention characteristics and comparing against the planned groups for each synthesis (item #5)).	2.7 Data synthesis and analysis
	13b	Describe any methods required to prepare the data for presentation or synthesis, such as handling of missing summary statistics, or data conversions.	2.7 Data synthesis and analysis
	13c	Describe any methods used to tabulate or visually display results of individual studies and syntheses.	2.7 Data synthesis and analysis
	13d	Describe any methods used to synthesize results and provide a rationale for the choice(s). If meta-analysis was performed, describe the model(s), method(s) to identify the presence and extent of statistical heterogeneity, and software package(s) used.	2.7 Data synthesis and analysis
	13e	Describe any methods used to explore possible causes of heterogeneity among study results (e.g. subgroup analysis, meta-regression).	2.7 Data synthesis and analysis
	13f	Describe any sensitivity analyses conducted to assess robustness of the synthesized results.	2.7 Data synthesis and analysis
Reporting bias assessment	14	Describe any methods used to assess risk of bias due to missing results in a synthesis (arising from reporting biases).	2.7 Data synthesis and analysis
Certainty assessment	15	Describe any methods used to assess certainty (or confidence) in the body of evidence for an outcome.	2.6 Risk of bias assessment and certainty of evidence
**RESULTS**			
Study selection	16a	Describe the results of the search and selection process, from the number of records identified in the search to the number of studies included in the review, ideally using a flow diagram.	3.1 Study selection
	16b	Cite studies that might appear to meet the inclusion criteria, but which were excluded, and explain why they were excluded.	3.1 Study selection
Study characteristics	17	Cite each included study and present its characteristics.	Appendix Table A.15
Risk of bias in studies	18	Present assessments of risk of bias for each included study.	3.3 Quality assessment
Results of individual studies	19	For all outcomes, present, for each study: (a) summary statistics for each group (where appropriate) and (b) an effect estimate and its precision (e.g. confidence/credible interval), ideally using structured tables or plots.	Appendix Table A.34–A.35
Results of syntheses	20a	For each synthesis, briefly summarise the characteristics and risk of bias among contributing studies.	Results
	20b	Present results of all statistical syntheses conducted. If meta-analysis was done, present for each the summary estimate and its precision (e.g. confidence/credible interval) and measures of statistical heterogeneity. If comparing groups, describe the direction of the effect.	Results
	20c	Present results of all investigations of possible causes of heterogeneity among study results.	3.8 Meta-regression,

Section and Topic	Item #	Checklist item	Location where item is reported
			3.9 Subgroup analyses
	20d	Present results of all sensitivity analyses conducted to assess the robustness of the synthesized results.	3.10 Sensitivity analyses
Reporting biases	21	Present assessments of risk of bias due to missing results (arising from reporting biases) for each synthesis assessed.	3.11 Publication bias
Certainty of evidence	22	Present assessments of certainty (or confidence) in the body of evidence for each outcome assessed.	3.12 Certainty of evidence
**DISCUSSION**			
Discussion	23a	Provide a general interpretation of the results in the context of other evidence.	4.1 Summary of key findings
	23b	Discuss any limitations of the evidence included in the review.	4.3 Strengths and limitations
	23c	Discuss any limitations of the review processes used.	4.3 Strengths and limitations
	23d	Discuss implications of the results for practice, policy, and future research.	4.4 Implication
**OTHER INFORMATION**			
Registration and protocol	24a	Provide registration information for the review, including register name and registration number, or state that the review was not registered.	2.1 Systematic review registration
	24b	Indicate where the review protocol can be accessed, or state that a protocol was not prepared.	2.1 Systematic review registration
	24c	Describe and explain any amendments to information provided at registration or in the protocol.	2.1 Systematic review registration
Support	25	Describe sources of financial or non-financial support for the review, and the role of the funders or sponsors in the review.	Funding
Competing interests	26	Declare any competing interests of review authors.	Conflict of interest
Availability of data, code and other materials	27	Report which of the following are publicly available and where they can be found: template data collection forms; data extracted from included studies; data used for all analyses; analytic code; any other materials used in the review.	Data availability statement

Appendix Table A.2: PRISMA-NMA checklist

Section/Topic	Item #	Checklist Item	Reported on Page #
**TITLE**			
Title	1	Identify the report as a systematic review *incorporating a network meta-analysis (or related form of meta-analysis).*	Title
**ABSTRACT**			
Structured summary	2	Provide a structured summary including, as applicable:<br>**Background:** main objectives<br>**Methods:** data sources; study eligibility criteria, participants, and interventions; study appraisal; and *synthesis methods, such as network meta-analysis.*<br>**Results:** number of studies and participants identified; summary estimates with corresponding confidence/credible intervals; *treatment rankings may also be discussed. Authors may choose to summarize pairwise comparisons against a chosen treatment included in their analyses for brevity.*<br>**Discussion/Conclusions:** limitations; conclusions and implications of findings.<br>**Other:** primary source of funding; systematic review registration number with registry name.	Abstract
**INTRODUCTION**			
Rationale	3	Describe the rationale for the review in the context of what is already known*, including mention of why a network meta-analysis has been conducted.*_	Introduction
Objectives	4	Provide an explicit statement of questions being addressed, with reference to participants, interventions, comparisons, outcomes, and study design (PICOS).	Introduction
**METHODS**			
Protocol and registration	5	Indicate whether a review protocol exists and if and where it can be accessed (e.g., Web address); and, if available, provide registration information, including registration number.	2.1 Systematic review registration
Eligibility criteria	6	Specify study characteristics (e.g., PICOS, length of follow-up) and report characteristics (e.g., years considered, language, publication status) used as criteria for eligibility, giving rationale. *Clearly describe eligible treatments included in the treatment network, and note whether any have been clustered or merged into the same node (with justification).*_	2.4 Inclusion and exclusion criteria

Information sources	7	Describe all information sources (e.g., databases with dates of coverage, contact with study authors to identify additional studies) in the search and date last searched.	2.3 Literature search
Search	8	Present full electronic search strategy for at least one database, including any limits used, such that it could be repeated.	Appendix Table A.6–A.14
Study selection	9	State the process for selecting studies (i.e., screening, eligibility, included in systematic review, and, if applicable, included in the meta-analysis).	2.5 Study selection and data extraction
Data collection process	10	Describe method of data extraction from reports (e.g., piloted forms, independently, in duplicate) and any processes for obtaining and confirming data from investigators.	2.5 Study selection and data extraction
Data items	11	List and define all variables for which data were sought (e.g., PICOS, funding sources) and any assumptions and simplifications made.	2.5 Study selection and data extraction
**Geometry of the network**	**S1**	Describe methods used to explore the geometry of the treatment network under study and potential biases related to it. This should include how the evidence base has been graphically summarized for presentation, and what characteristics were compiled and used to describe the evidence base to readers.	2.7 Data synthesis and analysis
Risk of bias within individual studies	12	Describe methods used for assessing risk of bias of individual studies (including specification of whether this was done at the study or outcome level), and how this information is to be used in any data synthesis.	2.6 Risk of bias assessment and certainty of evidence
Summary measures	13	State the principal summary measures (e.g., risk ratio, difference in means). *Also describe the use of additional summary measures assessed, such as treatment rankings and surface under the cumulative ranking curve (SUCRA) values, as well as modified approaches used to present summary findings from meta-analyses.*	2.7 Data synthesis and analysis
Planned methods of analysis	14	Describe the methods of handling data and combining results of studies for each network meta-analysis. This should include, but not be limited to:<br>• *Handling of multi-arm trials;*<br>• *Selection of variance structure;*<br>• *Selection of prior distributions in Bayesian analyses; and*<br>• *Assessment of model fit.*	2.7 Data synthesis and analysis
**Assessment of Inconsistency**	**S2**	Describe the statistical methods used to evaluate the agreement of direct and indirect evidence in the treatment network(s) studied. Describe efforts taken to address its presence when found.	2.7 Data synthesis and analysis
Risk of bias across studies	15	Specify any assessment of risk of bias that may affect the cumulative evidence (e.g., publication bias, selective reporting within studies).	2.7 Data synthesis and analysis
Additional analyses	16	Describe methods of additional analyses if done, indicating which were pre-specified. This may include, but not be limited to, the	2.7 Data synthesis and analysis

		following:<br>• Sensitivity or subgroup analyses;<br>• Meta-regression analyses;<br>• *Alternative formulations of the treatment network; and*<br>• *Use of alternative prior distributions for Bayesian analyses (if applicable).*_	
**RESULTS†**			
Study selection	17	Give numbers of studies screened, assessed for eligibility, and included in the review, with reasons for exclusions at each stage, ideally with a flow diagram.	3.1 Study selection
**Presentation of network structure**	**S3**	Provide a network graph of the included studies to enable visualization of the geometry of the treatment network.	Figure 3C–8C
**Summary of network geometry**	**S4**	Provide a brief overview of characteristics of the treatment network. This may include commentary on the abundance of trials and randomized patients for the different interventions and pairwise comparisons in the network, gaps of evidence in the treatment network, and potential biases reflected by the network structure.	3.2 Characteristics of included studies
Study characteristics	18	For each study, present characteristics for which data were extracted (e.g., study size, PICOS, follow-up period) and provide the citations.	Supplementary Appendix Table A.15
Risk of bias within studies	19	Present data on risk of bias of each study and, if available, any outcome level assessment.	3.3 Quality assessment
Results of individual studies	20	For all outcomes considered (benefits or harms), present, for each study: 1) simple summary data for each intervention group, and 2) effect estimates and confidence intervals. *Modified approaches may be needed to deal with information from larger networks.*	Supplementary Appendix Table A.34–A.35
Synthesis of results	21	Present results of each meta-analysis done, including confidence/credible intervals. *In larger networks, authors may focus on comparisons versus a particular comparator (e.g. placebo or standard care), with full findings presented in an appendix. League tables and forest plots may be considered to summarize pairwise comparisons.* If additional summary measures were explored (such as treatment rankings), these should also be presented.	Results
**Exploration for inconsistency**	**S5**	Describe results from investigations of inconsistency. This may include such information as measures of model fit to compare consistency and inconsistency models, *P* values from statistical tests, or summary of inconsistency estimates from different parts of	3.5 The results of NMA

		the treatment network.	
Risk of bias across studies	22	Present results of any assessment of risk of bias across studies for the evidence base being studied.	3.12 Certainty of evidence
Results of additional analyses	23	Give results of additional analyses, if done (e.g., sensitivity or subgroup analyses, meta-regression analyses*, alternative network geometries studied, alternative choice of prior distributions for Bayesian analyses,* and so forth).	Results
**DISCUSSION**			
Summary of evidence	24	Summarize the main findings, including the strength of evidence for each main outcome; consider their relevance to key groups (e.g., healthcare providers, users, and policy-makers).	4.1 Summary of key findings
Limitations	25	Discuss limitations at study and outcome level (e.g., risk of bias), and at review level (e.g., incomplete retrieval of identified research, reporting bias). *Comment on the validity of the assumptions, such as transitivity and consistency. Comment on any concerns regarding network geometry (e.g., avoidance of certain comparisons).*	4.3 Strengths and limitations
Conclusions	26	Provide a general interpretation of the results in the context of other evidence, and implications for future research.	Discussion
**FUNDING**			
Funding	27	Describe sources of funding for the systematic review and other support (e.g., supply of data); role of funders for the systematic review. This should also include information regarding whether funding has been received from manufacturers of treatments in the network and/or whether some of the authors are content experts with professional conflicts of interest that could affect use of treatments in the network.	Funding

PICOS = population, intervention, comparators, outcomes, study design.
* Text in italics indicates wording specific to reporting of network meta-analyses that has been added to guidance from the PRISMA statement.
† Authors may wish to plan for use of appendices to present all relevant information in full detail for items in this section.

Appendix Table A.3: Basic information of drug composition in ten herbal formulas

Formula name	Common English name	Latin name	Chinese name	Authorities	Family	Genus	Medicinal part	Initial Preparation	Main bioactive compounds	Dosage (grams)	Medicinal source (Pharmacopoeia)	Level of reporting in the original study
	Ginkgo Seed	*Ginkgo biloba* L.	Bai Guo	Carl Linnaeus	Ginkgoaceae	Ginkgo	Dried Seed	Remove impurities and hard calyx, crush before use	Ginkgolides (A, B, C, J, M), Bilobalides, Flavonoid glycosides (quercetin-3-O-glucoside, kaempferol-3-O-glucoside)	9	China Pharmacopoeia (2020)	Inadequate
	Ephedra Herb	*Ephedra sinica* Stapf	Ma Huang	Otto Stapf	Ephedraceae	Ephedra	Herbaceous Stem	Remove the woody stems, root remnants, and impurities; cut into sections	Ephedrine alkaloids (ephedrine, pseudoephedrine, norephedrine, norpseudoephedrine, methylephedrine)	6	China Pharmacopoeia (2020)	Inadequate
	Perilla Seed	*Perilla frutescens* (L.) Britt.	Zi Su Zi	Carl Linnaeus, Nathaniel Lord Britton	Lamiaceae	Perilla	Dried Seed	Remove impurities, wash clean, and dry	α-Linolenic acid, Perilla saponins (I-IV), Perilla ketone, Flavonoids (apigenin, luteolin)	9	China Pharmacopoeia (2020)	Inadequate
	Licorice	*Glycyrrhiza uralensis* Fisch.	Gan Cao	Friedrich Ernst Ludwig von Fischer	Fabaceae	Glycyrrhiza	Dried root and rhizome	Remove impurities, wash clean, and dry	Glycyrrhizin, Glycyrrhetic acid, Liquiritin, Liquiritigenin, Isoliquiritin, Licoricidin, Glycyrol	6	China Pharmacopoeia (2020)	Inadequate
**DC**	Coltsfoot Flower	*Tussilago farfara* L.	Kuan Dong Hua	Carl Linnaeus	Asteraceae	Tussilago	Flower Bud	Remove impurities and dry in shade	Faradiol, Flavonoids (rutin, quercetin, luteolin), Chlorogenic acid, Germacrene D	9	China Pharmacopoeia (2020)	Inadequate
	Bitter Apricot Seed	*Prunus armeniaca* L. var. *ansu* Maxim.	Ku Xing Ren	Carl Linnaeus, Karl Ivanovich Maximowicz	Rosaceae	Prunus	Seed	Remove impurities	Amygdalin, Amygdalin saponins, Quercetin, Oleic acid, Linoleic acid	9	China Pharmacopoeia (2020)	Inadequate
	White Mulberry Root Bark	*Morus alba* L.	Sang Bai Pi	Carl Linnaeus	Moraceae	Morus	Root Bark	Remove coarse bark, wash clean and dry	Mulberroside A, Oxyresveratrol, Kuwanon G, Morin, 1-Deoxynojirimycin	9	China Pharmacopoeia (2020)	Inadequate
	Baical Skullcap Root	*Scutellaria baicalensis* Georgi	Huang Qin	Johann Gottlieb Georgi	Lamiaceae	Scutellaria	Root	Remove impurities, boil in water for 10 minutes, take out, stew to soften, cut into thin slices, and dry.	Baicalin, Baicalein, Wogonin, Wogonoside, Oroxylin A, Scutellarin	9	China Pharmacopoeia (2020)	Inadequate
	Pinellia Tuber	*Pinellia ternata* (Thunb.) Breit.	Ban Xia	Carl Peter Thunberg, Karl (Max) Breiter	Araceae	Pinellia	Tuber	Crush before use	Pinellinine, L-(-)-N-methylguvacine, Tuberostemonine, p-Hydroxybenzoic acid, Methylparaben	9	China Pharmacopoeia (2020)	Inadequate
	Ephedra Herb	*Ephedra sinica* Stapf	Ma Huang	Otto Stapf	Ephedraceae	Ephedra	Herbaceous Stem	Remove the woody stems, root remnants, and impurities; cut into sections	Ephedrine alkaloids (ephedrine, pseudoephedrine, norephedrine, norpseudoephedrine, methylephedrine)	9	China Pharmacopoeia (2020)	Inadequate
**MXSG**	Bitter Apricot Seed	*Prunus armeniaca L.* var. *ansu* Maxim.	Ku Xing Ren	Carl Linnaeus, Karl Ivanovich Maximowicz	Rosaceae	Prunus	Seed	Remove impurities	Amygdalin, Amygdalin saponins, Quercetin, Oleic acid, Linoleic acid	9	China Pharmacopoeia (2020)	Inadequate
	Gypsum	Gypsum Fibrosum	Shi Gao	Mineral (no botanical authority)	N/A	N/A	Mineral	Crush into powder	Calcium sulfate dihydrate ($CaSO_4 \cdot 2H_2O$), Trace elements (Mg, Al, Fe, K)	18	China Pharmacopoeia (2020)	Inadequate
	Licorice	*Glycyrrhiza uralensis* Fisch.	Gan Cao	Friedrich Ernst Ludwig von Fischer	Fabaceae	Glycyrrhiza	Dried root and rhizome	Remove impurities, wash clean, and dry	Glycyrrhizin, Glycyrrhetic acid, Liquiritin, Liquiritigenin, Isoliquiritin, Licoricidin, Glycyrol	6	China Pharmacopoeia (2020)	Inadequate
	Ephedra Herb	*Ephedra sinica* Stapf	Ma Huang	Otto Stapf	Ephedraceae	Ephedra	Herbaceous Stem	Remove the woody stems, root remnants, and impurities; cut into sections	Ephedrine alkaloids (ephedrine, pseudoephedrine, norephedrine, norpseudoephedrine, methylephedrine)	9	China Pharmacopoeia (2020)	Inadequate
	Manchurian Wild Ginger	*Asarum heterotropoides* F.Schmidt	Xi Xin	Friedrich Schmidt	Aristolochiaceae	Asarum	Root and Rhizome	Clean and dry	Methyl eugenol, Aristolochic acid I, Aristolactam I, Eugenol, Safrole	3	China Pharmacopoeia (2020)	Inadequate

	Cinnamon Twig	*Cinnamomum cassia* (L.) J. Presl	Gui Zhi	Carl Linnaeus	Lauraceae	Cinnamomum	Dried young branches	Remove impurities, wash clean, moisten thoroughly, cut into thick slices, and dry	Cinnamaldehyde, Cinnamic acid, Eugenol, Quercetin, Ursolic acid	9	China Pharmacopoeia (2020)	Inadequate
**XQL**	White Peony Root	*Paeonia lactiflora* Pall.	Bai Shao	Peter Simon Pallas	Paeoniaceae	Paeonia	Root	Wash clean, moisten thoroughly, cut into thin slices, and dry.	Paeoniflorin, Albiflorin, Oxypaeoniflorin, Benzoylpaeoniflorin, Paeonol	9	China Pharmacopoeia (2020)	Inadequate
	Licorice	*Glycyrrhiza uralensis* Fisch.	Gan Cao	Friedrich Ernst Ludwig von Fischer	Fabaceae	Glycyrrhiza	Dried root and rhizome	Remove impurities, wash clean, and dry	Glycyrrhizin, Glycyrrhetic acid, Liquiritin, Liquiritigenin, Isoliquiritin, Licoricidin, Glycyrol	6	China Pharmacopoeia (2020)	Inadequate
	Dried Ginger	*Zingiber officinale* Roscoe	Gan Jiang	William Roscoe	Zingiberaceae	Zingiber	Rhizome	Clean, slice, and dry	6-Gingerol, 8-Gingerol, 10-Gingerol, Zingiberene, Shogaol, Zingerone	9	China Pharmacopoeia (2020)	Inadequate
	Pinellia Tuber	*Pinellia ternata* (Thunb.) Breit.	Ban Xia	Carl Peter Thunberg, Karl (Max) Breiter	Araceae	Pinellia	Tuber	Crush before use	Pinellinine, L-(-)-N-methylguvacine, Tuberostemonine, p-Hydroxybenzoic acid, Methylparaben	9	China Pharmacopoeia (2020)	Inadequate
	Chinese Magnolia Vine Fruit	*Schisandra chinensis* (Turcz.) Baill.	Wu Wei Zi	Nikolai Turczaninow, Henri Ernest Baillon	Schisandraceae	Schisandra	Fruit	Clean and dry	Schisandrin A, Schisandrin B, Schisandrol A, Schisandrol B, Schisantherin A, Deoxyschisandrin	6	China Pharmacopoeia (2020)	Inadequate
	Ephedra Herb	*Ephedra sinica* Stapf	Ma Huang	Otto Stapf	Ephedraceae	Ephedra	Herbaceous Stem	Remove the woody stems, root remnants, and impurities; cut into sections	Ephedrine alkaloids (ephedrine, pseudoephedrine, norephedrine, norpseudoephedrine, methylephedrine)	9	China Pharmacopoeia (2020)	Inadequate
	Gypsum	Gypsum Fibrosum	Shi Gao	Mineral (no botanical authority)	N/A	N/A	Mineral	Crush into powder	Calcium sulfate dihydrate ($CaSO_4 \cdot 2H_2O$), Trace elements (Mg, Al, Fe, K)	30	China Pharmacopoeia (2020)	Inadequate
**YBBX**	Fresh Ginger	*Zingiber officinale* Roscoe	Sheng Jiang	William Roscoe	Zingiberaceae	Zingiber	Fresh Rhizome	Clean and slice	6-Gingerol, Zingiberene, Zingerone, Shogaol, Paradol	9	China Pharmacopoeia (2020)	Inadequate
	Jujube Fruit	*Ziziphus jujuba* Mill.	Da Zao	Philip Miller	Rhamnaceae	Ziziphus	Fruit	Clean and dry	Jujube polysaccharides, Hesperidin, Ursolic acid, Quercetin, Vitamin C	5 pieces	China Pharmacopoeia (2020)	Inadequate
	Licorice	*Glycyrrhiza uralensis* Fisch.	Gan Cao	Friedrich Ernst Ludwig von Fischer	Fabaceae	Glycyrrhiza	Dried root and rhizome	Remove impurities, wash clean, and dry	Glycyrrhizin, Glycyrrhetic acid, Liquiritin, Liquiritigenin, Isoliquiritin, Licoricidin, Glycyrol	6	China Pharmacopoeia (2020)	Inadequate
	Pinellia Tuber	*Pinellia ternata* (Thunb.) Breit.	Ban Xia	Carl Peter Thunberg, Karl (Max) Breiter	Araceae	Pinellia	Tuber	Crush before use	Pinellinine, L-(-)-N-methylguvacine, Tuberostemonine, p-Hydroxybenzoic acid, Methylparaben	12	China Pharmacopoeia (2020)	Inadequate
	Aged Tangerine Peel	*Citrus reticulata* Blanco 'Chachi'	Chen Pi	Francisco Manuel Blanco	Rutaceae	Citrus	Dried pericarp of mature fruit	Clean, sun-dry, and store for aging	D-Limonene, Hesperidin, Neohesperidin, Scopoletin, Ursolic acid	9	China Pharmacopoeia (2020)	Inadequate
	Bitter Apricot Seed	*Prunus armeniaca* L. var. *ansu* Maxim.	Ku Xing Ren	Carl Linnaeus, Karl Ivanovich Maximowicz	Rosaceae	Prunus	Seed	Remove impurities	Amygdalin, Amygdalin saponins, Quercetin, Oleic acid, Linoleic acid	9	China Pharmacopoeia (2020)	Inadequate
	Immature Bitter Orange Fruit	*Citrus aurantium* L.	Zhi Shi	Carl Linnaeus	Rutaceae	Citrus	Immature Fruit	Clean, slice, and dry	Synephrine, Neohesperidin, Naringin, D-Limonene, Octopamine	9	China Pharmacopoeia (2020)	Inadequate
	Baical Skullcap Root	*Scutellaria baicalensis* Georgi	Huang Qin	Johann Gottlieb Georgi	Lamiaceae	Scutellaria	Root	Remove impurities, boil in water for 10 minutes, take out, stew to soften, cut into thin slices, and dry.	Baicalin, Baicalein, Wogonin, Wogonoside, Oroxylin A, Scutellarin	9	China Pharmacopoeia (2020)	Inadequate
**QQHT**	Snakegourd Seed	*Trichosanthes kirilowii* Maxim.	Gua Lou Ren	Karl Ivanovich Maximowicz	Cucurbitaceae	Trichosanthes	Seed	Wash, dry	Trichosanthes saponins (I-V), Linoleic acid, α-Linolenic acid, Oleic acid, Quercetin	12	China Pharmacopoeia (2020)	Inadequate

	Indian Bread	*Wolfiporia cocos* (F.A.Wolf) Ryvarden & Gilb.	Fu Ling	Frederick Adolph Wolf, Leif Ryvarden, Robert Lee Gilbertson	Polyporaceae	Wolfiporia	Sclerotium	Peel, cut, and dry	Poria cocos polysaccharides (pachyman, pachymaran), Pachymic acid, Dehydrotumulosic acid, Ergosterol	12	China Pharmacopoeia (2020)	Inadequate
	Arisaema Tuber	*Arisaema erubescens* (Wall.) Schott	Tian Nan Xing	Nathaniel Wallich, Heinrich Wilhelm Schott	Araceae	Arisaema	Tuber	Remove impurities, wash clean, and dry	Arisaemine, Arisaematine, Arisantuberine, Ursolic acid, Quercetin	6	China Pharmacopoeia (2020)	Inadequate
	Pinellia Tuber	*Pinellia ternata* (Thunb.) Breit.	Ban Xia	Carl Peter Thunberg, Karl (Max) Breiter	Araceae	Pinellia	Tuber	Crush before use	Pinellinine, L-(-)-N-methylguvacine, Tuberostemonine, p-Hydroxybenzoic acid, Methylparaben	9	China Pharmacopoeia (2020)	Inadequate
	Fresh Ginger	*Zingiber officinale* Roscoe	Sheng Jiang	William Roscoe	Zingiberaceae	Zingiber	Fresh Rhizome	Clean and slice	6-Gingerol, Zingiberene, Zingerone, Shogaol, Paradol	9	China Pharmacopoeia (2020)	Inadequate
	Common Reed Stem	*Phragmites australis* (Cav.) Trin. ex Steud.	Wei Jing	Antonio José Cavanilles, Carl Bernhard von Trinius, Ernst Gottlieb von Steudel	Poaceae	Phragmites	Stem	Clean, cut, and dry	Phragmites polysaccharides, Chlorogenic acid, Quercetin, Ursolic acid, Ferulic acid	30	China Pharmacopoeia (2020)	Inadequate
**WJ**	Coix Seed	*Coix lacryma-jobi* L. var. *ma-yuen* (Rom.Caill.) Stapf	Yi Yi Ren	Carl Linnaeus, Romain Caillieux, Otto Stapf	Poaceae	Coix	Seed	Remove shell, clean, and dry	Coixenolide, Linoleic acid, α-Linolenic acid, Oleic acid, Oleanolic acid	30	China Pharmacopoeia (2020)	Inadequate
	Peach Kernel	*Prunus persica* (L.) Batsch	Tao Ren	Carl Linnaeus	Rosaceae	Prunus	Dried ripe seed	Remove impurities and crush when in use	Amygdalin, Linoleic acid, Oleic acid, β-Sitosterol, Quercetin	9	China Pharmacopoeia (2020)	Inadequate
	Wax Gourd Seed	*Benincasa hispida* (Thunb.) Cogn.	Dong Gua Ren	Carl Peter Thunberg, Célestin Alfred Cogniaux	Cucurbitaceae	Benincasa	Seed	Wash, dry	Fatty acids (linoleic acid, oleic acid), Triterpenoids (oleanolic acid), Flavonoids (quercetin)	15	China Pharmacopoeia (2020)	Inadequate
	Gypsum	Gypsum Fibrosum	Shi Gao	Mineral (no botanical authority)	N/A	N/A	Mineral	Crush into powder	Calcium sulfate dihydrate ($CaSO_4 \cdot 2H_2O$), Trace elements (Mg, Al, Fe, K)	30	China Pharmacopoeia (2020)	Inadequate
**XBCQ**	Rhubarb	*Rheum palmatum* L.	Da Huang	Carl Linnaeus	Polygonaceae	Rheum	Dried root and rhizome	Remove impurities, wash thoroughly, moisten until softened, cut into thick slices or chunks, and dry	Anthraquinones (rhein, chrysophanol, emodin, aloe-emodin), Emodin-8-glucoside, Physcion	6	China Pharmacopoeia (2020)	Inadequate
	Bitter Apricot Seed	*Prunus armeniaca* L. *var. ansu* Maxim.	Ku Xing Ren	Carl Linnaeus, Karl Ivanovich Maximowicz	Rosaceae	Prunus	Seed	Remove impurities	Amygdalin, Amygdalin saponins, Quercetin, Oleic acid, Linoleic acid	9	China Pharmacopoeia (2020)	Inadequate
	Snakegourd Peel	*Trichosanthes kirilowii* Maxim.	Gua Lou Pi	Karl Ivanovich Maximowicz	Cucurbitaceae	Trichosanth es	Peel	Wash, cut, and dry	Trichosanthes saponins, Hesperidin, Naringin, D-Limonene, Quercetin	12	China Pharmacopoeia (2020)	Inadequate
	White Mulberry Root Bark	*Morus alba* L.	Sang Bai Pi	Carl Linnaeus	Moraceae	Morus	Root Bark	Remove coarse bark, wash clean and dry	Mulberroside A, Oxyresveratrol, Kuwanon G, Morin, 1-Deoxynojirimycin	15	China Pharmacopoeia (2020)	Inadequate
	Pinellia Tuber	*Pinellia ternata* (Thunb.) Breit.	Ban Xia	Carl Peter Thunberg, Karl (Max) Breiter	Araceae	Pinellia	Tuber	Crush before use	Pinellinine, L-(-)-N-methylguvacine, Tuberostemonine, p-Hydroxybenzoic acid, Methylparaben	9	China Pharmacopoeia (2020)	Inadequate
	Perilla Seed	*Perilla frutescens* (L.) Britt.	Zi Su Zi	Carl Linnaeus, Nathaniel Lord Britton	Lamiaceae	Perilla	Dried Seed	Remove impurities, wash clean, and dry	α-Linolenic acid, Perilla saponins (I-IV), Perilla ketone, Flavonoids (apigenin, luteolin)	9	China Pharmacopoeia (2020)	Inadequate
**SBP**	Bitter Apricot Seed	*Prunus armeniaca* L. var. *ansu* Maxim.	Ku Xing Ren	Carl Linnaeus, Karl Ivanovich Maximowicz	Rosaceae	Prunus	Seed	Remove impurities	Amygdalin, Amygdalin saponins, Quercetin, Oleic acid, Linoleic acid	9	China Pharmacopoeia (2020)	Inadequate

	Zhejiang Fritillary Bulb	*Fritillaria thunbergii* Miq.	Zhe Bei Mu	Carl (Karl) Miq. (Friedrich Anton Wilhelm Miquel)	Liliaceae	Fritillaria	Bulb	Remove soil, dry	Peimine, Peiminine, Fritillarine, Peimisine, Ebeiedine	12	China Pharmacopoeia (2020)	Inadequate
	Cape Jasmine Fruit	*Gardenia jasminoides* J.Ellis	Zhi Zi	John Ellis	Rubiaceae	Gardenia	Fruit	Clean and dry	Geniposide, Genipin, Crocin, Crocetin, Quercetin	9	China Pharmacopoeia (2020)	Inadequate
	Baical Skullcap Root	*Scutellaria baicalensis* Georgi	Huang Qin	Johann Gottlieb Georgi	Lamiaceae	Scutellaria	Root	Remove impurities, boil in water for 10 minutes, take out, stew to soften, cut into thin slices, and dry.	Baicalin, Baicalein, Wogonin, Wogonoside, Oroxylin A, Scutellarin	9	China Pharmacopoeia (2020)	Inadequate
	Chinese Goldthread	*Coptis chinensis* Franch.	Huang Lian	Adrien René Franchet	Ranunculaceae	Coptis	Rhizome	Clean, remove hair, slice, and dry	Berberine, Palmatine, Coptisine, Jatrorrhizine, Epiberberine	6	China Pharmacopoeia (2020)	Inadequate
	Perilla Seed	*Perilla frutescens* (L.) Britt.	Zi Su Zi	Carl Linnaeus, Nathaniel Lord Britton	Lamiaceae	Perilla	Dried Seed	Remove impurities, wash clean, and dry	α-Linolenic acid, Perilla saponins (I-IV), Perilla ketone, Flavonoids (apigenin, luteolin)	12	China Pharmacopoeia (2020)	Inadequate
	Pinellia Tuber	*Pinellia ternata* (Thunb.) Breit.	Ban Xia	Carl Peter Thunberg, Karl (Max) Breiter	Araceae	Pinellia	Tuber	Crush before use	Pinellinine, L-(-)-N-methylguvacine, Tuberostemonine, p-Hydroxybenzoic acid, Methylparaben	9	China Pharmacopoeia (2020)	Inadequate
	Hogfennel Root	*Peucedanum praeruptorum* (Dunn) Pimenov	Qian Hu	Stephen Troyte Dunn	Apiaceae	Peucedanum	Root	Clean, slice, and dry	Decursinol angelate, Decursin, Imperatorin, Bergapten, Psoralen	9	China Pharmacopoeia (2020)	Inadequate
	Magnolia Bark	*Magnolia officinalis* Rehder & E.H.Wilson	Hou Po	Alfred Rehder, Ernest Henry Wilson	Magnoliaceae	Magnolia	Bark	Clean, boil, curl, and dry	Honokiol, Magnolol, Oleanolic acid, Ursolic acid, Magnolignan	9	China Pharmacopoeia (2020)	Inadequate
**SZJQ**	Aged Tangerine Peel	*Citrus reticulata* Blanco 'Chachi'	Chen Pi	Francisco Manuel Blanco	Rutaceae	Citrus	Aged Peel	Clean, sun-dry, and store for aging	D-Limonene, Hesperidin, Neohesperidin, Scopoletin, Ursolic acid	9	China Pharmacopoeia (2020)	Inadequate
	Chinese Angelica	*Angelica sinensis* (Oliv.) Diels	Dang Gui	Daniel Oliver, Ludwig Diels	Apiaceae	Angelica	Root	Clean, soften, slice, and dry	Ligustilide, Butylphthalide, Ferulic acid, Z-ligustilide, Senkyunolide A	9	China Pharmacopoeia (2020)	Inadequate
	Licorice	*Glycyrrhiza uralensis* Fisch.	Gan Cao	Friedrich Ernst Ludwig von Fischer	Fabaceae	Glycyrrhiza	Dried root and rhizome	Remove impurities, wash clean, and dry	Glycyrrhizin, Glycyrrhetic acid, Liquiritin, Liquiritigenin, Isoliquiritin, Licoricidin, Glycyrol	6	China Pharmacopoeia (2020)	Inadequate
	Fresh Ginger	*Zingiber officinale* Roscoe	Sheng Jiang	William Roscoe	Zingiberaceae	Zingiber	Fresh Rhizome	Clean and slice	6-Gingerol, Zingiberene, Zingerone, Shogaol, Paradol	6	China Pharmacopoeia (2020)	Inadequate
	Jujube Fruit	*Ziziphus jujuba* Mill.	Da Zao	Philip Miller	Rhamnaceae	Ziziphus	Fruit	Clean and dry	Jujube polysaccharides, Hesperidin, Ursolic acid, Quercetin, Vitamin C	5 pieces	China Pharmacopoeia (2020)	Inadequate
	Cassia Bark	*Cinnamomum cassia* (L.) J.Presl	Rou Gui	Carl Linnaeus, Jan Svatopluk Presl	Lauraceae	Cinnamomum	Bark	Clean, remove cork layer, roll into quills, and dry	Cinnamaldehyde, Cinnamic acid, Eugenol, Coumarin, Cinnamyl alcohol	3	China Pharmacopoeia (2020)	Inadequate
	Pinellia Tuber	*Pinellia ternata* (Thunb.) Breit.	Ban Xia	Carl Peter Thunberg, Karl (Max) Breiter	Araceae	Pinellia	Tuber	Crush before use	Pinellinine, L-(-)-N-methylguvacine, Tuberostemonine, p-Hydroxybenzoic acid, Methylparaben	9	China Pharmacopoeia (2020)	Inadequate
	Red Tangerine Exocarp	*Citrus reticulata* Blanco 'Chachi'	Ju Hong	Francisco Manuel Blanco	Rutaceae	Citrus	Outer red pericarp of mature fruit	Clean, sun-dry	Hesperidin, Neohesperidin, D-Limonene, Quercetin, Ursolic acid	9	China Pharmacopoeia (2020)	Inadequate
	Indian Bread	*Wolfiporia cocos* (F.A.Wolf) Ryvarden & Gilb.	Fu Ling	Frederick Adolph Wolf, Leif Ryvarden, Robert Lee Gilbertson	Polyporaceae	Wolfiporia	Sclerotium	Peel, cut, and dry	Poria cocos polysaccharides (pachyman, pachymaran), Pachymic acid, Dehydrotumulosic acid, Ergosterol	15	China Pharmacopoeia (2020)	Inadequate

**ECSZYQ**	Licorice	*Glycyrrhiza uralensis* Fisch.	Gan Cao	Friedrich Ernst Ludwig von Fischer	Fabaceae	Glycyrrhiza	Dried root and rhizome	Remove impurities, wash clean, and dry	Glycyrrhizin, Glycyrrhetic acid, Liquiritin, Liquiritigenin, Isoliquiritin, Licoricidin, Glycyrol	6	China Pharmacopoeia (2020)	Inadequate
	Fresh Ginger	*Zingiber officinale* Roscoe	Sheng Jiang	William Roscoe	Zingiberaceae	Zingiber	Fresh Rhizome	Clean and slice	6-Gingerol, Zingiberene, Zingerone, Shogaol, Paradol	9	China Pharmacopoeia (2020)	Inadequate
	Smoked Plum	*Prunus mume* (Siebold & Zucc.) de Vriese	Wu Mei	Philipp Franz von Siebold, Joseph Gerhard Zuccarini	Rosaceae	Prunus	Smoked Fruit	Smoke until black then dry	5-Hydroxymethylfurfural, Citric acid, Malic acid, Quercetin, Rutin	1 piece	China Pharmacopoeia (2020)	Inadequate
	Perilla Seed	*Perilla frutescens* (L.) Britt.	Zi Su Zi	Carl Linnaeus, Nathaniel Lord Britton	Lamiaceae	Perilla	Dried Seed	Remove impurities, wash clean, and dry	α-Linolenic acid, Perilla saponins (I-IV), Perilla ketone, Flavonoids (apigenin, luteolin)	9	China Pharmacopoeia (2020)	Inadequate
	White Mustard Seed	*Sinapis alba* L.	Bai Jie Zi	Carl Linnaeus	Brassicaceae	Sinapis	Seed	Remove impurities, dry	Sinigrin, Allyl isothiocyanate, Myrosinase, Quercetin, Kaempferol	6	China Pharmacopoeia (2020)	Inadequate
	Radish Seed	*Raphanus sativus* L.	Lai Fu Zi	Carl Linnaeus	Brassicaceae	Raphanus	Seed	Remove impurities, wash clean, and dry. Crush before use	Sinapine, Glucoraphanin, Phenethyl isothiocyanate, Quercetin, Rutin	9	China Pharmacopoeia (2020)	Inadequate

Annotation: According to the relevant laws and regulations, the preparation method for traditional Chinese medicine (TCM) decoction is as follows: Remove impurities and thoroughly clean the substances. Place them into a clean container, add sufficient water, and soak for about 30 minutes until softened. Transfer the substances and soaking liquid to a ceramic or stainless steel pot, add 6–10 times the weight of water relative to the substances, bring to a boil, then simmer on low heat for 30 minutes. After the first decoction, filter the liquid and add water to the residue for a second decoction for 20–25 minutes. Mix the two decoctions thoroughly, then filter again through a fine sieve or gauze to clarify. While hot, transfer the decoction into a clean container, seal it, and store. Consume within 24 hours. For long-term storage, the decoction should be reboiled for sterilization and then refrigerated. It should be consumed warm in divided doses, with the dosage and frequency adjusted according to medical advice.

Appendix Table A.4: Standard processing methods of herbs

Formula name	Common English Name	Chinese Name	Primary Processing Method
DC	Ginkgo Seed	Bai Guo	Stir-fried
	Ephedra Herb	Ma Huang	Honey-fried
	Perilla Seed	Zi Su Zi	Stir-fried
	Licorice	Gan Cao	Honey-fried
	Coltsfoot Flower	Kuan Dong Hua	Raw
	Bitter Apricot Seed	Ku Xing Ren	Stir-fried or blanched
	White Mulberry Root Bark	Sang Bai Pi	Honey-fried
	Baical Skullcap Root	Huang Qin	Raw
	Pinellia Tuber	Ban Xia	Prepared with ginger and alum
MXSG	Ephedra Herb	Ma Huang	Raw
	Bitter Apricot Seed	Ku Xing Ren	Stir-fried or blanched
	Gypsum	Shi Gao	Raw
	Licorice	Gan Cao	Honey-fried
XQL	Ephedra Herb	Ma Huang	Raw
	Manchurian Wild Ginger	Xi Xin	Raw
	Cinnamon Twig	Gui Zhi	Raw
	White Peony Root	Bai Shao	Raw
	Licorice	Gan Cao	Honey-fried
	Dried Ginger	Gan Jiang	Raw
	Pinellia Tuber	Ban Xia	Prepared with ginger and alum
	Chinese Magnolia Vine Fruit	Wu Wei Zi	Raw
YBBX	Ephedra Herb	Ma Huang	Raw
	Gypsum	Shi Gao	Raw
	Fresh Ginger	Sheng Jiang	Raw
	Jujube Fruit	Da Zao	Raw
	Licorice	Gan Cao	Raw
	Pinellia Tuber	Ban Xia	Prepared with ginger and alum
QQHT	Aged Tangerine Peel	Chen Pi	Raw
	Bitter Apricot Seed	Ku Xing Ren	Stir-fried or blanched
	Immature Bitter Orange Fruit	Zhi Shi	Stir-fried
	Baical Skullcap Root	Huang Qin	Raw
	Snakegourd Seed	Gua Lou Ren	Raw
	Indian Bread	Fu Ling	Raw
	Arisaema Tuber	Tian Nan Xing	Bile Arisaema
	Pinellia Tuber	Ban Xia	Prepared with ginger and alum
	Fresh Ginger	Sheng Jiang	Raw
WJ	Common Reed Stem	Wei Jing	Raw
	Coix Seed	Yi Yi Ren	Raw
	Peach Kernel	Tao Ren	Raw
	Wax Gourd Seed	Dong Gua Ren	Raw
XBCQ	Gypsum	Shi Gao	Raw
	Rhubarb	Da Huang	Raw
	Bitter Apricot Seed	Ku Xing Ren	Stir-fried or blanched
	Snakegourd Peel	Gua Lou Pi	Raw
SBP	White Mulberry Root Bark	Sang Bai Pi	Raw
	Pinellia Tuber	Ban Xia	Prepared with ginger and alum
	Perilla Seed	Zi Su Zi	Stir-fried
	Bitter Apricot Seed	Ku Xing Ren	Stir-fried or blanched
	Zhejiang Fritillary Bulb	Zhe Bei Mu	Raw
	Cape Jasmine Fruit	Zhi Zi	Raw
	Baical Skullcap Root	Huang Qin	Raw
	Chinese Goldthread	Huang Lian	Raw
SZJQ	Perilla Seed	Zi Su Zi	Stir-fried
	Pinellia Tuber	Ban Xia	Prepared with ginger and alum
	Hogfennel Root	Qian Hu	Raw
	Magnolia Bark	Hou Po	Ginger-fried
	Aged Tangerine Peel	Chen Pi	Raw
	Chinese Angelica	Dang Gui	Raw
	Licorice	Gan Cao	Honey-fried
	Fresh Ginger	Sheng Jiang	Raw
	Jujube Fruit	Da Zao	Raw
	Cassia Bark	Rou Gui	Raw
ECSZYQ	Pinellia Tuber	Ban Xia	Prepared with ginger and alum
	Red Tangerine Exocarp	Ju Hong	Raw
	Indian Bread	Fu Ling	Raw
	Licorice	Gan Cao	Honey-fried
	Fresh Ginger	Sheng Jiang	Raw
	Smoked Plum	Wu Mei	Raw
	Perilla Seed	Zi Su Zi	Stir-fried
	White Mustard Seed	Bai Jie Zi	Stir-fried
	Radish Seed	Lai Fu Zi	Stir-fried

Appendix Table A.5 Taxonomically verified scientific nomenclature of all cited species

Chinese name	Standardized Nomenclature
Bai Guo	*Ginkgo biloba* L. [Ginkgoaceae; Ginkgo semen]
Ma Huang	*Ephedra sinica* Stapf [Ephedraceae; Ephedrae herba]
Ban Xia	*Pinellia ternata* (Thunb.) Breit. [Araceae; Pinelliae rhizoma]
Kuan Dong Hua	*Tussilago farfara* L. [Asteraceae; Farfarae flos]
Sang Bai Pi	*Morus alba* L. [Moraceae; Mori cortex]
Zi Su Zi	*Perilla frutescens* (L.) Britt. [Lamiaceae; Perillae fructus]
Huang Qin	*Scutellaria baicalensis* Georgi [Lamiaceae; Scutellariae radix]
Ku Xing Ren	*Prunus armeniaca* L. var. *ansu* Maxim. [Rosaceae; Armeniacae semen amarum]
Gan Cao	*Glycyrrhiza uralensis* Fisch. [Fabaceae; Glycyrrhizae radix et rhizoma]
Zi Wan	*Aster tataricus* L.f. [Asteraceae; Asteris radix et rhizoma]
Zhi Mu	*Anemarrhena asphodeloides* Bunge [Asparagaceae; Anemarrhenae rhizoma]
Zhi Shi	*Citrus × aurantium* L. [Rutaceae; Aurantii fructus immaturus]
Chen Pi	*Citrus reticulata* Blanco 'Chachi' [Rutaceae; Citri reticulatae pericarpium]
Zhe Bei Mu	*Fritillaria thunbergii* Miq. [Liliaceae; Fritillariae thunbergii bulbus]
Huang Qi	*Astragalus mongholicus* Bunge [Fabaceae; Astragali radix]
Tai Zi Shen	*Pseudostellaria heterophylla* (Miq.) Pax [Caryophyllaceae; Pseudostellariae radix]
Fu Ling	*Wolfiporia cocos* (F.A.Wolf) Ryvarden & Gilb. [Polyporaceae; Poria]
Jie Geng	*Platycodon grandiflorus* (Jacq.) A.DC. [Campanulaceae; Platycodonis radix]
Bai Bu	*Stemona japonica* (Blume) Miq. [Stemonaceae; Stemonae radix]
Bai Jie Zi	*Sinapis alba* L. [Brassicaceae; Sinapis semen]
Lai Fu Zi	*Raphanus sativus* L. [Brassicaceae; Raphani semen]
Wu Mei	*Prunus mume* (Siebold & Zucc.) de Vriese [Rosaceae; Mume fructus]
Sheng Jiang	*Zingiber officinale* Roscoe [Zingiberaceae; Zingiberis rhizoma recens]
Da Zao	*Ziziphus jujuba* Mill. [Rhamnaceae; Jujubae fructus]
Dan Shen	Salvia miltiorrhiza Bunge [Lamiaceae; Salviae miltiorrhizae radix et rhizoma]
Gui Zhi	*Cinnamomum cassia* (L.) J.Presl [Lauraceae; Cinnamomi ramulus]
Cang Zhu	*Atractylodes lancea* (Thunb.) DC. [Asteraceae; Atractylodis rhizoma]
Hou Po	*Magnolia officinalis* Rehder & E.H.Wilson [Magnoliaceae; Magnoliae officinalis cortex]
Wei Jing	*Phragmites australis* (Cav.) Trin. ex Steud. [Poaceae; Phragmitis rhizoma]
Yi Yi Ren	*Coix lacryma-jobi* L. var. *ma-yuen* (Rom.Caill.) Stapf [Poaceae; Coicis semen]
Tao Ren	*Prunus persica* (L.) Batsch [Rosaceae; Persicae semen]
Dong Gua Ren	*Benincasa hispida* (Thunb.) Cogn. [Cucurbitaceae; Benincasae semen]
Gua Lou Pi	*Trichosanthes kirilowii* Maxim. [Cucurbitaceae; Trichosanthis pericarpium]
Yu Xing Cao	*Houttuynia cordata* Thunb. [Saururaceae; Houttuyniae herba]
Ting Li Zi	*Descurainia sophia* (L.) Webb ex Prantl [Brassicaceae; Descurainiae semen]
Gua Lou	*Trichosanthes kirilowii* Maxim. [Cucurbitaceae; Trichosanthis fructus]
Qian Hu	*Peucedanum praeruptorum* (Dunn) Pimenov [Apiaceae; Peucedani radix]
Mai Dong	*Ophiopogon japonicus* (L.f.) Ker Gawl. [Asparagaceae; Ophiopogonis radix]
Bei Sha Shen	*Glehnia littoralis* (A.Gray) F.Schmidt [Apiaceae; Glehniae radix]
Huang Lian	*Coptis chinensis* Franch. [Ranunculaceae; Coptidis rhizoma]
Zhi Zi	*Gardenia jasminoides* J.Ellis [Rubiaceae; Gardeniae fructus]
Dang Gui	*Angelica sinensis* (Oliv.) Diels [Apiaceae; Angelicae sinensis radix]
Rou Gui	*Cinnamomum cassia* (L.) J.Presl [Lauraceae; Cinnamomi cortex]
Zi Su Ye	*Perilla frutescens* (L.) Britt. [Lamiaceae; Perillae folium]
Bai Zhu	*Atractylodes macrocephala* Koidz. [Asteraceae; Atractylodis macrocephalae rhizoma]
Zhi Qiao	*Citrus × aurantium* L. [Rutaceae; Aurantii fructus]
Chi Shao	*Paeonia lactiflora* Pall. [Paeoniaceae; Paeoniae radix rubra]
Chuan Xiong	*Conioselinum anthriscoides* 'Chuanxiong' [Apiaceae; Chuanxiong rhizoma]
Tian Nan Xing	Arisaema erubescens (Wall.) Schott [Araceae; Arisaematis Rhizoma]

Bai Tou Weng	*Pulsatilla chinensis* (Bunge) Regel [Ranunculaceae; Pulsatillae radix]
Pi Pa Ye	*Eriobotrya japonica* (Thunb.) Lindl. [Rosaceae; Eriobotryae folium]
Hong Jing Tian	*Rhodiola crenulata* (Hook.f. & Thomson) H.Ohba [Crassulaceae; Rhodiolae crenulatae radix et rhizoma]
Bai Qian	*Cynanchum stauntonii* (Decne.) Schltr. ex H.Lév. [Apocynaceae; Cynanchi stauntonii rhizoma et radix]
She Gan	*Belamcanda chinensis* (L.) Redouté [Iridaceae; Belamcandae rhizoma]
Jin Yin Hua	*Lonicera japonica* Thunb. [Caprifoliaceae; Lonicerae japonicae flos]
Xi Xin	*Asarum heterotropoides* F.Schmidt [Aristolochiaceae; Asari radix et rhizoma]
Gan Jiang	*Zingiber officinale* Roscoe [Zingiberaceae; Zingiberis rhizoma]
Wu Wei Zi	*Schisandra chinensis* (Turcz.) Baill. [Schisandraceae; Schisandrae chinensis fructus]
Bai Shao	*Paeonia lactiflora* Pall. [Paeoniaceae; Paeoniae radix alba]
Jing Jie	*Schizonepeta tenuifolia* (Benth.) Briq. [Lamiaceae; Schizonepetae herba]
Qiang Huo	*Notopterygium incisum* Ting ex H.T.Chang [Apiaceae; Notopterygii rhizoma et radix]
Xin Yi	*Magnolia biondii* Pamp. [Magnoliaceae; Magnoliae flos]
Du Huo	*Angelica biserrata* (R.H.Shan & C.Q.Yuan) C.Q.Yuan & R.H.Shan [Apiaceae; Angelicae biserrata radix]
Wu Yao	*Lindera aggregata* (Sims) Kosterm. [Lauraceae; Linderae radix]
Dang Shen	*Codonopsis pilosula* (Franch.) Nannf. [Campanulaceae; Codonopsis radix]
Bu Gu Zhi	*Psoralea corylifolia* L. [Fabaceae; Psoraleae fructus]
Lian Qiao	*Forsythia suspensa* (Thunb.) Vahl [Oleaceae; Forsythiae fructus]
Xie Bai	*Allium macrostemon* Bunge [Amaryllidaceae; Allii macrostemonis bulbus]

Appendix Table A.6–A.14: Search Strategy

Table A.6: Search Strategy: PubMed

#1	Pulmonary Disease, Chronic Obstructive[Mesh]
#2	(Pulmonary Disease, Chronic Obstructive[TIAB]) OR (Chronic Obstructive Pulmonary Diseases[TIAB]) OR (COPD[TIAB]) OR (Chronic Obstructive Lung Disease[TIAB]) OR (Chronic Obstructive Pulmonary Disease[TIAB]) OR (COAD[TIAB]) OR (Chronic Obstructive Airway Disease[TIAB]) OR (Airflow Obstruction, Chronic[TIAB]) OR (Airflow Obstructions, Chronic[TIAB]) OR (Chronic Airflow Obstructions[TIAB]) OR (Chronic Airflow Obstruction[TIAB])
#3	#1 OR #2
#4	Drugs, Chinese Herbal[Mesh]
#5	(Drugs, Chinese Herbal[TIAB]) OR (Chinese Drugs, Plant[TIAB]) OR (Chinese Herbal Drugs[TIAB]) OR (Herbal Drugs, Chinese[TIAB]) OR (Plant Extracts, Chinese[TIAB]) OR (Chinese Plant Extracts[TIAB]) OR (Extracts, Chinese Plant[TIAB])
#6	(Dingchuan Decoction[TIAB] OR Maxingshigan Decoction[TIAB] OR Xiaoqinglong Decoction[TIAB] OR Yuebibanxia Decoction[TIAB] OR Qingqihuatan Decoction[TIAB] OR Weijing Decoction[TIAB] OR Xuanbaichengqi Decoction[TIAB] OR Sangbaipi Decoction[TIAB] OR Suzijiangqi Decoction[TIAB] OR Erchen Decoction[TIAB] OR Sanziyangqin Decoction[TIAB])
#7	#4 OR #5 OR #6
#8	Randomized Controlled Trial [Publication Type]
#9	Randomized[TIAB] OR Trial[TIAB]
#10	#8 OR #9
	#3 AND #7 AND #10

Table A.7: Search Strategy: Embase

#1	'chronic obstructive lung disease'/exp
#2	'chronic airflow obstruction':ab,ti OR 'chronic airway obstruction':ab,ti OR 'chronic obstructive bronchopulmonary disease':ab,ti OR 'chronic obstructive lung disorder':ab,ti OR 'chronic obstructive pulmonary disease':ab,ti OR 'chronic obstructive pulmonary disorder':ab,ti OR 'chronic obstructive respiratory disease':ab,ti OR 'chronic pulmonary obstructive disease':ab,ti OR 'chronic pulmonary obstructive disorder':ab,ti OR 'copd':ab,ti OR 'lung chronic obstructive disease':ab,ti OR 'lung disease, chronic obstructive':ab,ti OR 'obstructive chronic lung disease':ab,ti OR 'obstructive chronic pulmonary disease':ab,ti OR 'obstructive lung disease, chronic':ab,ti OR 'pulmonary disease, chronic obstructive':ab,ti OR 'pulmonary disorder, chronic obstructive':ab,ti OR 'chronic obstructive lung disease':ab,ti
#3	#1 OR #2
#4	'Chinese medicine'/exp
#5	'chinese herbal medicine':ab,ti OR 'chinese traditional medicine':ab,ti OR 'medicine, chinese traditional':ab,ti OR 'traditional chinese medicine':ab,ti OR 'chinese medicine':ab,ti
#6	'dingchuan decoction':ab,ti OR 'maxingshigan decoction':ab,ti OR 'xiaoqinglong decoction':ab,ti OR 'yuebibanxia decoction':ab,ti OR 'qingqihuatan decoction':ab,ti OR 'weijing decoction':ab,ti OR 'sangbaipi decoction':ab,ti OR 'xuanbaichengqi decoction':ab,ti OR 'suzijiangqi decoction':ab,ti OR 'erchen decoction':ab,ti OR 'sanziyangqin decoction':ab,ti
#7	#4 OR #5 OR #6
#8	'randomized controlled trial'/exp OR 'controlled clinical trial'/exp
#9	'random':ab,ti OR 'controlled trial':ab,ti OR 'clinical trial':ab,ti OR rct:ab,ti
#10	#8 OR #9
#11	#3 AND #7 AND #10

Table A.8: Search Strategy: Cochrane Central Register of Controlled Trials (CENTRAL)

#1	(chronic obstructive pulmonary disease OR COPD OR AECOPD OR chronic obstructive lung disease OR COAD OR chronic airflow obstruction):ti,ab,kw
#2	(Chinese herbal medicine OR traditional Chinese medicine OR Chinese medicine OR Chinese drug OR Chinese plant extract):ti,ab,kw
#3	(Dingchuan decoction OR Maxing Shigan decoction OR Xiao Qinglong decoction OR Yuebibanxia decoction OR Qingqi Huatan decoction OR Weijing decoction):ti,ab,kw
#4	(Xuanbai Chengqi decoction OR Sangbaipi decoction OR Suzi Jiangqi decoction OR Erchen decoction OR Sanzi Yangqin decoction):ti,ab,kw
#5	#2 OR #3 OR #4
#6	#1 AND #5

Table A.9: Search Strategy: Cumulative Index to Nursing and Allied Health Literature (CINAHL)

#1	(MH "Pulmonary Disease, Chronic Obstructive+")
#2	TI (("Pulmonary Disease, Chronic Obstructive") OR ("Chronic Obstructive Pulmonary Diseases") OR (COPD) OR ("Chronic Obstructive Lung Disease") OR ("Chronic Obstructive Pulmonary Disease") OR (COAD) OR ("Chronic Obstructive Airway Disease") OR ("Chronic Airflow Obstruction")) OR AB (("Pulmonary Disease, Chronic Obstructive") OR ("Chronic Obstructive Pulmonary Diseases") OR (COPD) OR ("Chronic Obstructive Lung Disease") OR ("Chronic Obstructive Pulmonary Disease") OR (COAD) OR ("Chronic Obstructive Airway Disease") OR ("Chronic Airflow Obstruction"))
#3	#1 OR #2
#4	(MH "Chinese Herbal Medicine+") OR (MH "Chinese Traditional Medicine+") OR (MH "Herbal Medicine+")
#5	TI (("Drugs, Chinese Herbal") OR ("Chinese Herbal Drugs") OR ("Chinese Plant Extracts") OR ("Traditional Chinese Medicine") OR ("Chinese Medicine")) OR AB (("Drugs, Chinese Herbal") OR ("Chinese Herbal Drugs") OR ("Chinese Plant Extracts") OR ("Traditional Chinese Medicine") OR ("Chinese Medicine"))
#6	TI (("Dingchuan Decoction") OR ("Maxing Shigan Decoction") OR ("Xiao Qinglong Decoction") OR ("Yuebibanxia Decoction") OR ("Qingqi Huatan Decoction") OR ("Weijing Decoction") OR ("Xuanbai Chengqi Decoction") OR ("Sangbaipi Decoction") OR ("Suzi Jiangqi Decoction") OR ("Erchen Decoction") OR ("Sanzi Yangqin Decoction")) OR AB (("Dingchuan Decoction") OR ("Maxing Shigan Decoction") OR ("Xiao Qinglong Decoction") OR ("Qingqi Huatan Decoction") OR ("Weijing Decoction") OR ("Xuanbai Chengqi Decoction") OR ("Sangbaipi Decoction") OR ("Suzi Jiangqi Decoction") OR ("Erchen Decoction") OR ("Sanzi Yangqin Decoction"))
#7	#4 OR #5 OR #6
#8	(MH "Clinical Trials+") OR (PT "clinical trial") OR (PT "randomized controlled trial")
#9	#3 AND #7 AND #8

Table A.10: Search Strategy: Web of Science

#1	TS=("pulmonary disease, chronic obstructive" OR "chronic obstructive pulmonary diseases" OR COPD OR "chronic obstructive lung disease" OR "chronic obstructive pulmonary disease" OR COAD OR "chronic obstructive airway disease" OR "chronic airflow obstruction")
#2	TS=("drugs, chinese herbal" OR "chinese herbal drugs" OR "chinese plant extracts" OR "traditional chinese medicine" OR "chinese medicine" OR "chinese herbal medicine")
#3	TS=("dingchuan decoction" OR "maxingshigan decoction" OR "xiaoqinglong decoction" OR "yuebibanxia decoction" OR "qingqihuatan decoction" OR "weijing decoction" OR "xuanbaichengqi decoction" OR "sangbaipi decoction" OR "suzijiangqi decoction" OR "erchen decoction" OR "sanziyangqin decoction")
#4	#2 OR #3
#5	TS=(random* OR "controlled trial" OR "clinical trial" OR "randomized controlled trial" OR RCT)
#6	#1 AND #4 AND #5

Table A.11: Search Strategy: Chinese Biomedical Database (CBM)

#1	"肺疾病，慢性阻塞性"[常用字段:智能]
#2	"慢性阻塞肺疾病"[常用字段:智能] OR "COAD"[常用字段:智能] OR "慢性气道阻塞性疾病"[常用字段:智能] OR "慢性阻塞性肺疾病"[常用字段:智能] OR "慢性气道阻塞"[常用字段:智能] OR "慢性气流阻塞"[常用字段:智能] OR "气道阻塞, 慢性"[常用字段:智能] OR "COPD"[常用字段:智能] OR "慢性阻塞性肺疾病急性加重期"[常用字段:智能] OR "AECOPD"[常用字段:智能]
#3	#1 OR #2
#4	"定喘汤"[常用字段:智能] OR "麻杏石甘汤"[常用字段:智能] OR "小青龙汤"[常用字段:智能] OR "越婢半夏汤"[常用字段:智能] OR "清气化痰汤"[常用字段:智能] OR "苇茎汤"[常用字段:智能] OR "宣白承气汤"[常用字段:智能] OR "桑白皮汤"[常用字段:智能] OR "苏子降气汤"[常用字段:智能] OR "二陈汤合三子养亲汤"[常用字段:智能]
#5	"随机"[常用字段:智能] OR "随机对照"[常用字段:智能] OR "RCT"[常用字段:智能]
#6	#3 AND #4 AND #5

Table A.12: Search Strategy: China National Knowledge Infrastructure (CNKI)

#1	(慢性阻塞性肺疾病 + COPD + 慢性阻塞性肺疾病急性加重期 + AECOPD)[主题]
#2	(定喘汤 + 麻杏石甘汤 + 小青龙汤 + 越婢半夏汤 + 清气化痰汤 + 苇茎汤 + 宣白承气汤 + 桑白皮汤 + 苏子降气汤 + 二陈汤合三子养亲汤)[主题]
#3	(随机 + 随机对照 + RCT) [主题]
#4	#1 AND #2 AND #3

Table A.13: Search Strategy: Wanfang Data Knowledge Service Platform (Wanfang Database)

#1	**主题:**("肺疾病，慢性阻塞性")
#2	**主题:**("慢性阻塞肺疾病" OR "COAD" OR "慢性气道阻塞性疾病" OR "慢性阻塞性肺疾病" OR "慢性气道阻塞" OR "慢性气流阻塞" OR "气道阻塞，慢性" OR "COPD" OR "慢性阻塞性肺疾病急性加重期" OR "AECOPD")
#3	#1 OR #2
#4	**主题:**("定喘汤" OR "麻杏石甘汤" OR "小青龙汤" OR "越婢半夏汤" OR "清气化痰汤" OR "苇茎汤" OR "宣白承气汤" OR "桑白皮汤" OR "苏子降气汤" OR "二陈汤合三子养亲汤")
#5	**主题:**("随机" OR "随机对照" OR "RCT")
#6	#3 AND #4 AND #5

Table A.14: Search Strategy: Chongqing VIP Information Chinese Science and Technology Journal Database (VIP Database)

#1	**题名或关键词:**("肺疾病，慢性阻塞性")
#2	题名或关键词:("慢性阻塞肺疾病" OR "COAD" OR "慢性气道阻塞性疾病" OR "慢性阻塞性肺疾病" OR "慢性气道阻塞" OR "慢性气流阻塞" OR "气道阻塞，慢性" OR "COPD" OR "慢性阻塞性肺疾病急性加重期" OR "AECOPD")
#3	#1 OR #2
#4	题名或关键词:("定喘汤" OR "麻杏石甘汤" OR "小青龙汤" OR "越婢半夏汤" OR "清气化痰汤" OR "苇茎汤" OR "宣白承气汤" OR "桑白皮汤" OR "苏子降气汤" OR "二陈汤合三子养亲汤")
#5	**题名或关键词:**("随机" OR "随机对照" OR "RCT")
#6	#3 AND #4 AND #5

Appendix Table A.15: Characteristics of included studies

Study ID	Age (year) (Mean ± SD)	Gender (Men/Women)	Severity[a]	Duration of condition (year) (Mean ± SD)	Sample size (randomized/assessed)	Intervention	Control	Treatment duration	Outcomes	Adverse events	Follow-up time
Lin F 2024	I: 63.18 ± 2.74; C: 62.39 ± 2.69	I: 28/16; C: 26/18	NR	I: 9.19 ± 1.27; C: 9.10 ± 1.25	I: 44/44; C: 44/44	DC + ④	④	2w	ER; FEV_1; CAT	NR	NR
Yao F 2021	I: 62.12 ± 1.03; C: 62.54 ± 1.87	I: 28/12; C: 29/11	NR	I: 6.58 ± 1.65; C: 7.03 ± 1.65	I: 40/40; C: 40/40	DC + ③	③	15d	FEV_1; FEV_1/FVC	NR	NR
Zeng YZ 2024	I: 66.89 ± 5.24; C: 67.52 ± 4.37	I: 19/17; C: 20/16	NR	I: 6.38 ± 2.16; C: 6.40 ± 2.07	I: 36/36; C: 36/36	DC + ②	②	2w	ER; FEV_1; PaO_2; $PaCO_2$	NR	NR
You WH 2022	I: 65.58 ± 10.11; C: 66.22 ± 10.05	I: 24/24; C: 23/25	NR	I: 8.28 ± 1.10; C: 7.85 ± 1.05	I: 48/48; C: 48/48	DC + ④	④	8w	ER; FEV_1%pred; CAT	NR	NR
Wu M 2021	48.6 ± 10.4	61/41	NR	NR	I: 51/51; C: 51/51	DC + ③	③	2w	ER; FEV_1; FEV_1/FVC	NR	NR
Guan ZY 2021	I: 62.40 ± 7.25; C: 62.37 ± 7.22	I: 46/27; C: 47/26	NR	NR	I: 73/73; C: 73/73	ECSZYQ + ③	③	2w	ER; FEV_1; FEV_1/FVC; PaO_2; $PaCO_2$	NR	NR
Xu H 2019	I: 47.53 ± 2.96; C: 47.50 ± 3.16	I: 34/22; C: 36/20	1-3	I: 6.44 ± 1.51; C: 6.49 ± 1.34	I: 56/56; C: 56/56	ECSZYQ + ④	④	1w	ER; FEV_1%pred; FEV_1/FVC	NR	NR
Wei J 2022	I: 59.38 ± 5.71; C: 59.42 ± 5.83	I: 20/22; C: 19/23	NR	I: 8.18 ± 2.41; C: 8.24 ± 2.55	I: 42/42; C: 42/42	ECSZYQ + ③	③	2w	ER; FEV_1	NR	NR
Yang XL 2014	I: 54.6; C: 52.8	I: 24/22; C: 22/18	NR	NR	I: 46/46; C: 40/40	ECSZYQ + ③	③	14d	ER; FEV_1%pred; FEV_1/FVC; PaO_2; $PaCO_2$	NR	NR
Wei JQ 2023	I: 66.10 ± 3.35; C: 66.10 ± 3.31	I: 22/18; C: 21/19	NR	I: 4.39 ± 1.50; C: 4.39 ± 1.51	I: 40/40; C: 40/40	ECSZYQ + ③	③	2w	ER; FEV_1%pred; FEV_1/FVC; CCQ	NR	1y
Feng J 2023	I: 63.51 ± 3.06; C: 62.98 ± 3.09	I: 18/17; C: 19/16	NR	NR	I: 35/35; C: 35/35	ECSZYQ + ④	④	12d	ER; FEV_1; FEV_1/FVC	I: 1 C: 8	NR
Qi YX 2016	I: 59.02 ± 10.82; C: 58.06 ± 11.23	I: 36/34; C: 38/32	NR	I: 10.02 ± 5.42; C: 9.98 ± 6.02	I: 70/70; C: 70/70	ECSZYQ + ④	④	12d	ER; FEV_1%pred; FEV_1/FVC	NR	NR
Yan LW 2020	I: 57.58 ± 6.17; C: 57.46 ± 5.78	I: 85/65; C: 87/63	NR	I: 6.63 ± 2.17; C: 6.85 ± 2.46	I: 150/150; C: 150/150	ECSZYQ + ③	③	10d	FEV_1	NR	NR
Fan PC 2020	I: 65.19 ± 3.74; C: 66.18 ± 3.37	I: 15/22; C: 17/20	NR	I: 7.84 ± 1.57; C: 7.24 ± 1.93	I: 37/37; C: 37/37	ECSZYQ + ④	④	12d	ER; FEV_1%pred; FEV_1/FVC	NR	NR
Wang XB 2020	I: 58.28 ± 3.42; C: 59.39 ± 3.29	I: 30/13; C: 29/14	NR	NR	I: 43/43; C: 43/43	ECSZYQ + ③	③	10d	ER; FEV_1; FEV_1/FVC	NR	NR
Bai YL 2018	I: 61.7 ± 3.9; C: 59.5 ± 4.3	I: 52/33; C: 47/38	NR	I: 11.3 ± 1.3; C: 10.7 ± 1.2	I: 85/85; C: 85/85	WJ + ③	③	2w	ER; FEV_1; FEV_1/FVC	NR	NR
Wang HX 2023	I: 69.74 ± 8.35; C: 70.36 ± 8.81	I: 24/18; C: 26/16	NR	I: 6.52 ± 2.76; C: 6.96 ± 3.03	I: 42/42; C: 42/42	XQL + ④	④	14d	ER; FEV_1%pred; FEV_1/FVC; PaO_2; $PaCO_2$	NR	NR
Xing HZ 2020	I: 69.5 ± 0.3; C: 68.7 ± 0.5	I: 22/16; C: 20/18	NR	NR	I: 38/38; C: 38/38	ECSZYQ + ③	③	2w	ER; FEV_1%pred; FEV_1/FVC	I: 3 C: 2	NR
Ma JQ 2022	I: 70.18 ± 2.79; C: 70.22 ± 2.82	I: 42/23; C: 43/21	1-2	I: 3.52 ± 0.38; C: 3.41 ± 0.35	I: 65/65; C: 64/64	XQL + ③	③	12w	ER; FEV_1; FEV_1%pred; FEV_1/FVC	NR	NR
Wu JN 2016	I: 71.1 ± 4.3; C: 70.2 ± 4.6	I: 40/20; C: 36/20	NR	I: 12.4 ± 5.5; C: 12.6 ± 5.7	I: 60/60; C: 56/56	DC + ②	②	2w	ER; FEV_1%pred; FEV_1/FVC; PaO_2; $PaCO_2$	I: 1 C: 1	6m
Yan HF 2020	I: 56.82 ± 5.83; C: 56.29 ± 5.63	I: 29/16; C: 29/16	1-3	I: 3.06 ± 0.41; C: 2.86 ± 0.46	I: 45/45; C: 45/45	DC + ④	④	2w	ER; FEV_1; FEV_1/FVC	I: 6 C: 8	NR
Wang W 2021	I: 41.71 ± 5.58; C: 41.87 ± 5.28	I: 22/27; C: 20/28	NR	I: 3.53 ± 0.97; C: 3.36 ± 0.91	I: 49/49; C: 48/48	MXSG + ③	③	14d	ER; FEV_1; FEV_1/FVC; PaO_2; $PaCO_2$	I: 5 C: 4	NR
Xu XP 2022	I: 63.11 ± 12.95; C: 62.45 ± 13.12	I: 32/23; C: 31/24	NR	I: 9.50 ± 1.14; C: 9.00 ± 1.12	I: 55/55; C: 55/55	MXSG + ③	③	2w	ER; FEV_1; FEV_1/FVC; PaO_2; $PaCO_2$	NR	NR
Zou TE 2021	I: 66.65 ± 7.41; C: 66.79 ± 7.46	NR	NR	NR	I: 60/60; C: 60/60	MXSG + ④	④	14d	ER; FEV_1%pred; FEV_1/FVC; CAT	NR	NR

Yue YX 2020	I: 60.8 ± 4.1; C: 61.4 ± 3.8	I: 31/16; C: 28/19	NR	I: 12.2 ± 1.4; C: 11.9 ± 1.6	I: 47/47; C: 47/47	WJ + ⑤	⑤	14d	ER; FEV_1; FEV_1/FVC	NR	NR
Mei Y 2022	I: 62.53 ± 2.19; C: 62.59 ± 2.23	I: 24/18; C: 23/19	1-3	I: 6.65 ± 1.18; C: 6.69 ± 1.45	I: 42/42; C: 42/42	WJ + ④	④	4w	ER; FEV_1	I: 2 C: 4	NR
Niu J 2023	I: 55.67 ± 13.27; C: 56.25 ± 12.10	I: 34/14; C: 30/17	1	I: 6.56 ± 2.13; C: 6.47 ± 2.51	I: 48/48; C: 47/47	SBP + ③	③	2w	ER; FEV_1; FEV_1%pred; FEV_1/FVC	I: 5 C: 3	3m
Bai FR 2024	I: 49.99 ± 6.01; C: 49.39 ± 5.26	I: 22/22; C: 21/23	NR	NR	I: 44/44; C: 44/44	SZJQ + ①	①	14d	ER; FEV_1%pred; FEV_1/FVC	I: 4 C: 2	NR
Jiang LX 2021	I: 63.7 ± 11.8; C: 62.9 ± 12.3	I: 24/19; C: 22/21	NR	I: 6.4 ± 1.7; C: 7.1 ± 2.3	I: 43/43; C: 43/43	SZJQ + ④	④	2w	ER; FEV_1; FEV_1%pred; FEV_1/FVC	NR	NR
Su ZX 2019	I: 69.42 ± 5.17; C: 70.83 ± 3.96	I: 20/15; C: 21/14	NR	I: 13.02 ± 2.79; C: 13.81 ± 3.57	I: 35/35; C: 35/35	SZJQ + ④	④	2w	FEV_1; FEV_1/FVC; SGRQ	NR	NR
Feng J 2018	I: 69.80 ± 7.50; C: 69.75 ± 7.55	I: 33/17; C: 32/18	NR	I: 10.50 ± 3.50; C: 10.45 ± 3.65	I: 50/50; C: 50/50	WJ + ⑤	⑤	14d	ER; FEV_1; FEV_1/FVC	NR	NR
Pan XD 2018	I: 63.81 ± 8.5; C: 63.27 ± 8.4	NR	NR	NR	I: 50/50; C: 50/50	XQL + ③	③	14d	FEV_1; FEV_1/FVC	NR	NR
Chen WS 2024	I: 67.56 ± 6.32; C: 67.61 ± 6.21	I: 31/29; C: 32/28	2-4	I: 7.32 ± 1.21; C: 7.21 ± 1.31	I: 60/60; C: 60/60	XBCQ + ④	④	9w	ER; FEV_1; FEV_1/FVC	NR	NR
Huang F 2012	63.4 ± 8.2	89/75	2	NR	I: 82/82; C: 82/82	MXSG + ③	③	14d	FEV_1%pred; FEV_1/FVC	NR	NR
Lan H 2019	I: 58.3 ± 4.2; C: 58.0 ± 4.6	I: 26/14; C: 25/14	NR	I: 8.3 ± 1.5; C: 8.2 ± 1.5	I: 40/40; C: 39/39	MXSG + ④	④	14d	ER; FEV_1	NR	NR
Wang ZF 2017	I: 62.2 ± 2.2; C: 62.7 ± 4.3	I: 24/16; C: 19/21	NR	I: 12.8 ± 2.8; C: 12.3 ± 2.1	I: 40/40; C: 40/40	MXSG + ④	④	14d	ER; FEV_1%pred; FEV_1/FVC	NR	NR
Xiong DN 2016	I: 58.5 ± 13.4; C: 57.8 ± 12.3	I: 29/16; C: 26/15	1-4	I: 7.1 ± 1.4; C: 6.7 ± 1.2	I: 45/45; C: 41/41	MXSG + ③	③	14d	ER; FEV_1%pred; FEV_1/FVC	NR	NR
Sun HJ 2017	I: 64.1 ± 7.3; C: 63.4 ± 7.2	I: 32/18; C: 36/14	2-3	I: 14.2 ± 5.7; C: 14.5 ± 5.8	I: 50/50; C: 50/50	MXSG + ④	④	2w	ER; FEV_1%pred; FEV_1/FVC	0	NR
Gong Y 2022	I: 58.12 ± 3.87; C: 58.07 ± 3.98	I: 23/17; C: 25/15	NR	I: 4.29 ± 0.54; C: 4.47 ± 0.57	I: 40/40; C: 40/40	MXSG + ②	②	2w	ER; FEV_1; FEV_1/FVC	0	NR
He CN 2017	I: 52.34 ± 12.13; C: 52.19 ± 12.45	I: 23/22; C: 24/21	NR	NR	I: 45/45; C: 45/45	MXSG + ⑤	⑤	14d	ER; FEV_1; FEV_1/FVC	NR	NR
Gao ZT 2022	I: 66.52 ± 3.15; C: 63.25 ± 3.24	I: 30/19; C: 29/20	NR	NR	I: 49/49; C: 49/49	MXSG + ④	④	7d	ER; FEV_1; FEV_1/FVC	I: 3 C: 10	NR
Lin CB 2018	I: 59.97 ± 5.99; C: 60.67 ± 6.79	I: 28/10; C: 24/14	NR	NR	I: 38/38; C: 38/38	MXSG + ⑤	⑤	14d	ER; FEV_1%pred; FEV_1/FVC;	NR	NR
Sun XS 2015	I: 63.2 ± 9.7; C: 61.9 ± 9.1	I: 79/27; C: 74/32	NR	I: 10.3 ± 4.9; C: 9.7 ± 4.6	I: 106/106; C: 106/106	MXSG + ⑤	⑤	14d	ER; FEV_1%pred; FEV_1/FVC;	NR	NR
Duan WY 2021	I: 58.87 ± 5.99; C: 58.76 ± 5.98	I: 27/19; C: 26/20	NR	I: 9.5 ± 4.3; C: 9.8 ± 4.5	I: 46/46; C: 46/46	MXSG + ④	④	14d	ER; FEV_1; FEV_1%pred; FEV_1/FVC	NR	NR
Liao Q 2020	I: 61.4 ± 4.5; C: 62.4 ± 4.4	I: 57/43; C: 56/44	NR	NR	I: 100/100; C: 100/100	MXSG + ③	③	2w	ER; FEV_1%pred; FEV_1/FVC;	0	NR
Xie WH 2009	I: 64.8; C: 65.1	I: 28/14; C: 26/14	1-3	I: 12.1; C: 11.8	I: 42/42; C: 40/40	MXSG + ③	③	15d	ER; FEV_1; FEV_1/FVC	NR	NR
Yan SL 2019	65.55 ± 6.21	47/33	1-3	6.15 ± 1.34	I: 40/40; C: 40/40	MXSG + ④	④	2w	ER; FEV_1; FEV_1/FVC	0	NR
Song ZC 2020	I: 60.31 ± 2.47; C: 61.22 ± 2.39	I: 20/16; C: 19/17	1-3	I: 6.84 ± 1.65; C: 6.74 ± 1.69	I: 36/36; C: 36/36	MXSG + ③	③	2w	ER; FEV_1; FEV_1/FVC; PaO_2; $PaCO_2$	NR	NR
Wang XP 2015	I: 58.32 ± 15.21; C: 52.64 ± 15.74	I: 20/19; C: 21/18	NR	NR	I: 39/39; C: 39/39	MXSG + ③	③	2w	ER; FEV_1; FEV_1/FVC	NR	NR
Wang XD 2017	I: 57.15 ± 10.75; C: 56.35 ± 11.45	I: 24/51; C: 23/52	NR	I: 3.76 ± 1.93; C: 3.81 ± 2.10	I: 75/75; C: 75/75	MXSG + ④	④	2w	ER; FEV_1	NR	NR

Liu YF 2023	I: 60.55 ± 6.52; C: 60.35 ± 6.48	I: 26/17; C: 23/20	NR	I: 8.71 ± 4.85; C: 8.69 ± 4.67	I: 43/43; C: 43/43	ECSZYQ + ③	③	14d	ER; FEV_1%pred; FEV_1/FVC;	NR	NR
Zhang Y 2023	I: 66.54 ± 7.69; C: 66.85 ± 7.85	I: 30/10; C: 27/13	NR	I: 4.88 ± 1.90; C: 4.71 ± 1.95	I: 40/40; C: 40/40	ECSZYQ + ④	④	2w	ER; FEV_1; FEV_1/FVC	NR	NR
Hao SR 2018	I: 69.05 ± 4.65; C: 68.85 ± 5.05	I: 40/20; C: 41/19	NR	I: 11.8 ± 1.3; C: 11.5 ± 1.5	I: 60/60; C: 60/60	WJ + ③	③	15d	ER; FEV_1%pred; FEV_1/FVC	I: 4 C: 7	NR
Dai YM 2013	I: 64.34 ± 3.85; C: 65.46 ± 2.96	I: 27/13; C: 28/12	NR	I: 1.44 ± 0.60; C: 1.53 ± 0.54	I: 40/40; C: 40/40	WJ + ③	③	20d	ER; FEV_1; FEV_1%pred	NR	NR
Ma ZH 2020	I: 64.36 ± 5.82; C: 64.27 ± 5.73	I: 36/15; C: 35/16	NR	NR	I: 51/51; C: 51/51	QQHT + ⑤	⑤	2w	ER; FEV_1	0	NR
Yao B 2023	I: 52.86 ± 4.20; C: 52.84 ± 4.19	I: 24/23; C: 25/22	NR	I: 4.87 ± 1.10; C: 5.03 ± 1.12	I: 47/47; C: 47/47	QQHT + ③	③	2w	ER; FEV_1%pred; FEV_1/FVC; PaO_2; $PaCO_2$	0	NR
Xie GB 2023	I: 56.92 ± 5.13; C: 57.24 ± 4.38	I: 80/78; C: 82/76	NR	I: 11.22 ± 3.65; C: 11.34 ± 2.79	I: 158/158; C: 158/158	QQHT + ④	④	14d	ER; FEV_1%pred; FEV_1/FVC	NR	NR
Zhao DL 2020	I: 55.29 ± 11.19; C: 47.56 ± 11.26	I: 29/31; C: 32/28	2-4	I: 3.92 ± 1.44; C: 4.53 ± 1.26	I: 60/60; C: 60/60	QQHT + ④	④	2w	ER; FEV_1%pred; FEV_1/FVC; PaO_2; $PaCO_2$	NR	NR
Kou ZJ 2024	I: 65.64 ± 6.46; C: 65.33 ± 6.54	I: 43/17; C: 45/15	NR	I: 7.71 ± 2.35; C: 7.56 ± 2.42	I: 60/60; C: 60/60	QQHT + ③	③	14d	ER; FEV_1; PaO_2; $PaCO_2$	I: 4 C: 5	NR
Su W 2020	I: 64.18 ± 3.49; C: 64.12 ± 3.52	I: 27/18; C: 25/20	NR	NR	I: 45/45; C: 45/45	QQHT + ③	③	14d	ER; FEV_1; FEV_1/FVC	NR	NR
Lv T 2014	I: 57.7 ± 2.6; C: 57.4 ± 2.3	I: 48/66; C: 51/53	NR	I: 10.5 ± 2.8; C: 10.2 ± 2.4	I: 104/104; C: 104/104	QQHT + ④	④	14d	ER; FEV_1; FEV_1%pred	NR	NR
Wang XH 2023	I: 61.37 ± 3.50; C: 61.35 ± 3.61	I: 22/16; C: 21/17	NR	I: 6.57 ± 2.51; C: 6.59 ± 2.68	I: 38/38; C: 38/38	QQHT + ③	③	2w	ER; FEV_1; FEV_1/FVC; PaO_2; $PaCO_2$	I: 3 C: 2	NR
Qian JY 2019	I: 57.0 ± 6.6; C: 56.9 ± 6.7	I: 35/25; C: 34/26	2-3	NR	I: 60/60; C: 60/60	ECSZYQ + ④	④	14d	ER; FEV_1; FEV_1/FVC	NR	NR
Yu XD 2022	I: 49.95 ± 4.34; C: 49.61 ± 4.57	I: 28/18; C: 27/19	NR	NR	I: 46/46; C: 46/46	ECSZYQ + ④	④	2w	ER; FEV_1; FEV_1/FVC; CAT	NR	NR
Tang XJ 2023	I: 66.12 ± 7.05 C: 65.74 ± 7.31	I: 27/16; C: 28/15	NR	I: 4.25 ± 2.02; C: 3.84 ± 1.75	I: 43/43; C: 43/43	SBP + ④	④	14d	ER; FEV_1; FEV_1/FVC	NR	NR
Liu GJ 2024	I: 55.78 ± 6.44; C: 54.78 ± 6.12	I: 20/22; C: 23/18	NR	I: 8.56 ± 2.32; C: 8.27 ± 2.54	I: 42/42; C: 41/41	SBP + ③	③	1w	ER; FEV_1; FEV_1/FVC	NR	NR
Liu YC 2022	I: 67.86 ± 3.26; C: 68.01 ± 3.13	I: 27/16; C: 24/19	NR	NR	I: 43/43; C: 43/43	SBP + ③	③	14d	ER; FEV_1; FEV_1/FVC	I: 2 C: 4	NR
Gao BJ 2022	I: 70.58 ± 4.69; C: 70.59 ± 4.68	I: 22/18; C: 21/19	NR	NR	I: 40/40; C: 40/40	SBP + ②	②	10d	FEV_1; FEV_1/FVC; PaO_2; $PaCO_2$; SGRQ	NR	NR
Liang ZX 2023	I: 66.21 ± 2.35; C: 69.31 ± 2.24	I: 60/40; C: 58/42	NR	NR	I: 100/100; C: 100/100	SBP + ④	④	10d	ER; FEV_1; FEV_1/FVC	NR	NR
Chen L 2018	I: 65.6 ± 6.8; C: 65.1 ± 6.5	I: 25/15; C: 24/16	2	I: 7.7 ± 1.2; C: 7.5 ± 1.3	I: 40/40; C: 40/40	SBP + ③	③	1w	ER; FEV_1; FEV_1%pred; PaO_2; $PaCO_2$	NR	NR
Liu JJ 2021	I: 65.20 ± 2.42; C: 65.14 ± 2.37	I: 25/15; C: 23/17	APACHE-II: T: 26.37 ± 3.05; C: 26.28 ± 3.11	I: 5.42 ± 0.68; C: 5.36 ± 0.72	I: 40/40; C: 40/40	SBP + ④	④	14d	ER; FEV_1; FEV_1/FVC; PaO_2; $PaCO_2$	I: 3 C: 7	NR
Fei X 2024	I: 67.31 ± 4.21; C: 65.92 ± 5.81	I: 49/26; C: 51/24	APACHE-II: T: 25.39 ± 3.92; C: 26.28 ± 4.18	I: 5.92 ± 2.81; C: 5.28 ± 1.92	I: 75/75; C: 75/75	SBP + ③	③	2w	ER; FEV_1%pred	NR	NR
Zheng X 2018	I: 59.27 ± 16.33; C: 62.13 ± 12.38	I: 29/25; C: 33/21	NR	I: 17.65 ± 8.34; C: 18.54 ± 8.21	I: 54/50; C: 54/50	SZJQ + ③	③	2w	FEV_1; FEV_1/FVC; SGRQ	NR	NR
Cai B 2018	I: 55.14 ± 4.12; C: 55.24 ± 4.21	I: 18/17; C: 19/16	NR	I: 6.12 ± 2.11; C: 6.21 ± 2.21	I: 35/35; C: 35/35	SZJQ + ④	④	2w	ER; FEV_1%pred	0	NR

Yuan C 2021	I: 63.46 ± 8.67; C: 64.14 ± 7.72	I: 33/15; C: 31/17	NR	I: 17.44 ± 7.61; C: 16.53 ± 7.79	I: 48/46; C: 48/46	SZJQ + ④	④	14d	ER; FEV_1; FEV_1%pred; FEV_1/FVC; SGRQ	I: 0 C: 1	6m
Chen DM 2020	I: 58.17 ± 5.91; C: 58.33 ± 5.99	I: 40/29; C: 44/25	3-4	I: 6.65 ± 1.28; C: 6.63 ± 1.41	I: 69/69; C: 69/69	SZJQ + ④	④	14d	ER; FEV_1; FEV_1/FVC; PaO_2; $PaCO_2$	0	NR
Li YH 2023	I: 58.52 ± 3.94; C: 59.13 ± 4.04	I: 24/16; C: 23/17	NR	I: 3.41 ± 0.34; C: 3.37 ± 0.44	I: 40/40; C: 40/40	SZJQ + ②	②	7d	ER; FEV_1; FEV_1%pred; FEV_1/FVC	NR	14d
Jiang MH 2021	I: 56.14 ± 4.72; C: 55.97 ± 5.33	I: 25/22; C: 27/20	NR	NR	I: 47/47; C: 47/47	SZJQ + ④	④	14d	ER; FEV_1; FEV_1/FVC; PaO_2; $PaCO_2$	NR	NR
Yan HM 2020	I: 64.97 ± 5.48; C: 65.43 ± 5.46	I: 24/18; C: 26/16	NR	NR	I: 42/42; C: 42/42	SZJQ + ③	③	14d	ER; FEV_1%pred; PaO_2; $PaCO_2$	NR	NR
Wang L 2017	I: 56.0 ± 3.0; C: 55.0 ± 3.3	I: 19/21; C: 20/20	NR	NR	I: 40/40; C: 40/40	SZJQ + ③	③	14d	FEV_1; FEV_1/FVC; PaO_2; $PaCO_2$	NR	NR
Jiang L 2018	I: 65.31 ± 9.67; C: 65.23 ± 9.58	I: 27/21; C: 28/20	1-3	I: 8.35 ± 3.40; C: 8.46 ± 3.37	I: 48/48; C: 48/48	SZJQ + ③	③	7d	ER; FEV_1; FEV_1/FVC; PaO_2; $PaCO_2$	NR	NR
Chen GL 2024	I: 62.21 ± 3.18; C: 62.24 ± 3.15	I: 23/17; C: 22/18	NR	I: 5.21 ± 0.48; C: 5.24 ± 0.45	I: 40/40; C: 40/40	SZJQ + ②	②	14d	ER; FEV_1; FEV_1/FVC; PaO_2; $PaCO_2$	NR	NR
Wang LY 2022	I: 61.50 ± 2.50; C: 61.42 ± 2.45	I: 27/12; C: 24/15	NR	I: 8.05 ± 1.17; C: 7.94 ± 1.08	I: 39/39; C: 39/39	SZJQ + ④	④	14d	ER; FEV_1; FEV_1/FVC; PaO_2; $PaCO_2$	I: 1 C: 5	NR
Yang BJ 2018	I: 65.24 ± 3.17; C: 65.37 ± 3.24	I: 25/25; C: 26/24	NR	I: 6.07 ± 1.19; C: 6.17 ± 1.27	I: 50/50; C: 50/50	SZJQ + ③	③	2w	ER; FEV_1; FEV_1/FVC	NR	NR
Liu JH 2024	I: 63.64 ± 4.03; C: 63.19 ± 3.72	I: 24/15; C: 20/19	2-3	I: 6.34 ± 1.45; C: 6.08 ± 1.16	I: 39/39; C: 39/39	SZJQ + ④	④	14d	ER; FEV_1; FEV_1/FVC; PaO_2; $PaCO_2$	0	NR
Mao ZX 2019	I: 74.5 ± 7.8; C: 74.3 ± 7.6	I: 35/35; C: 36/24	2-3	NR	I: 60/60; C: 60/60	SZJQ + ④	④	2w	ER; FEV_1%pred; FEV_1/FVC; PaO_2; $PaCO_2$	0	NR
Zhang LH 2011	I: 65.2 C: 67.5	I: 21/19; C: 22/18	NR	I: 22.6; C: 25.6	I: 40/40; C: 40/40	SZJQ + ③	③	7d	ER; FEV_1; FEV_1/FVC	I: 0 C: 2	NR
Dai LF 2023	I: 57.36 ± 7.36; C: 56.96 ± 6.69	I: 25/19; C: 23/21	2-3	I: 8.14 ± 3.69; C: 7.89 ± 2.27	I: 44/44; C: 44/44	SZJQ + ④	④	10d	ER; FEV_1; FEV_1/FVC; PaO_2; $PaCO_2$	NR	NR
Deng HX 2013	I: 55.2 ± 4.8; C: 56.3 ± 6.9	I: 24/16; C: 30/10	NR	I: 6.0 ± 2.3; C: 6.9 ± 2.5	I: 40/40; C: 40/40	SZJQ + ④	④	2w	ER; FEV_1%pred	NR	NR
Fu JY 2018	I: 59.1 ± 4.5; C: 58.9 ± 4.4	I: 21/20; C: 22/19	NR	NR	I: 41/41; C: 41/41	SZJQ + ③	③	7d	ER; FEV_1; FEV_1/FVC	NR	NR
Yuan LD 2017	I: 68.89 ± 8.61; C: 69.02 ± 8.42	I: 26/15; C: 27/13	1-4	I: 12.08 ± 4.66; C: 11.85 ± 4.37	I: 41/41; C: 40/40	WJ + ⑤	⑤	14d	ER; FEV_1; FEV_1/FVC	NR	NR
Zhang LS 2011	67.7 ± 7.1	59/21	NR	NR	I: 40/40; C: 40/40	WJ + ③	③	10d	ER; FEV_1; FEV_1%pred	I: 10 C: 8	NR
Liu SN 2022	I: 59.02 ± 10.11; C: 58.97 ± 10.15	I: 30/28; C: 31/27	2-3	I: 6.05 ± 0.51; C: 5.98 ± 0.48	I: 58/58; C: 58/58	WJ + ③	③	2w	FEV_1; FEV_1%pred; PaO_2; $PaCO_2$	NR	NR
Chen XJ 2019	I: 56.82 ± 8.35; C: 56.59 ± 6.42	I: 34/29; C: 32/31	2-3	I: 13.56 ± 4.64; C: 14.21 ± 4.56	I: 63/63; C: 63/63	XQL + ③	③	2w	ER; FEV_1; FEV_1%pred; PaO_2; $PaCO_2$	I: 4 C: 3	NR
Zhang HL 2023	I: 62.8 ± 3.2; C: 62.2 ± 3.1	I: 21/16; C: 20/17	NR	I: ＜5: 15; 5-10: 16；＞10: 6 C: ＜5: 17; 5-10: 15; ＞10: 5	I: 37/37; C: 37/37	XQL + ④	④	2w	ER; FEV_1	NR	NR
Wang S 2013	I: 62.7 ± 8.3; C: 63.4 ± 8.6	I: 28/12; C: 27/13	NR	I: 11.8 ± 3.7; C: 12.1 ± 3.9	I: 40/40; C: 40/40	XQL + ③	③	5d	ER; FEV_1; PaO_2; $PaCO_2$	NR	NR
Wu YW 2015	I: 58.4 ± 11.6; C: 60.2 ± 10.1	I: 27/21; C: 26/22	NR	I: 18.2 ± 12.2; C: 19.3 ± 11.3	I: 48/48; C: 48/48	XQL + ⑤	⑤	4w	ER; FEV_1	NR	NR

Liu YX 2013	I: 58.21 ± 6.19; C: 56.39 ± 4.51	I: 30/16; C: 34/12	NR	I: 13.24 ± 2.17; C: 11.71 ± 6.23	I: 46/43; C: 46/42	XQL + ④	④	14d	FEV_1; FEV_1/FVC	NR	NR
Ren X 2021	I: 60.6 ± 2.9; C: 61.2 ± 3.0	I: 20/18; C: 22/16	NR	NR	I: 38/38; C: 38/38	XQL + ⑤	⑤	14d	ER; FEV_1%pred; FEV_1/FVC	NR	NR
Chen R 2021	I: 54.62 ± 4.49; C: 54.07 ± 4.85	I: 28/19; C: 29/18	NR	I: 3.42 ± 0.73; C: 3.07 ± 0.61	I: 47/47; C: 47/47	XQL + ④	④	2w	ER; FEV_1%pred; FEV_1/FVC	0	NR
Zhu YL 2019	I: 69.12 ± 1.24; C: 69.59 ± 1.74	I: 29/21; C: 30/20	NR	NR	I: 50/50; C: 50/50	XQL + ③	③	10d	FEV_1; FEV_1%pred; FEV_1/FVC	NR	NR
Han ZQ 2014	69 ± 5.27	72/38	NR	13.60 ± 0.95	I: 68/68; C: 42/42	XQL + ④	④	12w	FEV_1; FEV_1%pred; FEV_1/FVC; PaO_2; $PaCO_2$	NR	NR
Tan YL 2017	I: 56.49 ± 5.32; C: 54.25 ± 5.85	I: 21/19; C: 26/14	NR	I: 5.54 ± 0.34; C: 5.64 ± 0.99	I: 40/40; C: 40/40	XQL + ④	④	10d	ER; FEV_1; FEV_1/FVC	NR	NR
Wang HM 2023	I: 60.98 ± 7.29; C: 61.36 ± 6.83	I: 21/14; C: 20/15	1-3	I: 11.04 ± 4.24; C: 10.77 ± 4.58	I: 35/35; C: 35/35	XQL + ④	④	7d	ER; FEV_1; FEV_1/FVC	0	NR
Zhang Y 2020	I: 61.5 ± 2.4; C: 61.3 ± 2.7	I: 25/20; C: 26/19	1-3	I: 3.5 ± 0.3; C: 3.3 ± 0.6	I: 45/45; C: 45/45	XQL + ③	③	10d	ER; FEV_1; FEV_1/FVC; PaO_2; $PaCO_2$	NR	NR
Ni XQ 2024	I: 58.13 ± 3.94; C: 57.35 ± 3.59	I: 24/16; C: 22/18	NR	I: 10.18 ± 3.10; C: 10.18 ± 3.05	I: 40/40; C: 40/40	XQL + ③	③	2w	ER; FEV_1; FEV_1/FVC	NR	NR
Zhang MC 2022	I: 54.96 ± 4.45; C: 55.74 ± 4.83	I: 29/20; C: 28/21	NR	I: 3.60 ± 0.85; C: 3.15 ± 0.76	I: 49/49; C: 49/49	XQL + ④	④	2w	ER; FEV_1%pred; FEV_1/FVC	I: 8 C: 9	NR
Luo ZQ 2012	58.44 ± 4.2	62/56	NR	5.2 ± 1.3	I: 59/59; C: 59/59	XQL + ③	③	10d	ER; FEV_1; FEV_1%pred; FEV_1/FVC	NR	NR
Shi YJ 2015	I: 65.4 ± 3.5; C: 66.9 ± 4.1	I: 27/23; C: 29/21	NR	I: 15.8 ± 4.7; C: 14.9 ± 4.4	I: 50/50; C: 50/50	XQL + ④	④	7d	FEV_1; FEV_1/FVC; PaO_2; $PaCO_2$	NR	NR
Zou WB 2017	I: 66.9 ± 7.5; C: 67.8 ± 8.2	I: 47/29; C: 46/30	2-3	I: 9.6 ± 2.4; C: 9.3 ± 2.7	I: 76/76; C: 76/76	XQL + ④	④	14d	ER; FEV_1; FEV_1/FVC; PaO_2; $PaCO_2$	NR	NR
Li JL 2015	I: 59.27 ± 7.14; C: 58.33 ± 6.89	I: 24/20; C: 24/19	NR	I: 10.25 ± 3.11; C: 10.33 ± 2.95	I: 44/44; C: 43/43	XQL + ③	③	28d	ER; FEV_1; FEV_1/FVC	0	NR
Sun HW 2021	I: 63.97 ± 7.05; C: 64.28 ± 7.11	I: 20/15; C: 21/14	NR	I: 3.75 ± 1.03; C: 3.82 ± 1.05	I: 35/35; C: 35/35	XQL + ④	④	10d	ER; FEV_1/FVC	I: 4 C: 3	NR
Zhu CW 2023	I: 67.89 ± 3.07; C: 67.98 ± 3.19	I: 28/18; C: 29/17	NR	I: 10.33 ± 2.87; C: 10.51 ± 2.92	I: 46/46; C: 46/46	XQL + ④	④	14d	ER; FEV_1; FEV_1/FVC; PaO_2; $PaCO_2$	NR	NR
Yu L 2015	I: 48.4 ± 11.6; C: 47.8 ± 8.54	I: 23/17; C: 24/16	NR	I: 0.6 - 30 C: 0.5 - 30	I: 40/40; C: 40/40	XQL + ②	②	2w	ER; FEV_1%pred; FEV_1/FVC	NR	NR
Xu XM 2016	I: 55.1 ± 5.3; C: 54.7 ± 5.2	I: 35/23; C: 36/22	NR	I: 7.6 ± 2 3; C: 7.4 ± 2.3	I: 58/58; C: 58/58	XQL + ②	②	2w	ER; FEV_1; PaO_2; $PaCO_2$	NR	NR
Chang QJ 2016	I: 68.6 ± 5.5; C: 68.4 ± 5.9	I: 26/16; C: 25/17	2-3	NR	I: 42/42; C: 42/42	XBCQ + ③	③	14d	ER; FEV_1%pred; FEV_1/FVC	NR	NR
Gu YY 2019	I: 65.9 ± 6.4; C: 65.7 ± 6.6	I: 26/18; C: 24/20	NR	I: 2.2 ± 0.5; C: 2.1 ± 0.4	I: 44/44; C: 44/44	XBCQ + ②	②	2w	ER; FEV_1/FVC	NR	NR
Fu YX 2016	I: 68.45 ± 9.66; C: 68.69 ± 11.11	I: 30/16; C: 33/13	NR	2-3	I: 46/46; C: 46/46	XBCQ + ③	③	2w	ER; FEV_1%pred; FEV_1/FVC	NR	NR
Zhuang X 2020	I: 65.28 ± 8.72; C: 63.25 ± 9.75	I: 18/27; C: 16/29	NR	NR	I: 45/45; C: 45/45	SBP + ②	②	10d	FEV_1; FEV_1/FVC	NR	NR
Ji XX 2020	I: 65.3 ± 7.2; C: 65.7 ± 7.5	I: 57/43; C: 56/44	1-4	I: 12.6 ± 1.9; C: 12.5 ± 1.6	I: 40/40; C: 40/40	YBBX + ③	③	14d	FEV_1; PaO_2; $PaCO_2$	NR	NR
Xiao LS 2021	I: 62.0 ± 14.0; C: 64.1 ± 12.0	I: 31/29; C: 30/30	2	I: 10.6 ± 5.0; C: 12.5 ± 4.5	I: 60/60; C: 60/60	YBBX + ⑤	⑤	7d	ER; FEV_1; FEV_1/FVC	NR	NR
Wang PC 2012	I: 45-82; C: 47-83	I: 25/10; C: 24/10	1-3	I: 4-25; C: 3-28	I: 35/35; C: 35/35	YBBX + ③	③	2w	FEV_1%pred; FEV_1/FVC	NR	NR
Guo WX 2007	I: 50-86; C: 52-82	I: 20/15; C: 18/17	NR	I: 10-40; C: 10-38	I: 35/35; C: 35/35	XBCQ + ③	③	2w	ER; FEV_1%pred	NR	NR

Liu H 2017	I: 65.24 ± 8.32;<br>C: 64.89 ± 8.14	I: 23/17; C: 24/16	NR	I: 5.84 ± 3.01;<br>C: 5.84 ± 3.01	I: 40/40; C: 40/40	XQL + ③	③	7d	ER; FEV_1%pred; FEV_1/FVC	NR	NR
Zhang WZ 2014	I: 65.24 ± 8.32;<br>C: 65.48 ± 8.76	I: 20/15; C: 22/13	NR	NR	I: 35/35; C: 35/35	SZJQ + ③	③	7d	ER; FEV_1%pred; PaO_2; $PaCO_2$	0	NR
Wu CH 2015	57.16 ± 2.92	57/43	1-2	2-9	I: 50/50; C: 50/50	WJ + ③	③	10d	ER; FEV_1; FEV_1%pred; FEV_1/FVC	0	NR
Wang YX 2017	I: 53.2 ± 2.1;<br>C: 54.5 ± 2.2	58/32	NR	NR	I: 45/45; C: 45/45	YBBX + ③	③	14d	ER; FEV_1%pred; PaO_2; $PaCO_2$	NR	NR
Hua WS 2017	61.57 ± 6.54	47/33	NR	15 ± 3	I: 40/40; C: 40/40	MXSG + ③	③	14d	ER; FEV_1%pred; FEV_1/FVC	NR	NR
Han WL 2025	I: 70.02 ± 1.05;<br>C: 70.01 ± 1.03	I: 24/16; C: 21/19	APACHE-II:<br>T: 15.27 ± 3.11;<br>C: 15.06 ± 3.10	I: 2.33 ± 0.15;<br>C: 2.32 ± 0.14	I: 40/40; C: 40/40	DC + ③	③	21d	ER; FEV_1	I: 7<br>C: 4	NR
Lu GL 2025	I: 66.25 ± 4.12;<br>C: 65.43 ± 3.55	I: 33/13; C: 31/15	NR	I: 6.86 ± 1.24;<br>C: 7.16 ± 1.32	I: 46/46; C: 46/46	XQL + ②	②	14d	FEV_1%pred; CAT	NR	NR
Le X 2025	I: 56.53 ± 1.77;<br>C: 56.56 ± 1.75	I: 24/16; C: 22/18	NR	T: 8.60 ± 1.89;<br>C: 8.63 ± 1.92	I: 40/40; C: 40/40	SZJQ + ①	①	14d	ER; FEV_1; FEV_1%pred	I: 6<br>C: 4	NR
Qiu ZF 2024	I: 60.45 ± 2.26;<br>C: 60.31 ± 2.40	NR	NR	T: 1.69 ± 0.21;<br>C: 1.69 ± 0.22	I: 36/36; C: 36/36	XQL + ②	②	14d	ER; FEV_1	0	NR

I/C: intervention/control groups, ①: no core medication group (symptomatic treatment, without any of: bronchodilators, antibiotics, or corticosteroids), ②: monotherapy group (using only one class among: bronchodilators, antibiotics, or corticosteroids), ③: dual-therapy group (using any two classes among: bronchodilators, antibiotics, or corticosteroids), ④: triple-therapy group (using all three classes: bronchodilators, antibiotics, and corticosteroids), ⑤: unspecified-regimen group (original description too vague for classification), ER: effective rate, CAT: Chronic Obstructive Pulmonary Disease Assessment Test, SGRQ: St. George's Respiratory Questionnaire, CCQ: Clinical COPD Questionnaire, y: year, m: month, w: week, d: day, NR: not reported.

[a]The severity according to the GOLD (Global Initiative for Chronic Obstructive Lung Disease) classification criteria.

References:

1．Bai, F. R., Xu, D. (2024). Efficacy Observation of Modified Suzi Jiangqi Decoction Combined with Montelukast Sodium in the Treatment of Acute Exacerbation of Chronic Obstructive Pulmonary Disease with Phlegm - turbidity Obstructing the Lung Syndrome. *Acta Chinese Medicine and Pharmacology*, 52(03), 66 - 70. doi: 10.19664/j.cnki.1002 - 2392.240056

2．Bai, Y. L., Gao, C. (2018). Analysis of the Clinical Value of Modified Qianjin Weijing Decoction in Acute Exacerbation of Chronic Obstructive Pulmonary Disease. *World Latest Medicine Information Digest*, 18(40), 168 + 175. doi: 10.19613/j.cnki.1671 - 3141.2018.40.137

3．Cai, B., Ye, R. Y., Zhang, Z. L., Sun, S., Xie, H. J., Lin, H. (2018). Influence of Suzi Jiangqi Decoction on Pulmonary Function of Patients with Acute Exacerbation of Chronic Obstructive Pulmonary Disease. *Shenzhen Journal of Integrated Traditional Chinese and Western Medicine*, 28(03), 34 - 36. doi: 10.16458/j.cnki.1007 - 0893.2018.03.017

4．Zeng, Y. Z., Jiang, H., Tong, H. M. (2024). Efficacy of Dingchuan Decoction Combined with Budesonide in the Treatment of Acute Attack of COPD. *Chinese Journal of Practical Village Doctors*, 31(05), 19 - 22 + 26.

5．Chang, Q. J. (2016). Clinical Analysis of Xuanbai Chengqi Decoction in the Treatment of Acute Exacerbation of Chronic Obstructive Pulmonary Disease. *China Health Standard Management*, 7(36), 114 - 115.

6．Chen, D. M., Wang, J. Y. (2020). Clinical Observation of 69 Cases of Suzi Jiangqi Decoction in the Adjuvant Treatment of Acute Exacerbation of Chronic Obstructive Pulmonary Disease. *Journal of Anhui University of Chinese Medicine*, 39(05), 25 - 29.

7．Chen, G. L., Hu, D., Jin, C. J. (2024). Efficacy of Modified Suzi Jiangqi Decoction in the Treatment of Acute Exacerbation of Chronic Obstructive Pulmonary Disease. *Diet Health*, (7), 141 - 144.

8．Chen, L., Zhai, J. H., Xia, Q. Q. (2018). Modified Sangbaipi Decoction in the Treatment of Acute Exacerbation of Chronic Obstructive Pulmonary Disease. *Shenzhen Journal of Integrated Traditional Chinese and Western Medicine*, 28(22), 59 - 61. doi: 10.16458/j.cnki.1007 - 0893.2018.22.025

9．Chen, R., Li, H. J., Li, E., Fan, L., Zhang, L. L. (2021). Effects of Modified Xiaoqinglong Decoction on TGF - β1, YKL - 40, MMP - 9 and Pulmonary Function in Patients with Acute Phase of Chronic Obstructive Pulmonary Disease (External Cold and Internal Fluid - Retention Type). *Pharmacology and Clinics of Chinese Materia Medica*, 37(04), 184 - 188. doi: 10.13412/j.cnki.zyyl.2021.04.023

10．Chen, W. S., Feng, C., Li, C. Y. (2024). Effects of Modified Xuanbai Chengqi Decoction on Serum IL - 21, CTRP - 9 and IL - 33 in Patients with Chronic Obstructive Pulmonary Disease. *Journal of Emergency in Traditional Chinese Medicine*, 33(01), 92 - 95.

11．Chen, X. J. (2019). Effects of Xiaoqinglong Decoction on Blood Gas Analysis Indexes and Pulmonary Function in Patients with COPD during the Attack Period. *Acta Chinese Medicine*, 34(02), 400 - 403. doi: 10.16368/j.issn.1674 - 8999.2019.02.094

12．Dai, Y. M. (2013). Randomized Parallel Controlled Study on Qianjin Weijing Decoction Combined with Western Medicine in the Treatment of Acute Exacerbation of Chronic Obstructive Pulmonary Disease. *Journal of Practical Traditional Chinese Internal Medicine*, 27(03), 66 - 67.

13．Dai, L. F. (2023). Clinical Study on Suzi Jiangqi Decoction Combined with Western Medicine in the Treatment of Acute Exacerbation of Chronic Obstructive Pulmonary Disease. *Journal of New Chinese Medicine*, 55(06), 6 - 10. doi: 10.13457/j.cnki.jncm.2023.06.002

14．Deng, H. X., Xu, G. (2013). Clinical Observation of 80 Cases of Suzi Jiangqi Decoction in the Treatment of Acute Exacerbation of Chronic Obstructive Pulmonary Disease. *Inner Mongolia Journal of Traditional Chinese Medicine*, 32(32), 20. doi: 10.16040/j.cnki.cn15 - 1101.2013.32.046

15．Duan, W. Y. (2021). Clinical Observation on Modified Maxing Shigan Decoction in the Treatment of Acute Attack of Chronic Obstructive Pulmonary Disease. *Kangyi*, (17), 165 - 166.

16．Fan, P. C., Zhu, M. J. (2020). Exploration of the Effect of Erchen Decoction Combined with Sanzi Yangqin Decoction in the Treatment of Acute Exacerbation of Chronic Obstructive Pulmonary Disease. *Healthful Friend*, (13), 154.

17．Fei, X., Ren, L. L., Dang, Y. E. (2024). Efficacy Observation of Sangbaipi Decoction as Adjuvant Therapy in the Treatment of Acute Exacerbation of Chronic Obstructive Pulmonary Disease with Phlegm - heat Accumulating in the Lung Syndrome. *Forum on Traditional Chinese Medicine*, 39(03), 39 - 41. doi: 10.13913/j.cnki.41 - 1110/r.2024.03.012

18．Feng, J., Liu, Q. (2023). Analysis of the Efficacy of Erchen Decoction Combined with Sanzi Yangqin Decoction in the Treatment of Acute Exacerbation of Chronic Obstructive Pulmonary Disease. *Self - Care*, (10), 144 - 146.

19．Feng, J., Sun, G. (2018). Effects of Modified Weijing Decoction on Serum C - reactive Protein, Interleukin - 6 and Interleukin - 10 Levels in the Treatment of Acute Exacerbation of Chronic Obstructive Pulmonary Disease with Phlegm - heat Accumulating in the Lung Syndrome. *Journal of Hunan University of Chinese Medicine*, 38(A01).

20．Fu, Y. X., Wu, J. T., Gu, C. L. (2016). Clinical Observation of Xuanbai Chengqi Decoction in the Treatment of Acute Exacerbation of Chronic Obstructive Pulmonary Disease (Phlegm - heat Accumulating in the Lung Syndrome). *Clinical Journal of Traditional Chinese Medicine*, 8(25), 87 - 88.

21．Fu, J. Y. (2018). Suzi Jiangqi Decoction as Adjuvant Therapy in 41 Cases of Acute Attack of Chronic Obstructive Pulmonary Disease. *Forum on Traditional Chinese Medicine*, 33(01), 51 - 52. doi: 10.13913/j.cnki.41 - 1110/r.2018.01.025

22．Gao, B. J. (2022). Study on the Effect of Modified Sangbaipi Decoction Combined with Atomization Inhalation in the Treatment of Acute Exacerbation of Elderly Chronic Obstructive Pulmonary Disease. *China Science and Technology Journal Database Medicine*, (5).

23．Gao, Z. T., Chen, Y. (2022). Efficacy of Modified Maxing Shigan Decoction in the Treatment of Acute Attack of Chronic Obstructive Pulmonary Disease. *Chinese Community Doctors*, 38(08), 49 - 51.

24．Gong, Y. (2022). Effect of Modified Maxing Shigan Decoction Combined with Tiotropium Bromide Inhalation Powder Aerosol in the Treatment of Patients with Chronic Obstructive Pulmonary Disease. *Medical Journal of Chinese People's Health*, 34(21), 90 - 93.

25．Gu, Y. Y. (2019). Clinical Observation of Modified Xuanbai Chengqi Decoction in the Treatment of Acute Exacerbation of Chronic Obstructive Pulmonary Disease. *Chinese Journal of Traditional Medical Science and Technology*, 26(03), 429 - 430.

26．Guan, Z. Y. (2021). Effects of Erchen Decoction Combined with Sanzi Yangqin Decoction on Acute Exacerbation of Chronic Obstructive Pulmonary Disease (Phlegm - turbidity Accumulating in the Lung Syndrome). *Clinical Journal of Traditional Chinese Medicine*, 13(27), 23 - 25 + 35.

27．Guo, W. X., Zhang, F. Y. (2007). Influence of Integrated Traditional Chinese and Western Medicine Treatment on Pulmonary Function and Cytokines in Chronic Obstructive Pulmonary Disease. *China Journal of Traditional Chinese Medicine and Pharmacy*, 22(6).

28．Han, Z. Q., Xu, W. N. (2014). Efficacy Observation of Modified Xiaoqinglong Decoction Combined with Spiriva in the Treatment of Acute Exacerbation of COPD. *World Latest Medicine Information Digest (Continuous Electronic journal)*, (36), 309 - 310.

29．Hao, S. R., Wu, Q. L., Cao, X. L. (2018). Clinical Observation of Qianjin Weijing Decoction Combined with Combivent and Ambroxol Aerosol Inhalation in Elderly Patients with Acute Exacerbation of Chronic Obstructive Pulmonary Disease. *Hebei Medical Journal*, 40(24), 3708 - 3711.

30．He, C. N. (2017). Exploration of the Efficacy of Modified Maxing Shigan Decoction in the Treatment of Acute Exacerbation of Chronic Obstructive Pulmonary Disease (AECOPD). *World Latest Medicine Information Digest*, 17(22), 82 + 85.

31．Hua, W. S. (2017). Efficacy Observation of Integrated Traditional Chinese and Western Medicine in the Treatment of Acute Exacerbation of Chronic Obstructive Pulmonary Disease. *Journal of Practical Traditional Chinese Medicine*, 33(1), 41.

32．Huang, F., Wen, X. F., Shen, Q. (2012). Clinical Efficacy Observation of Maxing Shigan Decoction in the Treatment of Acute Exacerbation of Chronic Obstructive Pulmonary Disease. *China Pharmacy*, 23(31), 2955 - 2956.

33．Ji, X. X., Chen, X., Zhu, W. M. (2020). Exploration of the Efficacy and Mechanism of Yuebi plus Banxia Decoction in Elderly Patients with Acute Exacerbation of Chronic Obstructive Pulmonary Disease. *Contemporary Medicine*, 26(07), 137 - 139.

34．Jiang, L., Zhang, W. (2018). Effect Observation of Modified Suzi Jiangqi Decoction in the Treatment of Acute Exacerbation of Chronic Obstructive Pulmonary Disease. *The Medical Forum*, 22(07), 968 - 969. doi: 10.19435/j.1672 - 1721.2018.07.066

35．Jiang, M. H. (2021). Clinical Value of Modified Suzi Jiangqi Decoction in the Treatment of Acute Exacerbation of Chronic Obstructive Pulmonary Disease. *China Practical Medicine*, 16(07), 172 - 174. doi: 10.14163/j.cnki.11 - 5547/r.2021.07.075

36．Jiang, L. X., Jiang, W. W. (2021). Clinical Study on Modified Suzi Jiangqi Decoction Combined with Western Medicine in the Treatment of Acute Exacerbation of Chronic Obstructive Pulmonary Disease. *Journal of New Chinese Medicine*, 53(16), 9 - 12. doi: 10.13457/j.cnki.jncm.2021.16.003

37．Kou, Z. J. (2024). Effect of Modified Qingqi Huatan Decoction in the Treatment of Patients with Acute Exacerbation of Chronic Obstructive Pulmonary Disease. *Diet Health*, (20), 53 - 56.

38．Lan, H. (2019). Effect Observation of Modified Maxing Shigan Decoction in the Treatment of Acute Exacerbation of Chronic Obstructive Pulmonary Disease. *Contemporary Medical Symposium*, 17(02), 179 - 180.

39．Li, J. L., Fan, C., Wang, Q. (2015). Randomized Parallel Controlled Study on Xiaoqinglong Decoction Combined with Western Medicine in the Treatment of the Attack Stage of Chronic Obstructive Pulmonary Disease. *Journal of Practical Traditional Chinese Internal Medicine*, 29(10), 124 - 126. doi: 10.13729/j.issn.1671 - 7813.2015.10.53

40．Li, Y. H., Tan, M. L., Lu, J. H. (2023). Influence of Modified Suzi Jiangqi Decoction Combined with Compound Ipratropium Bromide on Airflow Limitation and Fas/Apo - 1, TNF - α in AECOPD Patients. *Heilongjiang Medicine Journal*, 36(04), 780 - 783. doi: 10.14035/j.cnki.hljyy.2023.04.011

41．Liang, Z. X., Zhong, X. B. (2023). Effect Observation of Modified Sangbaipi Decoction Combined with Aerosol Inhalation in the Treatment of Acute Exacerbation of Chronic Obstructive Pulmonary Disease. *Medical Journal of the Chinese People's Armed Police Forces in Xinjiang Production and Construction Corps*, 21(04), 49 - 50.

42．Liao, Q. (2020). Clinical Efficacy of Modified Maxing Shigan Decoction in the Treatment of Acute Exacerbation of Chronic Obstructive Pulmonary Disease. *Chinese Journal of Clinical Rational Drug Use*, 13(27), 160 - 161. doi: 10.15887/j.cnki.13 - 1389/r.2020.27.069

43．Lin, C. B., Zheng, L. L., Lin, Z. S. (2018). Clinical Effect Observation of Modified Maxing Shigan Decoction in the Treatment of Acute Attack of Chronic Obstructive Pulmonary Disease. *China Prescription Drug*, 16(03), 108 - 109.

44．Lin, F., Zhou, Q. Q., Fu, Y. Q., Liao, X. (2024). Influence of Dingchuan Decoction on Pulmonary Function and CAT Score of Patients with Acute Exacerbation of Chronic Obstructive Pulmonary Disease. *Guangming Journal of Chinese Medicine*, 39(03), 533 - 535.

45．Liu, H. (2017). Efficacy Observation of Integrated Traditional Chinese and Western Medicine in the Treatment of Acute Attack Stage of Chronic Obstructive Pulmonary Disease with Cold Fluid Retention Syndrome. *Traditional Chinese Medicine Journal*, 16(2), 49 - 51.

46．Liu, J. J., Feng, J. S., Li, X., Li, J. M. (2021). Efficacy Observation of Modified Sangbaipi Decoction in the Treatment of Acute Exacerbation of Chronic Obstructive Pulmonary Disease (Phlegm - heat Accumulating in the Lung Type). *World Chinese Medicine*, 16(12), 1884 - 1889.

47．Liu, J. H. (2024). Clinical Study on Adjuvant Treatment of AECOPD with Modified Suzi Jiangqi Decoction. *Practical Clinical Journal of Integrated Traditional Chinese and Western Medicine*, 24(09), 81 - 84. doi: 10.13638/j.issn.1671 - 4040.2024.09.024

48．Liu, S. N., Yuan, B. (2022). Effect of Modified Weijing Decoction in the Treatment of Patients with Acute Exacerbation of Chronic Obstructive Pulmonary Disease and Its Influence on Immune Balance Regulation and Inflammatory Response Control. *Clinical Research and Practice*, 7(30), 128 - 130. doi: 10.19347/j.cnki.2096 - 1413.202230035

49．Liu, Y. C. (2022). Effect Analysis of Modified Sangbaipi Decoction Combined with Aerosol Inhalation in the Treatment of Elderly Patients with Acute Exacerbation of Chronic Obstructive Pulmonary Disease. *Healthmust - Readmagazine*, (22), 180 - 181, 186.

50．Liu, Y. X., Wang, F., Qu, J. L., Zeng, Z. Y. (2013). Influence of Xiaoqinglong Decoction on Cytokines and Pulmonary Function in Patients with Acute Attack Stage of Chronic Obstructive Pulmonary Disease. *Journal of New Chinese Medicine*, 45(07), 24 - 26. doi: 10.13457/j.cnki.jncm.2013.07.032

51．Liu, G. J. (2024). Application Observation of Modified Sangbaipi Decoction Combined with High - Flow Nasal Oxygen Therapy in Acute Exacerbation of Chronic Obstructive Pulmonary Disease. *Chinese Journal of Traditional Medical Science and Technology*, 31(04), 669 - 671.

52．Luo, Z. Q. (2012). Clinical Analysis of Xiaoqinglong Decoction Combined with Seretide in the Treatment of Acute Exacerbation of Chronic Obstructive Pulmonary Disease. *Chinese Journal of Ethnomedicine and Ethnopharmacy*, 21(21), 119 - 120.

53．Lv, T. (2014). Clinical Observation of Qingqi Huatan Decoction Combined with Western Medicine in the Treatment of Acute Stage of Chronic Obstructive Pulmonary Disease. *Journal of Emergency in Traditional Chinese Medicine*, 23(05), 972 - 973.

54．Ma, J. Q. (2022). Influence of Modified Xiaoqinglong Decoction on Pulmonary Function Indexes and Airway Inflammatory Level in Elderly AECOPD Patients with External Cold and Internal Fluid - Retention Syndrome. *Liaoning Medical Journal*, 36(1), 87 - 90.

55．Ma, Z. H., Ma, X. J., Peng, L. (2020). Clinical Observation of Modified Qingqi Huatan Decoction Combined with Western Medicine in the Treatment of Acute Exacerbation of Chronic Obstructive Pulmonary Disease. *Heilongjiang Traditional Chinese Medicine*, 49(04), 34 - 35.

56．Mao, Z. X. (2019). Clinical Efficacy of Suzi Jiangqi Decoction Combined with Salmeterol and Fluticasone Propionate Powder for Inhalation in the Treatment of Elderly Patients with Acute Exacerbation of Chronic Obstructive Pulmonary Disease. *Chinese Journal of Clinical Rational Drug Use*, 12(27), 100 - 101. doi: 10.15887/j.cnki.13 - 1389/r.2019.27.051

57．Mei, Y. (2022). Effectiveness Analysis of Modified Qianjin Weijing Decoction Combined with Budesonide in the Treatment of Phlegm - heat Accumulating in the Lung Syndrome of Acute Exacerbation of Chronic Obstructive Pulmonary Disease. *Journal of Basic Chinese Medicine at the Grass - roots Level*, 1(02), 26 - 30.

58．Ni, X. Q., Zhang, X. W., Chen, X. J., Song, W. C., Shen, Q. M. (2024). Clinical Efficacy Observation of Modified Xiaoqinglong Decoction Combined with Western Medicine in the Treatment of Phlegm - dampness Accumulating in the Lung Syndrome of Acute Exacerbation of COPD. *Chinese Journal of Health Preservation and Healthcare*, 42(09), 7 - 12.

59．Niu, J., Wang, X. B., Wang, H. Y. (2023). Influence of Adjuvant Treatment with Modified Sangbaipi Decoction on Patients with Acute Exacerbation of Chronic Obstructive Pulmonary Disease. *Practical Journal of Clinical Medicine*, 20(01), 31 - 35.
60．Pan, X. D., Fan, L., Lu, B. Q., Zhang, M. C. (2018). Influence of Modified Xiaoqinglong Decoction on Inflammatory Factors in COPD Patients with External Cold and Internal Fluid - Retention Syndrome at the Acute Attack Stage. *Liaoning Journal of Traditional Chinese Medicine*, 45(09), 1879 - 1882. doi: 10.13192/j.issn.1000 - 1719.2018.09.028
61．Qi, Y. X., Wu, M. P., Deng, S. J., Wei, K. F. (2016). Efficacy Observation of Erchen Decoction Combined with Sanzi Yangqin Decoction in the Treatment of Acute Exacerbation of Chronic Obstructive Pulmonary Disease. *World Chinese Medicine*, 11(11), 2278 - 2280 + 2285.
62．Qian, J. Y., Tao, J. F., Liu, Q., Wang, J. J. (2019). Treatment of 60 Cases of Chronic Obstructive Pulmonary Disease with Sanzi Yangqin Decoction Combined with Erchen Decoction. *Zhejiang Journal of Traditional Chinese Medicine*, 54(11), 800 - 801. doi: 10.13633/j.cnki.zjtcm.2019.11.010
63．Ren, X. (2021). Influence of Adjuvant Treatment with Xiaoqinglong Decoction on the Efficacy and Pulmonary Function of COPD Patients in the Acute Stage. *Chinese Science and Technology Journal Database (Full - text Edition) Medicine and Health*, (10).
64．Shi, Y. J., Ding, X. C., Xu, B. (2015). Clinical Study on Oral Administration of Xiaoqinglong Decoction Combined with Western Medicine in the Treatment of the Attack Stage of COPD. *Journal of Chinese Medicinal Materials*, 38(07), 1550 - 1552. doi: 10.13863/j.issn1001 - 4454.2015.07.055
65．Song, Z. C., Cui, C. B. (2020). Efficacy of Maxing Shigan Decoction Combined with Salbutamol in the Treatment of Chronic Obstructive Pulmonary Disease and Its Influence on Inflammatory Factors. *Chinese Archives of Traditional Chinese Medicine*, 38(10), 222 - 225. doi: 10.13193/j.issn.1673 - 7717.2020.10.052
66．Su, W. (2020). Analysis of the Clinical Effect of Modified Qingqi Huatan Decoction in the Treatment of Acute Exacerbation of Chronic Obstructive Pulmonary Disease with Phlegm - heat Accumulating in the Lung Syndrome and Its Influence on Pulmonary Function. *Healthcare & Wellness*, (14), 52 - 53.
67．Su, Z. X. (2019). Application Effect of Modified Suzi Jiangqi Decoction in Elderly Patients with Acute Exacerbation of Chronic Obstructive Pulmonary Disease of Phlegm - turbidity Obstructing the Lung Type. *Clinical Medicine*, 39(08), 121 - 124. doi: 10.19528/j.issn.1003 - 3548.2019.08.048
68．Sun, H. W., Tan, H. D. (2021). Treatment of Chronic Obstructive Pulmonary Disease with External Cold and Internal Fluid - Retention Syndrome by Xiaoqinglong Decoction Combined with Western Medicine. *Shenzhen Journal of Integrated Traditional Chinese and Western Medicine*, 31(09), 55 - 57. doi: 10.16458/j.cnki.1007 - 0893.2021.09.025
69．Sun, H. J. (2017). Efficacy Observation of Modified Maxing Shigan Decoction Combined with Adrenocortical Glucocorticoids in the Treatment of the Acute Stage of Chronic Obstructive Pulmonary Disease. *Modern Journal of Integrated Traditional Chinese and Western Medicine*, 26(01), 24 - 26 + 30.
70．Sun, X. S., Xu, G. L. (2015). Clinical Observation of Modified Maxing Shigan Decoction in the Treatment of Acute Attack of Chronic Obstructive Pulmonary Disease. *World Chinese Medicine*, 10(02), 199 - 201 + 205.
71．Tan, Y. L. (2017). Efficacy Analysis of Modified Xiaoqinglong Decoction Combined with Spiriva in the Treatment of Acute Exacerbation of Chronic Obstructive Pulmonary Disease. *World Latest Medicine Information Digest*, 17(73), 130 + 134. doi: 10.19613/j.cnki.1671 - 3141.2017.73.081
72．Tang, X. J., Lei, L. M. (2023). Clinical Observation of Modified Sangbaipi Decoction as Adjuvant Therapy in the Treatment of Acute Exacerbation of Chronic Obstructive Pulmonary Disease. *Journal of Practical Traditional Chinese Medicine*, 39(03), 548 - 550.
73．Wang, W., Wang, B., Zhang, L. C., Gao, F., Wu, W. (2021). Influence of Modified Maxing Shigan Decoction Combined with Tiotropium Bromide on Pulmonary Function, Blood Gas Indexes and Inflammatory Factors in Patients with Acute Exacerbation of Chronic Obstructive Pulmonary Disease. *Progress in Modern Biomedicine*, 21(06), 1046 - 1050. doi: 10.13241/j.cnki.pmb.2021.06.010
74．Wang, H. M., Li, H. T., Zhang, J., Gu, J. H. (2023). Efficacy of Modified Xiaoqinglong Decoction in the Treatment of Acute Attack of Chronic Obstructive Pulmonary Disease and Its Influence on Inflammatory Factors. *Henan Traditional Chinese Medicine*, 43(03), 340 - 344. doi: 10.16367/j.issn.1003 - 5028.2023.03.0068
75．Wang, H. X. (2023). Analysis of the Clinical Efficacy of Xiaoqinglong Decoction Combined with Routine Treatment in Patients with Acute Stage of Chronic Obstructive Pulmonary Disease of External Cold and Internal Fluid - Retention Type. *Healthmust - Readmagazine*, (1), 195 - 196.
76．Wang, L. Y. (2022). Effect Observation of Modified Suzi Jiangqi Decoction in the Treatment of Acute Exacerbation of Chronic Obstructive Pulmonary Disease and Analysis of Its Influence on TCM Syndrome Score. *Medical Diet and Health*, 20(18), 21 - 24.
77．Wang, L. (2017). Clinical Efficacy Observation of Modified Suzi Jiangqi Decoction in the Treatment of Acute Exacerbation of Chronic Obstructive Pulmonary Disease. *Chinese Journal of Modern Drug Application*, 11(23), 83 - 84. doi: 10.14164/j.cnki.cn11 - 5581/r.2017.23.048
78．Wang, P. C., Lin, X. H. (2012). Clinical Efficacy of Yuebi plus Banxia Decoction in the Treatment of Acute Exacerbation of Chronic Obstructive Pulmonary Disease of Phlegm - heat Accumulating in the Lung Type. *The Medical Frontier*, 02(11), 138 - 139.
79．Wang, S. (2013). Efficacy Observation of Xiaoqinglong Decoction in the Acute Exacerbation of Chronic Obstructive Pulmonary Disease Patients. *Chinese Journal of Modern Drug Application*, 7(11), 133 - 134. doi: 10.14164/j.cnki.cn11 - 5581/r.2013.11.014
80．Wang, S. C., Zhang, A. (2023). Effect Analysis of Erchen Decoction Combined with Sanzi Yangqin Decoction in the Treatment of Patients with Acute Exacerbation of Chronic Obstructive Pulmonary Disease. *Chinese Science and Technology Journal Database (Full - text Edition) Medicine and Health*, (4).
81．Wang, X. H., Yuan, Y. H. (2023). Clinical Study of Qingqi Huatan Decoction Combined with Western Medicine in the Treatment of Acute Stage of Chronic Obstructive Pulmonary Disease of Phlegm - heat Obstructing the Lung Type. *The Medical Forum*, 27(32), 121 - 123. doi: 10.19435/j.1672 - 1721.2023.32.041
82．Wang, X. B. (2020). Value Analysis of the Combined Use of Erchen Decoction and Sanzi Yangqin Decoction in the Treatment of Acute Exacerbation of Chronic Obstructive Pulmonary Disease. *China Continuing Medical Education*, 12(31), 166 - 169.
83．Wang, X. D. (2017). Clinical Efficacy Discussion of Modified Maxing Shigan Decoction in the Treatment of Patients with Acute Attack of Chronic Obstructive Pulmonary Disease. *Health Guide*, (38), 51, 82.
84．Wang, X. P. (2015). Maxing Shigan Decoction as Adjuvant Therapy in 39 Cases of Acute Exacerbation of Chronic Obstructive Pulmonary Disease and Nursing Measures. *China Pharmaceuticals*, 24(12), 93 - 95.
85．Wang, Y. X., Jiang, Y. H. (2017). Efficacy Observation of Integrated Traditional Chinese and Western Medicine in the Treatment of Acute Exacerbation of Chronic Obstructive Pulmonary Disease. *World Latest Medicine Information Digest*, 17(04), 131.
86．Wang, Z. F. (2017). Effect Observation of Modified Maxing Shigan Decoction as Adjuvant Therapy in the Treatment of Acute Exacerbation of Chronic Obstructive Pulmonary Disease. *Chinese Journal of Rural Medicine and Pharmacy*, 24(20), 58 - 59. doi: 10.19542/j.cnki.1006 - 5180.000889
87．Wei, J. Q. (2023). Clinical Efficacy Observation of Erchen Decoction Combined with Sanzi Yangqin Decoction in the Treatment of Acute Exacerbation of Chronic Obstructive Pulmonary Disease. *China Science and Technology Journal Database Medicine*, (4).
88．Wei, J., Li, Z. P., Hu, H. J. (2022). Clinical Observation of Erchen Decoction Combined with Sanzi Yangqin Decoction as Adjuvant Therapy in the Treatment of Acute Exacerbation of Chronic Obstructive Pulmonary Disease. *Journal of Practical Traditional Chinese Medicine*, 38(11), 1904 - 1905.
89．Wu, C. H., Zhou, Q. L. (2015). Efficacy Observation of Integrated Traditional Chinese and Western Medicine in the Treatment of Acute Attack Stage of Chronic Obstructive Pulmonary Disease. *Journal of New Chinese Medicine*, 47(12), 42 - 44. doi: 10.13457/j.cnki.jncm.2015.12.019
90．Wu, J. N., Wang, J. Y. (2016). Clinical Observation of Modified Dingchuan Decoction Combined with Compound Methoxyphenamine Capsules in the Treatment of Acute Exacerbation of COPD. *Journal of New Chinese Medicine*, 48(06), 43 - 45. doi: 10.13457/j.cnki.jncm.2016.06.019
91．Wu, M. (2021). Effectiveness Study of Dingchuan Decoction in the Treatment of Acute Attack Stage of Chronic Obstructive Pulmonary Disease. *China Health Care & Nutrition*, 31(17), 268 - 269.

92．Wu, Y. W., Liu, T., Liu, S. P. (2015). Influence of Xiaoqinglong Decoction on Pulmonary Function and Serum Cytokines in Patients with Acute Attack Stage of Chronic Obstructive Pulmonary Disease. *Medical Innovation of China*, 12(01), 109 - 111.

93．Xiao, L. S., Yuan, G. Z., Yang, X. C. (2021). Clinical Efficacy Observation of Yuebi plus Banxia Decoction in Patients with Acute Attack of Chronic Obstructive Pulmonary Disease (Phlegm - heat Stagnating in the Lung Type). *Clinical Journal of Traditional Chinese Medicine*, 13(07), 110 - 112.

94．Xie, G. B. (2023). Efficacy Observation of Modified Qingqi Huatan Decoction Combined with Budesonide in Patients with Acute Exacerbation of Chronic Obstructive Pulmonary Disease. *Chinese Journal of Health Preservation and Healthcare*, 41(16), 36 - 40.

95．Xie, W. H. (2009). Modified Maxing Shigan Decoction in the Treatment of Acute Exacerbation of Chronic Obstructive Pulmonary Disease. *Chinese Medicine Modern Distance Education of China*, 7(04), 101 - 102.

96．Xing, H. Z., Tian, Z. L. (2020). Observation on the Therapeutic Effect of Erchen Decoction Combined with Sanzi Yangqin Decoction on Acute Exacerbation of Chronic Obstructive Pulmonary Disease (COPD) and Its Influence on Inflammatory Factors and Pulmonary Function. *Smart Healthcare*, 6(27), 173 - 174 + 189. doi: 10.19335/j.cnki.2096 - 1219.2020.27.083

97．Xiong, D. N., Sun, C. H. (2016). Clinical Study of Modified Maxing Shigan Decoction Combined with Salmeterol and Fluticasone Propionate Inhalation in the Treatment of Acute Exacerbation of Chronic Obstructive Pulmonary Disease. *International Journal of Traditional Chinese Medicine*, (2), 118 - 122.

98．Xu, H., Zhu, J., Wang, Y. F. (2019). Influence of Erchen Decoction Combined with Sanzi Yangqin Decoction on Inflammatory Factors and Pulmonary Function in Patients with Acute Exacerbation of Chronic Obstructive Pulmonary Disease. *Journal of Emergency in Traditional Chinese Medicine*, 28(11), 1889 - 1892.

99．Xu, X. M. (2016). Clinical Observation of 58 Cases of Xiaoqinglong Decoction in the Treatment of Acute Exacerbation of Chronic Obstructive Pulmonary Disease. *Guangming Journal of Chinese Medicine*, 31(24), 3604 - 3606.

100．Xu, X. P. (2022). Efficacy Observation of Modified Maxing Shigan Decoction Combined with Western Medicine Routine Treatment in the Treatment of Acute Exacerbation of Chronic Obstructive Pulmonary Disease and Its Influence on Related Indicators. *Chinese Journal of Traditional Medical Science and Technology*, 29(05), 912 - 914.

101．Yan, H. M., Yu, J. H. (2020). Experience on the Clinical Value of Modified Suzi Jiangqi Decoction in the Treatment of Acute Exacerbation of Chronic Obstructive Pulmonary Disease. *Clinical Journal of Traditional Chinese Medicine*, 12(08), 28 - 30.

102．Yan, L. W. (2020). Efficacy Analysis of Erchen Decoction Combined with Sanzi Yangqin Decoction in the Treatment of Acute Exacerbation of Chronic Obstructive Pulmonary Disease with Phlegm - turbidity Obstructing the Lung Syndrome. *Electronic Journal of Integrated Traditional Chinese and Western Medicine in Cardio - Cerebrovascular Disease*, 8(33), 10 + 40. doi: 10.16282/j.cnki.cn11 - 9336/r.2020.33.008

103．Yan, S. L., Huo, J. (2019). Clinical Efficacy of Modified Maxing Shigan Decoction in the Treatment of Chronic Obstructive Pulmonary Disease and Its Influence on Pulmonary Function. *Special Health*, (21), 101 - 102.

104．Yan, H. F., Chen, L., Zeng, J. J., Rao, J., Gui, Y., Yan, W., et al. (2020). Efficacy and Influence on Lipid Metabolism of Modified Dingchuan Decoction Combined with Western Medicine Routine Treatment in Patients with Acute Exacerbation of Chronic Obstructive Pulmonary Disease of Phlegm - heat Obstructing Type. *Journal of Chinese Medicinal Materials*, 43(08), 2012 - 2016. doi: 10.13863/j.issn1001 - 4454.2020.08.041

105．Yang, B. J. (2018). Clinical Efficacy Evaluation of Modified Suzi Jiangqi Decoction with Whole Scorpion and Pheretima in the Treatment of AECOPD of Phlegm - turbidity Obstructing the Lung Type. *Practical Journal of Cardiac Cerebral Pneumal and Vascular Disease*, 26(S2), 234 - 235.

106．Yang, X. L., Shi, J. (2014). Modified Erchen Decoction Combined with Sanzi Yangqin Decoction in the Treatment of 46 Cases of Acute Exacerbation of Chronic Obstructive Pulmonary Disease. *Shaanxi Journal of Traditional Chinese Medicine*, 35(09), 1154 - 1155.

107．Yao, B. (2023). Clinical Observation of Modified Qingqi Huatan Decoction Combined with Western Medicine in the Treatment of Acute Exacerbation of Chronic Obstructive Pulmonary Disease. *Laboratory Medicine and Clinic*, 20(06), 736 - 738 + 742.

108．Yao, F. (2021). Effect Analysis of Modified Dingchuan Decoction in the Treatment of Acute Exacerbation of Chronic Obstructive Pulmonary Disease. *Contemporary Medical Symposium*, 19(13).

109．You, W. H. (2022). Effectiveness Study of Dingchuan Decoction in the Treatment of Acute Attack Stage of Chronic Obstructive Pulmonary Disease. *Inner Mongolia Journal of Traditional Chinese Medicine*, 41(12).

110．Yu, C., Liu, A., Li, A., Wang, A., Chen, A. (2023). Clinical Efficacy Observation of Erchen Decoction Combined with Sanzi Yangqin Decoction in the Treatment of Phlegm - turbidity Obstructing the Lung Syndrome in Patients with Acute Exacerbation of Chronic Obstructive Pulmonary Disease. *Chinese Science and Technology Journal Database (Full - text Edition) Medicine and Health*, (7).

111．Yu, L. (2015). Xiaoqinglong Decoction in the Treatment of 40 Cases of Acute Exacerbation of Chronic Obstructive Pulmonary Disease. *Chinese Medicine Modern Distance Education of China*, 13(11), 43 - 45.

112．Yu, X. D. (2022). Analysis of the Clinical Effect of Sanzi Yangqin Decoction Combined with Erchen Decoction in the Treatment of Patients with Acute Exacerbation of Chronic Obstructive Pulmonary Disease. *Doctor*, 7(8), 14 - 17.

113．Yuan, C., Zhu, Z. G. (2021). Clinical Efficacy of Suzi Jiangqi Decoction in the Treatment of Phlegm - turbidity Obstructing the Lung Syndrome during the Acute Phase of Chronic Obstructive Pulmonary Disease and Its Influence on Endocrine Function. *Journal of Chinese Medicinal Materials*, 44(05), 1239 - 1243. doi: 10.13863/j.issn1001 - 4454.2021.05.038

114．Yuan, L. D., Li, L. S., Meng, B. (2017). Clinical Efficacy of Modified Weijing Decoction in the Treatment of Patients with Acute Exacerbation of Chronic Obstructive Pulmonary Disease and Its Influence on TNF - α and IL - 8. *Harbin Medical Journal*, 37(02), 177 - 178.

115．Yue, Y. X. (2020). Clinical Value of Modified Qianjin Weijing Decoction in Acute Exacerbation of Chronic Obstructive Pulmonary Disease. *Diabetes World*, 17(4), 63 - 64.

116．Zhang, H. L. (2023). Influence of Xiaoqinglong Decoction on Pulmonary Function and Inflammatory Response in Patients with Acute Exacerbation of Chronic Obstructive Pulmonary Disease. *Acta Medicinae Sinica*, 36(03), 153 - 157. doi: 10.19296/j.cnki.1008 - 2409.2023 - 03 - 031

117．Zhang, L. H., Wu, J. J. (2011). Suzi Jiangqi Decoction Combined with Western Medicine in the Treatment of 40 Cases of Acute Attack of Chronic Obstructive Pulmonary Disease. *Chinese Medicine Modern Distance Education of China*, 9(15), 48 - 49.

118．Zhang, L. S. (2011). Efficacy Observation of Modified Weijing Decoction in the Treatment of Exacerbation of Chronic Obstructive Pulmonary Disease. *Journal of Shandong University of Traditional Chinese Medicine*, 35(02), 148 - 149. doi: 10.16294/j.cnki.1007 - 659x.2011.02.015

119．Zhang, M. C., Du, S. B., Fan, L. (2022). Clinical Efficacy of Xiaoqinglong Decoction Combined with Routine Treatment in Patients with Acute Phase of Chronic Obstructive Pulmonary Disease of External Cold and Internal Fluid - Retention Type. *Chinese Traditional Patent Medicine*, 44(01), 78 - 82.

120．Zhang, W. Z., Wu, S. S., Yan, Z. (2014). Clinical Observation of Integrated Traditional Chinese and Western Medicine in the Treatment of Acute Exacerbation of Elderly Chronic Obstructive Pulmonary Disease. *Journal of Emergency in Traditional Chinese Medicine*, 23(11), 2068 - 2070.

121．Zhang, Y. (2020). Influence of Modified Xiaoqinglong Decoction on Pulmonary Function and Laboratory Indicators in Patients with Acute Attack of Chronic Obstructive Pulmonary Disease. *Modern Journal of Integrated Traditional Chinese and Western Medicine*, 29(12), 1323 - 1326.

122．Zhao, D. L., Huang, Y., Li, F. L., Jiao, L., He, Y. Z., Jiang, Y. L., et al. (2020). Influence of Modified Qingqi Huatan Decoction on Serum MCP - 1 and sTREM - 1 Levels in Patients with Acute Exacerbation of Chronic Obstructive Pulmonary Disease. *World Journal of Integrated Traditional and Western Medicine*, 15(11), 2125 - 2129. doi: 10.13935/j.cnki.sjzx.201134

123．Zheng, X., Qu, N. N., Ma, L. J., Qin, Y. B., Xu, X. (2018). Influence of Suzi Jiangqi Decoction on Pulmonary Function and Partial Mechanisms in the Acute Attack Stage of Chronic Obstructive Pulmonary Disease (Phlegm - turbidity Obstructing the Lung Syndrome). *World Chinese Medicine*, 13(06), 1487 - 1491.

124．Zhu, C. W., Xiao, H. J. (2023). Clinical Observation of Xiaoqinglong Decoction in the Treatment of Elderly Patients with Chronic Obstructive Pulmonary Disease during the Attack Stage. *Guangming Journal of Chinese Medicine*, 38(06), 1078 - 1081.

125．Zhu, Y. L. (2019). Efficacy Observation of Modified Xiaoqinglong Decoction Combined with Tiotropium Bromide Powder for Inhalation in the Treatment of Acute Exacerbation of COPD. *Practical Clinical Journal of Integrated Traditional Chinese and Western Medicine*, 19(10), 12 - 14. doi: 10.13638/j.issn.1671 - 4040.2019.10.006

126．Zhuang, Y., Wu, L. C., Liu, M. (2020). Efficacy Analysis of Sangbaipi Decoction Combined with Western Medical Therapy in the Treatment of Chronic Obstructive Pulmonary Disease with Phlegm - heat Stagnating in the Lung Type. *Healthmust - Readmagazine*, (20), 176.

127．Zou, T. E., Wu, J. F., Qian, R. J., Liang, Y. (2021). Treatment of 60 Cases of Acute Exacerbation of Chronic Obstructive Pulmonary Disease with Phlegm - heat Accumulating in the Lung Type by Modified Maxing Shigan Decoction. *Zhejiang Journal of Traditional Chinese Medicine*, 56(07), 496. doi: 10.13633/j.cnki.zjtcm.2021.07.016

128．Zou, W. B., Fu, X. G., Li, M., Ruan, H., Liu, X., Zhang, Y. P. (2017). Clinical Efficacy and Immunomodulatory Effect of Xiaoqinglong Decoction Combined with Western Medicine in the Treatment of Elderly Patients with Chronic Obstructive Pulmonary Disease during the Attack Stage. *Chinese Journal of Gerontology*, 37(03), 638 - 639.

129．Han, W. L. (2025). Efficacy of Dingchuan Decoction Combined with Budesonide and Formoterol in the Treatment of Patients with Acute Exacerbation of Chronic Obstructive Pulmonary Disease. Chinese Journal of People's Health, 37(08), 109 - 111 + 115.

130．Lu, G. L., Gao, S. (2025). Study on Modified Xiaoqinglong Decoction Combined with Western Medicine in the Treatment of Patients with Acute Exacerbation of Chronic Obstructive Pulmonary Disease. Shenzhen Journal of Integrated Traditional Chinese and Western Medicine, 35(09), 46 - 49. doi: 10.16458/j.cnki.1007 - 0893.2025.09.013.

131．Le, X. (2025). Clinical Observation of Modified Suzi Jiangqi Decoction Combined with Montelukast Sodium in the Treatment of Acute Exacerbation of Chronic Obstructive Pulmonary Disease. Journal of Practical Traditional Chinese Medicine, 41(04), 798 - 800.

132．Qiu, Z. F. (2024). Clinical Observation of Modified Xiaoqinglong Decoction as Adjuvant Treatment for Acute Exacerbation of Chronic Obstructive Pulmonary Disease. Journal of Practical Traditional Chinese Medicine, 40(08), 1619 - 1622.

Appendix Table A.16: Herb details in included studies

Study ID	Formula name	Traditional Chinese medicine syndrome patterns	Herb ingredients and dosage[a] (g)	Usage	Quality control standards for drugs	Reported
Lin F 2024	DC	Phlegm and blood stasis obstructing the lung pattern	Bai Guo 9g, Ma Huang 9g, Ban Xia 9g, Kuan Dong Hua 9g, Sang Bai Pi 9g, Zi Su Zi 6g, Huang Qin 6g, Ku Xing Ren 4.5g, Gan Cao 3g	250/3 mL, three times per day (250 mL/dose)	Quality control standards for drugs in compliance with the Chinese Pharmacopoeia	Yes
Yao F 2021	DC	NR	Zi Wan 15g, Kuan Dong Hua 15g, Sang Bai Pi 15g, Zhi Mu 15g, Chen Pi 10g, Huang Qin 10g, Zi Su Zi 6g, Ku Xing Ren 6g, Zhe Bei Mu 6g, Ban Xia 6g, Gan Cao 3g	0.5 dose, twice per day	Quality control standards for drugs in compliance with the Chinese Pharmacopoeia	Yes
Zeng YZ 2024	DC	NR	Huang Qi 30g, Gan Cao 6g, Ma Huang 8g, Ku Xing Ren 9g, Zi Su Zi 9g, Sang Bai Pi 20g, Tai Zi Shen 15g, Huang Qin 15g, Zi Wan 15g, Kuan Dong Hua 15g, Zhi Mu 15g	150 mL, three times per day (450 mL/dose)	Quality control standards for drugs in compliance with the Chinese Pharmacopoeia	Yes
You WH 2022	DC	NR	Ma Huang 5g, Zi Su Zi 10g, Sang Bai Pi 10g, Ku Xing Ren 8g, Ban Xia 9g, Zhe Bei Mu 15g, Fu Ling 15g, Chen Pi 10g, Jie Geng 10g, Bai Bu 10g, Huang Qin 6g, Gan Cao 3g	0.25 dose, four times per day	Quality control standards for drugs in compliance with the Chinese Pharmacopoeia	Yes
Wu M 2021	DC	NR	Ma Huang 5g, Ku Xing Ren 5g, Ban Xia 5g, Gan Cao 5g, Bai Guo 10g, Zi Su Zi 10g, Sang Bai Pi 15g, Huang Qin 15g, Kuan Dong Hua 15g	0.5 dose, twice per day	Quality control standards for drugs in compliance with the Chinese Pharmacopoeia	Yes
Guan ZY 2021	ECSZYQ	NR	Ban Xia 15g, Jie Geng 15g, Fu Ling 15g, Chen Pi 12g, Gan Cao 10g, Ju Hong 10g, Lai Fu Zi 9g, Zi Su Zi 9g, Bai Jie Zi 9g	0.5 dose, twice per day	Quality control standards for drugs in compliance with the Chinese Pharmacopoeia	Yes
Xu H 2019	ECSZYQ	Phlegm dampness accumulating in the lung pattern	Zi Su Zi 10g, Bai Jie Zi 10g, Lai Fu Zi 10g, Ban Xia 10g, Fu Ling 10g, Wu Mei 10g, Gan Cao 6g, Sheng Jiang 6g	200 mL, twice per day (400 mL/dose)	Quality control standards for drugs in compliance with the Chinese Pharmacopoeia	Yes
Wei J 2022	ECSZYQ	Phlegm dampness accumulating in the lung pattern	Zi Su Zi 15g, Ban Xia 15g, Chen Pi 15g, Da Zao 15g, Bai Jie Zi 10g, Sheng Jiang 10g, Lai Fu Zi 30g, Gan Cao 5g, Fu Ling 20g	100 mL, three times per day (300 mL/dose)	Quality control standards for drugs in compliance with the Chinese Pharmacopoeia	Yes
Yang XL 2014	ECSZYQ	Phlegm dampness accumulating in the lung pattern	Chen Pi 10g, Ban Xia 10g, Fu Ling 10g, Bai Jie Zi 10g, Zi Su Zi 15g, Lai Fu Zi 15g, Dan Shen 15g, Gan Cao 6g	NR	Quality control standards for drugs in compliance with the Chinese Pharmacopoeia	Yes
Wei JQ 2023	ECSZYQ	NR	Ban Xia 10g, Ku Xing Ren 10g, Chen Pi 10g, Sheng Jiang 15g, Zi Su Zi 10g, Fu Ling 10g, Gui Zhi 15g, Bai Jie Zi 10g, Lai Fu Zi 10g, Cang Zhu 15g, Hou Po 10g, Gan Cao 5g	100 mL, twice per day (200 mL/dose)	Quality control standards for drugs in compliance with the Chinese Pharmacopoeia	Yes
Feng J 2023	ECSZYQ	NR	Jie Geng 15g, Ban Xia 15g, Fu Ling 15g, Zi Su Zi 9g, Bai Jie Zi 9g, Lai Fu Zi 9g, Ju Hong 10g	100 mL, twice per day (200 mL/dose)	Quality control standards for drugs in compliance with the Chinese Pharmacopoeia	Yes
Qi YX 2016	ECSZYQ	Phlegm dampness accumulating in the lung pattern	Ban Xia 15g, Chen Pi 12g, Ju Hong10g, Fu Ling 15g, Zi Su Zi 9g, Bai Jie Zi 9g, Lai Fu Zi 9g, Jie Geng 15g, Gan Cao 10g	0.5 dose, twice per day	Quality control standards for drugs in compliance with the Chinese Pharmacopoeia	Yes
Yan LW 2020	ECSZYQ	Phlegm dampness accumulating in the lung pattern	Zi Su Zi 9g, Bai Jie Zi 9g, Lai Fu Zi 9g, Ban Xia 15g, Ju Hong 10g, Fu Ling 15g, Gan Cao 10g	0.5 dose, twice per day	Quality control standards for drugs in compliance with the Chinese Pharmacopoeia	Yes
Fan PC 2020	ECSZYQ	NR	Ban Xia 15g, Jie Geng 15g, Fu Ling 15g, Chen Pi 12g, Ju Hong, Gan Cao 10g, Lai Fu Zi 9g, Bai Jie Zi 9g, Zi Su Zi 9g	0.5 dose, twice per day	Quality control standards for drugs in compliance with the Chinese Pharmacopoeia	Yes
Wang XB 2020	ECSZYQ	NR	Ban Xia 15g, Chen Pi 12g, Lai Fu Zi 9g, Gan Cao 10g, Jie Geng 15g, Bai Jie Zi 9g, Fu Ling 15g, Zi Su Zi 9g, Ju Hong 10g	0.5 dose, twice per day	Quality control standards for drugs in compliance with the Chinese Pharmacopoeia	Yes
Bai YL 2018	WJ	NR	Wei Jing 24g, Huang Qin 15g, Yi Yi Ren 30g, Tao Ren 12g, Dong Gua Pi 30g, Ku Xing Ren 12g, Bai Bu 15g, Kuan Dong Hua 15g, Ban Xia 12g, Gan Cao 6g	150 mL, twice per day (300 mL/dose)	Quality control standards for drugs in compliance with the Chinese Pharmacopoeia	Yes
Wang HX 2023	XQL	Exterior cold with interior fluid retention pattern	Ma Huang 20g, Ban Xia 15g, Gui Zhi 10g, Gan Cao 9g, Wu Wei Zi 9g, Bai Shao 10g, Jing Jie 10g, Kuan Dong Hua 10g, Jiang Can 10g, Di Long 10g, Xi Xin 6g, Gan Jiang 6g	150 mL, twice per day (300 mL/dose)	Quality control standards for drugs in compliance with the Chinese Pharmacopoeia	Yes
Xing HZ 2020	ECSZYQ	NR	Lai Fu Zi 10g, Zi Su Zi 10g, Bai Jie Zi 10g, Sheng Jiang 6g, Gan Cao 6g, Ban Xia 10g, Wu Mei 10g, Fu Ling 10g	200 mL, twice per day (400 mL/dose)	Quality control standards for drugs in compliance with the Chinese Pharmacopoeia	Yes
Ma JQ 2022	XQL	Exterior cold with interior fluid retention pattern	Ma Huang 9g, Bai Shao 9g, Wu Wei Zi 9g, Ban Xia 10g, Gui Zhi 10g, Huang Qin 10g, Chen Pi 10g, Kuan Dong Hua 10g, Zi Wan 10g, Ku Xing Ren 10g, Zi Su Zi 10g, Gan Jiang 6g, Gan Cao 6g, Xi Xin 3g	0.5 dose, twice per day	Quality control standards for drugs in compliance with the Chinese Pharmacopoeia	Yes

Wu JN 2016	DC	Qi deficiency, phlegm-heat and blood stasis pattern	Huang Qi 30g, Sang Bai Pi 20g, Huang Qin 15g, Zhi Mu 15g, Zi Wan 15g, Kuan Dong Hua 15g, Tai Zi Shen 15g, Zi Su Zi 9g, Ku Xing Ren 9g, Ma Huang 8g, Gan Cao 6g	200 mL, twice per day (400 mL/dose)	Quality control standards for drugs in compliance with the Chinese Pharmacopoeia	Yes
Yan HF 2020	DC	Phlegm heat accumulating in the lung pattern	Huang Qi 20g, Kuan Dong Hua 15g, Zhi Mu 15g, Huang Qin 15g, Sang Bai Pi 15g, Zi Wan 15g, Tai Zi Shen 15g, Ma Huang 10g, Di Long 10g, Ku Xing Ren 10g, Zi Su Zi 10g, Gan Cao 5g	150 mL, twice per day (300 mL/dose)	Quality control standards for drugs in compliance with the Chinese Pharmacopoeia	Yes
Wang W 2021	MXSG	Phlegm heat accumulating in the lung pattern	Zhe Bei Mu 10g, Shi Gao 15g, Ting Li Zi 10g, Ma Huang 6g, Zi Su Zi 10g, Ku Xing Ren 10g, Lai Fu Zi 10g, Sang Bai Pi 15g,	150 mL, twice per day (300 mL/dose)	Quality control standards for drugs in compliance with the Chinese Pharmacopoeia	Yes
Xu XP 2022	MXSG	Phlegm heat accumulating in the lung pattern	Shi Gao 20g, Yu Xing Cao 20g, Huang Qin 10g, Ting Li Zi 10g, Ma Huang 5g, Ku Xing Ren 10g, Gua Lou 15g, Qian Hu 10g, Zhe Bei Mu 10g, Gan Cao 10g	150 mL, twice per day (300 mL/dose)	Quality control standards for drugs in compliance with the Chinese Pharmacopoeia	Yes
Zou TE 2021	MXSG	Phlegm heat accumulating in the lung pattern	Shi Gao 30g, Sang Bai Pi 20g, Gua Lou 15g, Mai Dong 15g, Chen Pi 15g, Ku Xing Ren 10g, Huang Qin 10g, Bei Mu 10g, Ma Huang 6g, Gan Cao 6g	1 dose/day, once per day	Quality control standards for drugs in compliance with the Chinese Pharmacopoeia	Yes
Yue YX 2020	WJ	NR	Tao Ren 30g, Wei Jing 30g, Zhe Bei Mu 15g, Huang Qin 30g, Yi Yi Ren 30g, Gua Lou 15g, Dong Gua Ren 20g, Jie Geng 12g	50 mL, twice per day (100 mL/dose)	Quality control standards for drugs in compliance with the Chinese Pharmacopoeia	Yes
Mei Y 2022	WJ	Phlegm heat accumulating in the lung pattern	Wei Jing 30g, Tao Ren 30g, Dong Gua Ren 20g, Huang Qin 9g, Zhe Bei Mu 15g, Gua Lou 15g, Jie Geng 12g	100 mL, twice per day (200 mL/dose)	Quality control standards for drugs in compliance with the Chinese Pharmacopoeia	Yes
Niu J 2023	SBP	NR	Sang Bai Pi 20g, Shi Gao 15g, Bei Sha Shen 12g, Zi Su Zi 10g, Huang Qin 10g, Zi Wan 10g, Zhe Bei Mu 10g, Huang Lian 10g, Zhi Zi 10g, Gan Cao 10g, Yu Xing Cao 9g, Bai Bu 9g, Ban Xia 9g, Ku Xing Ren 6g	0.5 dose, twice per day	Quality control standards for drugs in compliance with the Chinese Pharmacopoeia	Yes
Bai FR 2024	SZJQ	Phlegm dampness accumulating in the lung pattern	Zi Su Zi 9g, Chen Pi 9g, Ban Xia 9g, Qian Hu 6g, Dang Gui 6g, Gan Cao 6g, Hou Po 6g, Rou Gui 3g, Sheng Jiang 3g, Da Zao 3g, Zi Su Ye 3g	150 mL, twice per day (300 mL/dose)	Quality control standards for drugs in compliance with the Chinese Pharmacopoeia	Yes
Jiang LX 2021	SZJQ	Phlegm dampness accumulating in the lung pattern	Zi Su Zi12g, Qian Hu 12g, Chen Pi 12g, Dan Nan Xing 12g, Dang Gui 12g, Ban Xia 10g, Hou Po 10g, Zi Su Ye 10g, Ku Xing Ren 10g, Sheng Jiang 10g, Da Zao 10g, Bai Zhu 15g, Rou Gui 5g, Ma Huang 8g	1/3 dose, three times per day	Quality control standards for drugs in compliance with the Chinese Pharmacopoeia	Yes
Su ZX 2019	SZJQ	Phlegm dampness accumulating in the lung pattern	Zi Su Zi 15g, Ban Xia 15g, Hou Po 15g, Chen Pi 15g, Zhi Qiao 15g, Bai Zhu 15g, Gua Lou Pi 15g, Dang Gui 10g, Qian Hu 10g, Rou Gui 10g, Sheng Jiang 10g, Chi Shao10g, Gan Cao 10g, Fu Ling 20g	100 mL, three times per day (300 mL/dose)	Quality control standards for drugs in compliance with the Chinese Pharmacopoeia	Yes
Feng J 2018	WJ	Phlegm heat accumulating in the lung pattern	Wei Jing 30g, Tao Ren 30g, Yi Yi Ren 30g, Huang Qin 30g, Dong Gua Ren 20g, Bei Mu 15g, Gua Lou 15g, Jie Geng 15g	150 mL, once per day (150 mL/dose)	Quality control standards for drugs in compliance with the Chinese Pharmacopoeia	Yes
Pan XD 2018	XQL	Exterior cold with interior fluid retention pattern	Ma Huang 10g, Jiang Can 10g, Di Long 10g, Ting Li Zi 10g, Bai Zhu 10g, Ban Xia 10g, Wu Wei Zi 5g, Gan Jiang 5g, Gui Zhi 5g, Xi Xin 5g, Gan Cao 5g	50 mL, twice per day (100 mL/dose)	Quality control standards for drugs in compliance with the Chinese Pharmacopoeia	Yes
Chen WC 2024	XBCQ	Phlegm heat accumulating in the lung pattern	Ting Li Zi 30g, Shi Gao 30g, Da Huang 10g, Gua Lou Pi 15g, Ku Xing Ren 15g	0.5 dose, twice per day	Quality control standards for drugs in compliance with the Chinese Pharmacopoeia	Yes
Huang F 2012	MXSG	Phlegm heat accumulating in the lung pattern	Ma Huang 5g, Ku Xing Ren 9g, Gan Cao 6g, Shi Gao18g	1/3 dose, three times per day	Quality control standards for drugs in compliance with the Chinese Pharmacopoeia	Yes
Lan H 2019	MXSG	NR	Tao Ren 12g, Zi Su Zi 12g, Gua Lou 12g, Da Huang 12g, Yu Xing Cao 15g, Ku Xing Ren 15g, Ma Huang 9g, Ban Xia 9g, Gan Cao 9g, Shi Gao 20g	200 mL, twice per day (400 mL/dose)	Quality control standards for drugs in compliance with the Chinese Pharmacopoeia	Yes
Wang ZF 2017	MXSG	NR	Shi Gao 20g, Ku Xing Ren 15g, Ma Huang 9g, Gan Cao 9g	100 mL, twice per day (200 mL/dose)	Quality control standards for drugs in compliance with the Chinese Pharmacopoeia	Yes
Xiong DN 2016	MXSG	NR	Ma Huang 10g, Ku Xing Ren 10g, She Gan 15g, Zhi Zi Wan 15g, Zhi Qiao 15g, Jie Geng 15g	200 mL, twice per day (200 mL/dose)	Quality control standards for drugs in compliance with the Chinese Pharmacopoeia	Yes
Sun HJ 2017	MXSG	NR	Shi Gao 20g, Ku Xing Ren 15g, Ma Huang 9g, Gan Cao 9g	100 mL, twice per day (200 mL/dose)	Quality control standards for drugs in compliance with the Chinese Pharmacopoeia	Yes
Gong Y 2022	MXSG	Phlegm heat accumulating in the lung pattern	Shi Gao 15g, Sang Bai Pi 15g, Chuan Xiong 6g, Ma Huang 6g, Zhe Bei Mu 10g, Lai Fu Zi 10g, Ku Xing Ren 10g, Ting Li Zi 10g	280 mL/day (280 mL/dose)	Quality control standards for drugs in compliance with the Chinese Pharmacopoeia	Yes
He CN 2017	MXSG	NR	Yu Xing Cao 15g, Ku Xing Ren 15g, Shi Gao20g, Sang Bai Pi 12g, Tao Ren 12g, Da Huang 12g, Gua Lou 12g, Gan Cao 9g, Ban Xia 9g, Ma Huang 9g	0.5 dose, twice per day	Quality control standards for drugs in compliance with the Chinese Pharmacopoeia	Yes
Gao ZT 2022	MXSG	NR	Gan Cao 9g, Ma Huang 9g, Ban Xia 9g, Zi Su Zi 12g, Da Huang 12g, Gua Lou 12g, Sang Bai Pi 12g, Ku Xing Ren 15g, Yu Xing Cao 15g, Shi Gao 20g	0.5 dose, twice per day	Quality control standards for drugs in compliance with the Chinese Pharmacopoeia	Yes

Lin CB 2018	MXSG	NR	Ma Huang 9g, Ban Xia 9g, Gan Cao9g, Shi Gao20g, Da Huang 12g, Sang Bai Pi 12g, Gua Lou 12g, Zi Su Zi 12g, Yu Xing Cao 15g, Ku Xing Ren 15g	100 mL, twice per day (200 mL/dose)	Quality control standards for drugs in compliance with the Chinese Pharmacopoeia	Yes
Sun XS 2015	MXSG	NR	Ma Huang 9g, Shi Gao 20g, Ku Xing Ren 15g, Da Huang 12g, Yu Xing Cao 15g, Sang Bai Pi 12g, Gua Lou 12g, Ban Xia 9g, Zi Su Zi 12g, Gan Cao 9g	100 mL, twice per day (200 mL/dose)	Quality control standards for drugs in compliance with the Chinese Pharmacopoeia	Yes
Duan WY 2021	MXSG	NR	Shi Gao 20g, Ku Xing Ren 15g, Yu Xing Cao 15g, Sang Bai Pi 12g, Gua Lou 12g, Dang Gui 12g, Zi Su Zi 12g, Da Huang 12g, Ban Xia 9g, Gan Cao 9g	100 mL, twice per day (200 mL/dose)	Quality control standards for drugs in compliance with the Chinese Pharmacopoeia	Yes
Liao Q 2020	MXSG	NR	Gan Cao 9g, Ban Xia 9g, Ma Huang 9g, Zi Su Zi 12g, Dang Gui 12g, Tao Ren 12g, Sang Bai Pi 12g, Gua Lou 12g, Ku Xing Ren 15g, Shi Gao 20g	100 mL, twice per day (200 mL/dose)	Quality control standards for drugs in compliance with the Chinese Pharmacopoeia	Yes
Xie WH 2009	MXSG	NR	Ma Huang 9g, Ku Xing Ren 10g, Shi Gao 20g, Zi Su Zi 10g, Di Long 12g, Huang Qin 12g, Dan Nan Xing 6g, Lian Qiao 15g, Ban Xia 10g, Gan Cao 10g	1 dose/day	Quality control standards for drugs in compliance with the Chinese Pharmacopoeia	Yes
Yan SL 2019	MXSG	NR	Yu Xing Cao 20g, Shi Gao 20g, Di Long 10g, Sang Bai Pi 10g, Ku Xing Ren 10g, Dang Gui 10g, Gua Lou 10g, Tao Ren 10g, Gan Cao6g, Ma Huang 4g	0.5 dose, twice per day	Quality control standards for drugs in compliance with the Chinese Pharmacopoeia	Yes
Song ZC 2020	MXSG	Exterior cold with lung heat pattern	Ku Xing Ren 15g, Sang Bai Pi 15g, Gui Zhi 6g, Yu Xing Cao 5g, Jin Yin Hua 20g, Kuan Dong Hua 15g, Shi Gao 15g, Chuan Bei Mu 10g, Ma Huang 3g, Gan Cao 3g	0.5 dose, twice per day	Quality control standards for drugs in compliance with the Chinese Pharmacopoeia	Yes
Wang XP 2015	MXSG	Exterior cold with lung heat pattern	Ma Huang, Ku Xing Ren, Shi Gao, Gan Cao (dosage not specified)	100 mL, twice per day (200 mL/dose)	Quality control standards for drugs in compliance with the Chinese Pharmacopoeia	Yes
Wang XD 2017	MXSG	NR	Ma Huang 6g, Shi Gao 30g, Ku Xing Ren 15g, Gan Cao 10g	200 mL, twice per day (400 mL/dose)	Quality control standards for drugs in compliance with the Chinese Pharmacopoeia	Yes
Liu YF 2023	ECSZYQ	Phlegm dampness accumulating in the lung pattern	Chen Pi 10g, Zi Su Zi 10g, Lai Fu Zi 10g, Ju Hong 10g, Jie Geng 10g, Ban Xia 9g, Gan Cao 9g, Fu Ling 15g, Bai Jie Zi 6g	0.5 dose, twice per day	Quality control standards for drugs in compliance with the Chinese Pharmacopoeia	Yes
Zhang Y 2023	ECSZYQ	NR	Lai Fu Zi 30g, Fu Ling 20g, Da Zao 15g, Ban Xia 15g, Chen Pi 15g, Zi Su Zi 15g, Sheng Jiang 10g, Bai Jie Zi 10g, Gan Cao 5g	100 mL, three times per day (300 mL/dose)	Quality control standards for drugs in compliance with the Chinese Pharmacopoeia	Yes
Hao SR 2018	WJ	NR	Wei Jing 20g, Dong Gua Ren 15g, Tao Ren 20g, Fu Ling 15g, Chen Pi 15g, Yi Yi Ren 15g	125 mL, twice per day (250 mL/dose)	Quality control standards for drugs in compliance with the Chinese Pharmacopoeia	Yes
Dai YM 2013	WJ	Phlegm heat accumulating in the lung pattern	Wei Jing 30g, Yi Yi Ren 30g, Huang Qin 30g, Tao Ren 30g, Dong Gua Ren 20g, Jie Geng 12g, Zhe Bei Mu 15g, Gua Lou 15g	100 mL, twice per day (200 mL/dose)	Quality control standards for drugs in compliance with the Chinese Pharmacopoeia	Yes
Ma ZH 2020	QQHT	NR	Gua Lou 20g, Fu Ling 15g, Huang Qin 10g, Zhi Shi 10g, Dan Nan Xing 9g, Ban Xia 9g, Ku Xing Ren 9g, Chen Pi 9g, Sheng Jiang 6g	150 mL, twice per day (300 mL/dose)	Quality control standards for drugs in compliance with the Chinese Pharmacopoeia	Yes
Yao B 2023	QQHT	Phlegm heat accumulating in the lung pattern	Gua Lou 20g, Fu Ling 15g, Huang Qin 10g, Zhi Shi 10g, Ku Xing Ren 9g, Ban Xia 9g, Dan Nan Xing 9g, Chen Pi 9g, Sheng Jiang 6g	150 mL, twice per day (300 mL/dose)	Quality control standards for drugs in compliance with the Chinese Pharmacopoeia	Yes
Xie GB 2023	QQHT	Phlegm heat accumulating in the lung pattern	Zhi Shi 10g, Chen Pi 10g, Huang Qin 15g, Ku Xing Ren 10g, Fu Ling 20g, Gua Lou Ren 15g, Dan Nan Xing 10g, Ban Xia 10g, Sheng Jiang (3 slices)	200 mL, twice per day (400 mL/dose)	Quality control standards for drugs in compliance with the Chinese Pharmacopoeia	Yes
Zhao DL 2020	QQHT	Phlegm heat accumulating in the lung pattern	Dan Nan Xing 15g, Gua Lou 15g, Ban Xia 9g, Zhe Bei Mu 9g, Zhi Shi 9g, Sang Bai Pi 12g, Huang Qin 9g, Ku Xing Ren 9g, Bai Tou Weng 12g, Yu Xing Cao 18g, Fu Ling 12g, Chen Pi 9g	200 mL, twice per day (400 mL/dose)	Quality control standards for drugs in compliance with the Chinese Pharmacopoeia	Yes
Kou ZJ 2024	QQHT	Phlegm heat accumulating in the lung pattern	Gua Lou 20g, Huang Qin 10g, Fu Ling 15g, Zhi Shi 10g, Chen Pi 9g, Ban Xia 9g, Dan Nan Xing 9g, Ku Xing Ren 9g, Sheng Jiang 6g	150 mL, twice per day (300 mL/dose)	Quality control standards for drugs in compliance with the Chinese Pharmacopoeia	Yes
Su W 2020	QQHT	Phlegm heat accumulating in the lung pattern	Huang Qin 9g, Dan Nan Xing 9g, Jie Geng 9g, Ban Xia 9g, Chen Pi 9g, Fu Ling 12g, Gan Cao 9g, Ku Xing Ren 9g, Gua Lou 12g, Zhi Shi 9g, Zi Su Zi 12g, Zhi Pi Pa Ye 12g, Dong Gua Ren 6g, Chuan Bei Mu 9g	0.5 dose, twice per day	Quality control standards for drugs in compliance with the Chinese Pharmacopoeia	Yes
Lv T 2014	QQHT	NR	Chen Pi 6g, Ku Xing Ren 6g, Zhi Shi 6g, Huang Qin 6g, Gua Lou Ren 6g, Fu Ling 6g, Dan Nan Xing 9g, Ban Xia 9g	1 dose/day	Quality control standards for drugs in compliance with the Chinese Pharmacopoeia	Yes
Wang XH 2023	QQHT	Phlegm heat accumulating in the lung pattern	Huang Qin 10g, Sang Bai Pi 12g, Ku Xing Ren 9g, Jie Geng 9g, Gua Lou Pi 20g, Zhe Bei Mu 9g, Ban Xia 9g	150 mL, twice per day (300 mL/dose)	Quality control standards for drugs in compliance with the Chinese Pharmacopoeia	Yes
Qian JY 2019	ECSZYQ	NR	Lai Fu Zi 15g, Zi Su Zi 15g, Bai Jie Zi 15g, Ju Hong 15g, Fu Ling 15g, Pi Pa Ye 15g, Chen Pi 12g, Ban Xia 12g, Jie Geng 12g, Bai Bu 10g, Qian Hu 10g, Ku Xing Ren 9g, Gan Cao 6g	150 mL, twice per day (300 mL/dose)	Quality control standards for drugs in compliance with the Chinese Pharmacopoeia	Yes

Yu XD 2022	ECSZYQ	NR	Fu Ling 15g, Chen Pi 12g, Gan Cao 10g, Zi Su Zi 9g, Lai Fu Zi 9g, Bai Jie Zi 10g, Ban Xia 15g, Ju Hong 10g, Jie Geng 15g	200 mL, twice per day (400 mL/dose)	Quality control standards for drugs in compliance with the Chinese Pharmacopoeia	Yes
Tang XJ 2023	SBP	Phlegm heat accumulating in the lung pattern	Sang Bai Pi 10g, Zi Su Zi 10g, Ku Xing Ren 10g, Huang Qin 10g, Jie Geng 10g, Zhe Bei Mu 10g, Di Long 10g, Gua Lou Pi 15g, Ban Xia 9g, Gan Cao 10g	150 mL, twice per day (300 mL/dose)	Quality control standards for drugs in compliance with the Chinese Pharmacopoeia	Yes
Liu GJ 2024	SBP	Phlegm heat accumulating in the lung pattern	Sang Bai Pi 15g, Jin Yin Hua 15g, Yu Xing Cao 15g, Huang Qin 12g, Zhe Bei Mu 12g, Jie Geng 12g, Gua Lou Pi 12g, Hong Jing Tian 12g, Ban Xia 10g, Zi Su Zi 10g, Ku Xing Ren 10g, Zhi Zi 10g, Di Long 10g, Gan Cao 10g	200 mL, twice per day (400 mL/dose)	Quality control standards for drugs in compliance with the Chinese Pharmacopoeia	Yes
Liu YC 2022	SBP	Phlegm heat accumulating in the lung pattern	Sang Bai Pi 30g, Ban Xia 15g, Zhe Bei Mu 15g, Bai Shao 15g, Ku Xing Ren 12g, Zi Su Zi 10g, Jie Geng 10g, Huang Qin 10g, Chen Pi 10g, Zhi Zi 10g, Gan Cao 10g	150 mL, twice per day (300 mL/dose)	Quality control standards for drugs in compliance with the Chinese Pharmacopoeia	Yes
Gao BJ 2022	SBP	NR	Gan Cao 6g, Ma Huang 10g, Zi Su Zi 10g, Ku Xing Ren 10g, Huang Qin 10g, Ban Xia 10g, Sang Bai Pi 15g, Zhe Bei Mu 15g	0.5 dose, twice per day	Quality control standards for drugs in compliance with the Chinese Pharmacopoeia	Yes
Liang ZX 2023	SBP	NR	Sang Bai Pi 30g, Ban Xia 15g, Huang Lian 3g, Bei Mu 15g, Huang Qin 10g, Ku Xing Ren 12g, Zi Su Zi 10g, Zhi Zi 10g	0.5 dose, twice per day	Quality control standards for drugs in compliance with the Chinese Pharmacopoeia	Yes
Chen L 2018	SBP	Phlegm heat accumulating in the lung pattern	Sang Bai Pi 12g, Zhe Bei Mu 12g, Huang Qin 12g, Ku Xing Ren 10g, Ban Xia 10g, Zi Su Zi 10g, Huang Lian 3g, Zhi Zi 6g	200 mL, twice per day (400 mL/dose)	Quality control standards for drugs in compliance with the Chinese Pharmacopoeia	Yes
Liu JJ 2021	SBP	Phlegm heat accumulating in the lung pattern	Sang Bai Pi 10g, Zhe Bei Mu 10g, Huang Qin 10g, Bai Qian 10g, Ma Huang 10g, Ku Xing Ren 15g, Ban Xia 9g, Bai Guo 15g, Gua Lou Pi 15g, Gan Cao 10g	1 dose/day	Quality control standards for drugs in compliance with the Chinese Pharmacopoeia	Yes
Fei X 2024	SBP	Phlegm heat accumulating in the lung pattern, stagnant blood obstructing the meridians pattern	Sang Bai Pi 18g, Ban Xia 10g, Zi Su Zi 10g, Ku Xing Ren 10g, Chuan Bei Mu 10g, Huang Qin 12g, Huang Lian 15g, Zhi Zi 10g	200 mL, twice per day (400 mL/dose)	Quality control standards for drugs in compliance with the Chinese Pharmacopoeia	Yes
Zheng X 2018	SZJQ	Phlegm dampness accumulating in the lung pattern	Zi Su Zi 15g, Ban Xia 12g, Qian Hu 12g, Hou Po 10g, Dang Gui 10g, Rou Gui 6g, Zi Wan 10g, Kuan Dong Hua 10g, Sheng Jiang 6g, Da Zao 10g, Gan Cao 6g	100 mL, three times per day (300 mL/dose)	Quality control standards for drugs in compliance with the Chinese Pharmacopoeia	Yes
Cai B 2018	SZJQ	Upper excess with lower deficiency pattern, root deficiency and branch excess	Zi Su Zi 9g, Ban Xia 9g, Qian Hu 6g, Gan Cao 6g, Hou Po 6g, Dang Gui 6g, Chen Pi 5g, Rou Gui 3g, Da Zao (1 piece), Sheng Jiang (2 slices)	50 mL, twice per day (100 mL/dose)	Quality control standards for drugs in compliance with the Chinese Pharmacopoeia	Yes
Yuan C 2021	SZJQ	Phlegm dampness accumulating in the lung pattern	Zi Su Zi 10g, Zi Su Ye 10g, Ban Xia 9g, Hou Po 6g, Qian Hu 10g, Dang Gui 10g, Rou Gui 6g, Sheng Jiang 6g, Da Zao 10g, Gan Cao 6g	100 mL, twice per day (200 mL/dose)	Quality control standards for drugs in compliance with the Chinese Pharmacopoeia	Yes
Chen DM 2020	SZJQ	Phlegm dampness accumulating in the lung pattern	Zi Su Zi 9g, Ban Xia 9g, Zi Su Ye 6g, Rou Gui 6g, Qian Hu 6g, Dang Gui 6g, Hou Po 6g, Gan Cao 6g, Da Zao (2 pieces), Sheng Jiang (2 slices)	150 mL, twice per day (300 mL/dose)	Quality control standards for drugs in compliance with the Chinese Pharmacopoeia	Yes
Li YH 2023	SZJQ	NR	Rou Gui 3g, Chen Pi 5g, Qian Hu 6g, Hou Po 6g, Dang Gui 6g, Gan Cao 6g, Ban Xia 9g, Zi Su Zi 9g Da Zao (1 piece), Sheng Jiang (2 slices)	100 mL, three times per day (300 mL/dose)	Quality control standards for drugs in compliance with the Chinese Pharmacopoeia	Yes
Jiang MH 2021	SZJQ	NR	Zi Su Zi 9g, Ban Xia 9g, Hou Po 6g, Chen Pi 6g, Qian Hu 6g, Dang Gui 6g, Gan Cao 6g, Rou Gui 3g, Da Zao (1 piece), Sheng Jiang (2 slices)	0.5 dose, twice per day	Quality control standards for drugs in compliance with the Chinese Pharmacopoeia	Yes
Yan HM 2020	SZJQ	Phlegm heat accumulating in the lung pattern	Zi Su Zi 9g, Ban Xia 9g, Qian Hu 6g, Hou Po 6g, Chen Pi 6g, Dang Gui 6g, Rou Gui 3g, Da Zao (1 piece), Sheng Jiang (2 slices), Gan Cao 6g	1 dose/day	Quality control standards for drugs in compliance with the Chinese Pharmacopoeia	Yes
Wang L 2017	SZJQ	NR	Zi Su Zi 9g, Ban Xia 9g, Gan Cao 6g, Qian Hu 6g, Hou Po 6g, Dang Gui 6g, Rou Gui 3g, Chen Pi 5g, Da Zao (1 piece), Sheng Jiang (2 slices)	0.5dose, twice per day	Quality control standards for drugs in compliance with the Chinese Pharmacopoeia	Yes
Jiang L 2018	SZJQ	Upper excess with lower deficiency pattern	Zi Su Zi 15g, Ban Xia 12g, Qian Hu 12g, Hou Po 10g, Dang Gui 10g, Rou Gui 6g, Zi Su Ye 6g, Sheng Jiang 6g, Gan Cao 6g	0.5 dose, twice per day	Quality control standards for drugs in compliance with the Chinese Pharmacopoeia	Yes
Chen GL 2024	SZJQ	NR	Zi Su Zi 9g, Hou Po 6g, Ban Xia 9g, Chen Pi 5g, Dang Gui 6g, Rou Gui 3g, Qian Hu 6g, Gan Cao 5g, Sheng Jiang 2g	150 mL, three times per day (300 mL/dose)	Quality control standards for drugs in compliance with the Chinese Pharmacopoeia	Yes
Wang LY 2022	SZJQ	NR	Chen Pi 6g, Gan Cao 6g, Qian Hu 6g, Dang Gui 6g, Rou Gui 6g, Ban Xia 9g, Zi Su Zi 9g, Da Zao (1 piece), Sheng Jiang (2 slices)	150 mL, twice per day (300 mL/dose)	Quality control standards for drugs in compliance with the Chinese Pharmacopoeia	Yes
Yang BJ 2018	SZJQ	Phlegm dampness accumulating in the lung pattern	Zi Su Zi 20g, Ban Xia 9g, Gan Cao 6g, Qian Hu 10g, Chen Pi 10g, Hou Po 10g, Rou Gui 3g, Dang Gui 10g, Quan Xie 5g, Di Long 12g, Da Zao (3 pieces), Sheng Jiang (3 slices)	200 mL, twice per day (400 mL/dose)	Quality control standards for drugs in compliance with the Chinese Pharmacopoeia	Yes
Liu JH 2024	SZJQ	NR	Zi Su Zi 10g, Ban Xia 9g, Zi Su Ye 10g, Qian Hu 10g, Dang Gui 10g, Sheng Jiang 6g, Da Zao 10g, Hou Po 6g, Rou Gui 6g, Gan Cao 6g	100 mL, twice per day (200 mL/dose)	Quality control standards for drugs in compliance with the Chinese Pharmacopoeia	Yes
Mao ZX 2019	SZJQ	NR	Zi Su Zi 9g, Ban Xia 9g, Qian Hu 6g, Hou Po 6g, Dang Gui 6g, Gan Cao 6g, Chen Pi 3g, Dang Gui 3g, Da Zao (1 piece), Sheng Jiang (2 slices)	100 mL, twice per day (200 mL/dose)	Quality control standards for drugs in compliance with the Chinese Pharmacopoeia	Yes

Zhang LH 2011	SZJQ	NR	Zi Su Zi 12g, Ban Xia 9g, Hou Po 6g, Qian Hu 9g, Dang Gui 12g, Rou Gui 3g, Zi Su Ye 6g, Gan Cao 6g	200 mL, twice per day (400 mL/dose)	Quality control standards for drugs in compliance with the Chinese Pharmacopoeia	Yes
Dai LF 2023	SZJQ	NR	Gan Cao 6g, Hou Po 6g, Dang Gui 6g, Qian Hu 6g, Rou Gui 6g, Zi Su Ye 6g, Ban Xia 9g, Zi Su Zi 9g, Da Zao (2 pieces), Sheng Jiang (2 slices)	200 mL, twice per day (400 mL/dose)	Quality control standards for drugs in compliance with the Chinese Pharmacopoeia	Yes
Deng HX 2013	SZJQ	Upper excess with lower deficiency pattern, root deficiency and branch excess	Zi Su Zi, Da Zao, Rou Gui, Sheng Jiang, Ban Xia, Qian Hu, Hou Po, Dang Gui, Gan Cao (dosage not specified)	50 mL, twice per day (100 mL/dose)	Quality control standards for drugs in compliance with the Chinese Pharmacopoeia	Yes
Fu JY 2018	SZJQ	NR	Zi Su Zi 9g, Ban Xia 9g, Dang Gui 6g, Gan Cao 6g, Qian Hu 6g, Hou Po 6g, Rou Gui 6g, Da Zao (2 pieces), Sheng Jiang (2 slices)	150 mL, twice per day (300 mL/dose)	Quality control standards for drugs in compliance with the Chinese Pharmacopoeia	Yes
Yuan LD 2017	WJ	Phlegm heat accumulating in the lung pattern	Wei Jing 30g, Tao Ren 30g, Yi Yi Ren 30g, Huang Qin 30g, Dong Gua Ren 20g, Zhe Bei Mu 15g, Gua Lou 15g, Jie Geng 15g	150 mL, once per day (150 mL/dose)	Quality control standards for drugs in compliance with the Chinese Pharmacopoeia	Yes
Zhang LS 2011	WJ	Phlegm heat accumulating in the lung pattern	Wei Jing 30g, Dong Gua Ren 20g, Yi Yi Ren 30g, Tao Ren 30g, Huang Qin 30g, Gua Lou 15g, Zhe Bei Mu 15g, Jie Geng 12g	100 mL, twice per day (200 mL/dose)	Quality control standards for drugs in compliance with the Chinese Pharmacopoeia	Yes
Liu SN 2022	WJ	NR	Yi Yi Ren 30g, Dong Gua Ren 30g, Wei Jing 24g, Kuan Dong Hua 15g, Huang Qin 15g, Tao Ren 12g, Ku Xing Ren 12g, Ban Xia 12g, Gua Lou Ren 12g, Gan Cao 6g	150 mL, twice per day (300 mL/dose)	Quality control standards for drugs in compliance with the Chinese Pharmacopoeia	Yes
Chen XJ 2019	XQL	NR	Ma Huang 5g, Zi Su Zi 10g, Ban Xia 10g, She Gan 10g, Bai Shao 10g, Gui Zhi 9g, Ju Hong 9g, Dan Shen 9g, Bai Shao 12g, Gan Jiang 6g, Chen Pi 6g, Xi Xin 3g, Wu Wei Zi 5g, Jie Geng 5g	0.5 dose, twice per day	Quality control standards for drugs in compliance with the Chinese Pharmacopoeia	Yes
Zhang HL 2023	XQL	Exterior cold with interior fluid retention pattern	Ma Huang 10g, Shao Yao10g, Gan Jiang 10g, Gan Cao10g, Gui Zhi 10g, Ban Xia 10g, Xi Xin 6g, Wu Wei Zi 6g	100 mL, twice per day (200 mL/dose)	Quality control standards for drugs in compliance with the Chinese Pharmacopoeia	Yes
Wang S 2013	XQL	NR	Not specified	100 mL, three times per day (300 mL/dose)	Quality control standards for drugs in compliance with the Chinese Pharmacopoeia	Yes
Wu YW 2015	XQL	NR	Ma Huang 9g, Gui Zhi 9g, Xi Xin 3g, Gan Jiang 6g, Shao Yao 9g, Ban Xia 9g, Wu Wei Zi 6g, Gan Cao 6g	100 mL, three times per day (300 mL/dose)	Quality control standards for drugs in compliance with the Chinese Pharmacopoeia	Yes
Liu YX 2013	XQL	NR	Ma Huang 15g, Gui Zhi 15g, Ban Xia 15g, Xi Xin 3g, Gan Jiang 10g, Gan Cao 10g, Wu Wei Zi 10g	150 mL, twice per day (300 mL/dose)	Quality control standards for drugs in compliance with the Chinese Pharmacopoeia	Yes
Ren X 2021	XQL	Exterior cold with interior fluid retention pattern	Ma Huang 10g, Ban Xia 15g, Wu Wei Zi 10g, Ku Xing Ren 20g, Xi Xin 5g, Shao Yao 15g, Gan Cao 10g, Gan Jiang 10g, Gui Zhi 10g	100 mL, twice per day (200 mL/dose)	Quality control standards for drugs in compliance with the Chinese Pharmacopoeia	Yes
Chen R 2021	XQL	Exterior cold with interior fluid retention pattern	Ma Huang 20g, Ban Xia15g, Di Long10g, Kuan Dong Hua10g, Jing Jie 10g, Jiang Can 10g, Wu Wei Zi 10g, Gui Zhi 6g, Ting Li Zi 6g, Gan Jiang 6g, Xi Xin 3g, Gan Cao 3g	150 mL, twice per day (300 mL/dose)	Quality control standards for drugs in compliance with the Chinese Pharmacopoeia	Yes
Zhu YL 2019	XQL	Exterior cold with interior fluid retention pattern	Ma Huang 10g, Gan Jiang 10g, Xi Xin 10g, Wu Wei Zi 15g, Shao Yao 5g, Ban Xia 5g, Gui Zhi 10g, Gan Cao 20g	100 mL, twice per day (200 mL/dose)	Quality control standards for drugs in compliance with the Chinese Pharmacopoeia	Yes
Han ZQ 2014	XQL	NR	Ban Xia 15g, Ma Huang 10g, Bai Jie Zi 15g, Gan Cao 10g, Ku Xing Ren 15g, Dang Shen 15g, Huang Qi 30g, Xi Xin 3g	100 mL, twice per day (200 mL/dose)	Quality control standards for drugs in compliance with the Chinese Pharmacopoeia	Yes
Tan YL 2017	XQL	NR	Ban Xia 15g, Bai Jie Zi 15g, Ku Xing Ren 15g, Dang Shen 15g, Ma Huang10g, Gan Cao10g, Huang Qi 30g, Xi Xin 3g	100 mL, twice per day (200 mL/dose)	Quality control standards for drugs in compliance with the Chinese Pharmacopoeia	Yes
Wang HM 2023	XQL	Exterior cold with interior fluid retention pattern	Ma Huang 20g, Ban Xia 12g, Bai Shao 10g, Gui Zhi 10g, Wu Wei Zi 9g, Gan Jiang 8g, Gan Cao 6g, Xi Xin 3g	200 mL, twice per day (400 mL/dose)	Quality control standards for drugs in compliance with the Chinese Pharmacopoeia	Yes
Zhang Y 2020	XQL	Exterior cold with interior fluid retention pattern	Ma Huang 9g, Ban Xia 10g, Gui Zhi 15g, Gan Jiang 10g, Wu Wei Zi 10g, Bai Shao 20g, Qiang Huo 10g, Xin Yi 8g, Xi Xin 3g, Di Long 6g, Du Huo 10g, Gan Cao 6g	150 mL, twice per day (300 mL/dose)	Quality control standards for drugs in compliance with the Chinese Pharmacopoeia	Yes
Ni XQ 2024	XQL	Phlegm dampness accumulating in the lung pattern	Ma Huang 9g, Gui Zhi 18g, Gan Jiang 12g, Xi Xin 9g, Ban Xia 9g, Bai Shao 15g, Wu Wei Zi 15g, Wu Yao 9g, Gan Cao 9g, Dang Shen 30g, Chen Pi 15g, Ku Xing Ren 15g, Zhi Qiao 15g, Ci Shi 15g	150 mL, twice per day (300 mL/dose)	Quality control standards for drugs in compliance with the Chinese Pharmacopoeia	Yes
Zhang MC 2022	XQL	Exterior cold with interior fluid retention pattern	Ma Huang 20g, Ban Xia 15g, Di Long 10g, Kuan Dong Hua 10g, Jing Jie 10g, Jiang Can 10g, Wu Wei Zi 10g, Gui Zhi 6g, Ting Li Zi 6g, Gan Jiang 6g, Xi Xin 3g, Gan Cao 3g	150 mL, twice per day (300 mL/dose)	Quality control standards for drugs in compliance with the Chinese Pharmacopoeia	Yes

Luo ZQ 2012	XQL	NR	Ma Huang 8g, Gui Zhi 8g, Gan Cao 8g, Ban Xia 10g, Wu Wei Zi 10g, Bai Shao 10g, Gan Jiang 10g, Xi Xin 5g	200 mL, three times per day (600 mL/dose)	Quality control standards for drugs in compliance with the Chinese Pharmacopoeia	Yes
Shi YJ 2015	XQL	NR	Ma Huang 15g, Bai Shao 15g, Gui Zhi 15g, Ban Xia 15g, Xi Xin 3g, Gan Jiang 10g, Gan Cao 10g, Wu Wei Zi 10g	150 mL, twice per day (300 mL/dose)	Quality control standards for drugs in compliance with the Chinese Pharmacopoeia	Yes
Zou WB 2017	XQL	NR	Ma Huang 5g, Zi Su Zi 10g, Gui Zhi 9g, Ban Xia 10g, Bai Shao 12g, Chen Pi 6g, She Gan 10g, Dan Shen 9g, Chao Bai Shao 10g, Gan Jiang 6g, Xi Xin 3g, Ju Hong 9g, Fu Ling 15g, Wu Wei Zi 5g	0.5 dose, twice per day	Quality control standards for drugs in compliance with the Chinese Pharmacopoeia	Yes
Li JL 2015	XQL	NR	Ma Huang 9g, Xi Xin 3g, Shao Yao 9g, Wu Wei Zi 6g, Gui Zhi 9g, Gan Jiang 6g, Ban Xia 9g, Gan Cao 6g	100 mL, twice per day (200 mL/dose)	Quality control standards for drugs in compliance with the Chinese Pharmacopoeia	Yes
Sun HW 2021	XQL	Exterior cold with interior fluid retention pattern	Ma Huang 9g, Gui Zhi 15g, Bai Shao 15g, Gan Jiang 10g, Wu Wei Zi 10g, Xin Yi 10g, Ban Xia 10g, Qiang Huo 10g, Du Huo 10g, Xi Xin 3g	200 mL, twice per day (400 mL/dose)	Quality control standards for drugs in compliance with the Chinese Pharmacopoeia	Yes
Zhu CW 2023	XQL	NR	Xi Xin 3g, Ma Huang 5g, Wu Wei Zi 5g, Jie Geng 5g, Chen Pi 6g, Gan Jiang 6g, Gui Zhi 9g, Dan Shen 9g, Ju Hong 9g, Zi Su Zi 10g, Ban Xia 10g, She Gan 10g, Bai Shao 12g, Fu Ling 15g	150 mL, twice per day (300 mL/dose)	Quality control standards for drugs in compliance with the Chinese Pharmacopoeia	Yes
Yu L 2015	XQL	Exterior cold with interior fluid retention pattern	Ma Huang 10g, Gui Zhi 15g, Gan Jiang 10g, Xi Xin 3g, Ban Xia 10g, Bai Shao 15g, Wu Wei Zi 15g, Gan Cao 15g, Zi Wan 15g, Bu Gu Zhi 15g, Zi Su Zi 15g	100 mL, three times per day (300 mL/dose)	Quality control standards for drugs in compliance with the Chinese Pharmacopoeia	Yes
Xu XM 2016	XQL	NR	Ma Huang 9g, Shao Yao 9g, Xi Xin 9g, Gui Zhi 9g, Wu Wei Zi 12g, Ban Xia 12g, Gan Jiang 9g, Huang Qin 9g, Fu Ling 9g, Ku Xing Ren 9g, Gan Cao 6g	250 mL, twice per day (500 mL/dose)	Quality control standards for drugs in compliance with the Chinese Pharmacopoeia	Yes
Chang QJ 2016	XBCQ	NR	Shi Gao 30g, Gua Lou 15g, Da Huang 10g, Ku Xing Ren 10g, Huang Qin 15g, Yu Xing Cao 15g, Zhe Bei Mu 15g, Ting Li Zi 15g, Lu Gen 25g, Gan Cao 15g	150 mL, twice per day (300 mL/dose)	Quality control standards for drugs in compliance with the Chinese Pharmacopoeia	Yes
Gu YY 2019	XBCQ	NR	Gua Lou 20g, Xie Bai 15g, Huang Qin 10g, Sang Bai Pi 15g, Zhi Zi Wan 15g, Kuan Dong Hua 15g, Ma Huang 10g, Ku Xing Ren 10g, Da Huang 5g, Dan Shen 15g, Gan Cao 6g	100 mL, twice per day (200 mL/dose)	Quality control standards for drugs in compliance with the Chinese Pharmacopoeia	Yes
Fu YX 2016	XBCQ	Phlegm heat accumulating in the lung pattern	Shi Gao, Da Huang, Ku Xing Ren, Gua Lou Pi (dosage not specified)	200 mL, twice per day (400 mL/dose)	Quality control standards for drugs in compliance with the Chinese Pharmacopoeia	Yes
Zhuang X 2020	SBP	Phlegm heat accumulating in the lung pattern	Sang Bai Pi 12g, Zhe Bei Mu 12g, Huang Qin 12g, Ku Xing Ren 10g, Ban Xia 10g, Zi Su Zi 10g, Shan Zhi 6g, Huang Lian 3g	150 mL, twice per day (300 mL/dose)	Quality control standards for drugs in compliance with the Chinese Pharmacopoeia	Yes
Ji XX 2020	YBBX	NR	Ma Huang 10g, Shi Gao 30g, Sheng Jiang 6g, Gan Cao 6g, Da Zao 12g, Ban Xia 15g	100 mL, three times per day (300 mL/dose)	Quality control standards for drugs in compliance with the Chinese Pharmacopoeia	Yes
Xiao LS 2021	YBBX	Phlegm heat accumulating in the lung pattern	Ma Huang 12g, Shi Gao 25g, Sheng Jiang 9g, Gan Cao 6g, Ban Xia 9g, Da Zao (15 pieces)	(100 mL/dose)	Quality control standards for drugs in compliance with the Chinese Pharmacopoeia	Yes
Wang PC 2012	YBBX	Phlegm heat accumulating in the lung pattern	First week: Ma Huang 10g, Shi Gao 30g, Sheng Jiang 6g, Da Zao 12g, Gan Cao 6g, Ban Xia 15g, Dan Shen 20g, Di Long 10g<br>Second week: Ma Huang 10g, Shi Gao 20g, Sheng Jiang 6g, Da Zao 12g, Gan Cao 6g, Ban Xia 10g, Dang Shen 15g, Bai Zhu 15g, Fu Ling 10g, Dan Shen 10g, Di Long 10g	1 dose/day	Quality control standards for drugs in compliance with the Chinese Pharmacopoeia	Yes
Guo WX 2007	XBCQ	Phlegm heat accumulating in the lung pattern	Shi Gao 15g, Huang Qin 15g, Gan Cao 6g, Ku Xing Ren 6g, Gua Lou 12g, Yu Xing Cao 30g, Da Huang 6g	25 mL, four times per day (100 mL/dose)	Quality control standards for drugs in compliance with the Chinese Pharmacopoeia	Yes
Liu H 2017	XQL	Cold fluids affecting the lung pattern	Ma Huang 9g, Gui Zhi 15g, Bai Shao 10g, Gan Cao 5g, Gan Jiang 10g, Xi Xin 3g, Ban Xia 12g, Wu Wei Zi 6g	200 mL, twice per day (400 mL/dose)	Quality control standards for drugs in compliance with the Chinese Pharmacopoeia	Yes
Zhang WZ 2014	SZJQ	Upper excess with lower deficiency pattern, root deficiency and branch excess	Zi Su Zi 15g, Ban Xia 10g, Hou Po 6g, Qian Hu 12g, Dang Gui 12g, Rou Gui 3g, Gan Cao 6g	200 mL, twice per day (400 mL/dose)	Quality control standards for drugs in compliance with the Chinese Pharmacopoeia	Yes
Wu CH 2015	WJ	Phlegm heat accumulating in the lung pattern	Wei Jing 20g, Yi Yi Ren 20g, Dong Gua Ren 15g, Tao Ren 15g, Gua Lou 15g, Dan Shen 15g, Yu Xing Cao 15g, Ban Xia 9g, Zhe Bei Mu 12g, Gan Cao 6g	150 mL, once per day (150 mL/dose)	Quality control standards for drugs in compliance with the Chinese Pharmacopoeia	Yes

Wang YX 2017	YBBX	NR	Chen Pi 15g, Huang Qin 15g, Gan Cao 10g, Zhe Bei Mu 10g, Ku Xing Ren 10g, Zi Su Zi 10g, Kuan Dong Hua 20g, Zhi Mu 20g, Sang Bai Pi 20g, Ban Xia 10g, Shi Gao 20g, Ma Huang 6g	150 mL, twice per day (300 mL/dose)	Quality control standards for drugs in compliance with the Chinese Pharmacopoeia	Yes
Hua WS 2017	MXSG	Phlegm heat accumulating in the lung pattern	Ma Huang 5g, Ku Xing Ren 10g, Shi Gao 20g, Huang Qin 12g, She Gan 12g, Jie Geng 12g, Zi Wan 12g, Kuan Dong Hua 12g, Hai Fu Shi 30g, Ting Li Zi 30g	0.5 dose, twice per day	Quality control standards for drugs in compliance with the Chinese Pharmacopoeia	Yes
Han WL 2025	DC	Phlegm heat accumulating in the lung pattern	Bai Guo 9g, Ma Huang 9g, Ban Xia 9g, Kuan Dong Hua 9g, Sang Bai Pi 9g, Zi Su Zi 6g, Huang Qin 6g, Ku Xing Ren 4.5g, Gan Cao 3g	200 mL, twice per day (400 mL/dose)	Quality control standards for drugs in compliance with the Chinese Pharmacopoeia	Yes
Lu GL 2025	XQL	Phlegm dampness accumulating in the lung pattern	Ma Huang 15g, Bai Shao 15g, Gui Zhi 15g, Ban Xia 15g, Gan Jiang 10g, Gan Cao 10g, Wu Wei Zi 10g, Xi Xin 3g	150 mL, twice per day (300 mL/dose)	Quality control standards for drugs in compliance with the Chinese Pharmacopoeia	Yes
Le X 2025	SZJQ	NR	Zi Su Zi 15g, Qian Hu 12g, Ban Xia 12g, Dang Gui 10g, Hou Po 10g, Sheng Jiang 6g, Rou Gui 6g, Gan Cao 6g	100 mL, three times per day (300 mL/dose)	Quality control standards for drugs in compliance with the Chinese Pharmacopoeia	Yes
Qiu ZF 2024	XQL	Exterior cold with interior fluid retention pattern	Ma Huang 9g, Bai Shao 9g, Gui Zhi 9g, Wu Wei Zi 9g, Ban Xia 9g, Xi Xin 3g, Gan Jiang 6g, Gan Cao 6g	150 mL, twice per day (300 mL/dose)	Quality control standards for drugs in compliance with the Chinese Pharmacopoeia	Yes

NR: not reported.

[a]The dosage provided here is 1 dose (Chinese Name is used for brevity).

Note: Some herbs such as *Ephedra sinica* can be toxic, and some have been classified as endangered by the IUCN (e.g. *Ginkgo biloba*). The applications of herbs are advised to comply with relevant regulations.

Appendix Table A.17: Decoction protocols for herbal formulas in the included studies

Study ID	Formula name	Decoction detail
Wang HX 2023	XQL	Decoct twice.
Ma JQ 2022	XQL	Decoct twice: first for 1 hour, second for 40 minutes; combine the liquids.
Yan HF 2020	DC	Standard decoction method.
Xu XP 2022	MXSG	Decoction of gypsum first.
Mei Y 2022	WJ	The hospital uniformly decocts the herbs.
Jiang LX 2021	SZJQ	Add 800 mL water, soak for 50 minutes, boil over high heat, then simmer for 25 minutes; decoct twice and combine the liquids.
Pan XD 2018	XQL	Decoct with 300 mL water.
Wang XD 2017	MXSG	The hospital uniformly decocts the herbs.
Zhang Y 2023	ECSZYQ	The hospital uniformly decocts the herbs.
Hao SR 2018	WJ	The hospital uniformly decocts the herbs.
Zhao DL 2020	QQHT	The hospital uniformly decocts the herbs.
Yu XD 2022	ECSZYQ	Add 800 mL water, soak for over 3 hours; first decoction: boil over high heat, then simmer for 30 minutes to yield 200 mL; second decoction: add 500 mL hot water, boil over high heat, then simmer for 20 minutes to yield 200 mL; combine the liquids.
Liu GJ 2024	SBP	Decoct twice and combine the liquids.
Liu YC 2022	SBP	Decoct with 500 mL water to yield 300 mL liquid.
Zheng X 2018	SZJQ	The hospital uniformly decocts the herbs.
Yuan C 2021	SZJQ	The hospital uniformly decocts the herbs.
Chen DM 2020	SZJQ	Decoct with 500 mL water to yield 300 mL liquid.
Yang BJ 2018	SZJQ	Add 400 mL water, soak for 30 minutes, decoct for 30 minutes to yield 200 mL; add 200 mL water, decoct for 20 minutes to yield another 200 mL; combine the liquids.
Yuan LD 2017	WJ	The hospital uniformly decocts the herbs.
Liu SN 2022	WJ	Decoct twice to yield 300 mL liquid.
Zhang HL 2023	XQL	Decoct with 400 mL water to yield 200 mL liquid.
Wu YW 2015	XQL	Decoct ephedra first, skim foam, then decoct with other herbs to yield 200 mL liquid.
Chen R 2021	XQL	The hospital uniformly decocts the herbs using the standard method to yield 300 mL liquid.
Zhang Y 2020	XQL	Soak in cold water for 30 minutes, then decoct twice.
Zhang MC 2022	XQL	The hospital uniformly decocts the herbs using the standard method to yield 300 mL liquid.
Yu L 2015	XQL	The hospital uniformly decocts the herbs.
Xu XM 2016	XQL	Decoct with 900 mL water to yield 500 mL liquid.
Gu YY 2019	XBCQ	Add rhubarb later.
Zhuang X 2020	SBP	The hospital uniformly decocts the herbs.
Xiao LS 2021	YBBX	Add 400 mL water, decoct ephedra first, skim foam, then add other herbs and decoct to 100 mL liquid.
Guo WX 2007	XBCQ	Decoction of gypsum first, rhubarb added later.
Wu CH 2015	WJ	The hospital uniformly decocts the herbs, using 500 mL water to yield 150 mL liquid.
Hua WS 2017	MXSG	Wrap lepidium seed in cloth for decoction.
Lu GL 2025	XQL	Decoct with 600 mL water to yield 300 mL liquid.

Appendix Table A.18: Pairwise random-effects meta-analyses for all outcomes

Intervention	FEV_1 (L)				FEV_1%pred (%)				FEV_1/FVC (%)			
	No. of RCTs	Patients	MD (95% CrI)	I^2(%)	No. of RCTs	Patients	MD (95% CrI)	I^2(%)	No. of RCTs	Patients	MD (95% CrI)	I^2(%)
DC + CT vs CT	6	512	0.37 (0.27, 0.48)	74	2	212	7.52 (1.99, 13.05)	77.5	4	388	5.85 (2.64, 9.07)	91.6
ECSZYQ + CT vs CT	8	978	0.39 (0.28, 0.49)	65.1	7	654	8.04 (5.01, 11.07)	89	13	1248	9.11 (6.24, 11.98)	95.9
MXSG + CT vs CT	12	1007	0.47 (0.31, 0.63)	95.6	10	1210	6.53 (4.55, 8.51)	91.5	19	1996	5.40 (4.66, 6.14)	62.9
QQHT + CT vs CT	5	596	0.47 (0.37, 0.56)	0	4	738	8.77 (6.68, 10.86)	57	5	696	7.95 (6.84, 9.05)	0
SBP + CT vs CT	9	880	0.54 (0.29, 0.79)	95.7	3	325	3.46 (2.20, 4.73)	15.6	8	800	10.33 (6.63, 14.04)	95.5
SZJQ + CT vs CT	17	1502	0.31 (0.23, 0.39)	88	10	770	4.05 (3.38, 4.71)	23.9	18	1630	6.93 (5.16, 8.70)	90.2
WJ + CT vs CT	9	905	0.56 (0.15, 0.96)	98.8	5	496	6.52 (2.47, 10.57)	92.4	6	665	10.40 (4.81, 16.00)	93.5
XBCQ + CT vs CT	1	120	0.25 (0.19, 0.31)	N/A	3	246	4.54 (2.54, 6.53)	0	4	384	5.52 (4.36, 6.68)	29.3
XQL + CT vs CT	20	1957	0.39 (0.29, 0.49)	90.5	11	1061	5.13 (4.05, 6.22)	71.7	22	2101	6.25 (5.42, 7.08)	95.4
YBBX + CT vs CT	2	200	0.25 (-0.04, 0.53)	88.4	2	160	6.79 (-1.66, 15.23)	93.9	2	190	4.58 (2.52, 6.63)	46.1

Intervention	PaO_2 (mmHg)				$PaCO_2$ (mmHg)				Effective rate (%)			
	No. of RCTs	Patients	MD (95% CrI)	I^2(%)	No. of RCTs	Patients	MD (95% CrI)	I^2(%)	No. of RCTs	Patients	OR (95% CrI)	I^2(%)
DC + CT vs CT	2	188	7.70 (-0.49, 15.90)	92.6	2	188	-6.23 (-12.96, -0.49)	95.9	7	644	4.91 (2.97, 8.12)	0
ECSZYQ + CT vs CT	2	232	5.31 (1.92, 8.70)	71.2	2	232	-5.88 (-8.69, -3.07)	55.7	14	1332	4.58 (3.10, 6.78)	0
MXSG + CT vs CT	3	279	12.55 (10.73, 14.38)	12.6	3	279	-10.24(-12.35,-8.13)	66.2	20	2061	3.53 (2.55, 4.90)	0
QQHT + CT vs CT	4	410	9.94 (3.44, 16.45)	91.7	4	410	-5.17 (-7.42, -2.92)	68	8	1006	6.48 (4.01, 10.48)	0
SBP + CT vs CT	3	240	6.37 (-0.08, 12.83)	93.9	3	240	-5.89 (-11.88, 0.10)	96	8	860	4.09 (2.63, 6.35)	0
SZJQ + CT vs CT	12	1086	8.22 (5.48, 10.96)	91.7	12	1086	-7.06 (-9.08, -5.05)	91	20	1764	3.91 (2.86, 5.36)	0
WJ + CT vs CT	1	116	2.97 (1.70, 4.24)	N/A	1	116	-3.95 (-5.47, -2.43)	N/A	9	909	5.10 (3.17, 8.19)	0
XBCQ + CT vs CT	-	-	-	-	-	-	-	-	5	454	3.95 (2.12, 7.32)	0
XQL + CT vs CT	9	950	10.70 (5.63, 15.76)	95.7	9	950	-9.10 (-11.94, -6.26)	89.9	23	2144	5.01 (3.77, 6.66)	0
YBBX + CT vs CT	2	170	10.60 (6.66, 14.54)	47.4	2	170	-6.57 (-19.27, 6.14)	96.2	2	210	6.50 (2.10, 20.12)	0

MD: mean difference, CrI: credible interval, OR: odds ratio.

Appendix Table A.19: Summary of adverse events

Study ID	Adverse events	
	I	C
Feng J 2023	Fatigue in 1 case	Diarrhea in 2 cases, transient liver impairment in 1 case, fatigue in 5 cases
Xing HZ 2020	Nausea in 2 cases, headache in 1 case	Nausea in 1 case, drowsiness in 1 case
Wu JN 2016	Diarrhea in 1 case (resolved after 30 minutes)	Vomiting and diarrhea in 1 case (mild symptoms)
Yan HF 2020	Diarrhea in 3 cases, skin itching in 2 cases, abnormal liver function in 1 case	Diarrhea in 2 cases, nausea and vomiting in 1 case, skin itching in 3 cases, abnormal liver function in 2 cases
Wang W 2021	Nausea in 1 case, gastrointestinal discomfort in 2 cases, insomnia in 2 cases	Insomnia in 1 case, dizziness and headache in 2 cases, gastrointestinal discomfort in 1 case
Mei Y 2022	Dizziness in 1 case, gastrointestinal reaction in 1 case	Dizziness in 1 case, gastrointestinal reaction in 2 cases, fatigue in 1 case
Niu J 2023	Vomiting in 2 cases, diarrhea in 2 cases, rash in 1 case	Vomiting in 1 case, rash in 1 case, diarrhea in 1 case
Bai FR 2024	Diarrhea in 1 case, rash in 1 case, drowsiness in 1 case, dizziness in 1 case	Rash in 1 case, dizziness in 1 case
Sun HJ 2017	None	None
Gong Y 2022	None	None
Gao ZT 2022	Diarrhea in 1 case, abdominal pain in 1 case, dry mouth and nose in 1 case	Rash in 1 case, diarrhea in 2 cases, abdominal pain in 3 cases, dry mouth and nose in 4 cases
Liao Q 2020	None	None
Yan SL 2019	None	None
Hao SR 2018	Nausea and vomiting in 2 cases, ventilator-assisted breathing in 1 case, other adverse events in 1 case	Nausea and vomiting in 2 cases, ventilator-assisted breathing in 2 cases, respiratory failure in 1 case, other adverse events in 2 cases
Ma ZH 2020	None	None
Yao B 2023	None	None
Kou ZJ 2024	Nausea and vomiting in 2 cases, dizziness in 2 cases	Nausea and vomiting in 3 cases, dizziness in 1 case, abdominal distension in 1 case
Wang XH 2023	Nausea in 1 case, dizziness in 2 cases	Nausea in 1 case, dizziness in 1 case
Liu YC 2022	Gastrointestinal discomfort in 1 case, headache in 1 case	Gastrointestinal discomfort in 2 cases, headache in 1 case, drowsiness in 1 case
Liu JJ 2021	Headache in 1 case, gastrointestinal discomfort in 1 case, drowsiness in 1 case	Headache in 2 cases, gastrointestinal discomfort in 2 cases, contact dermatitis in 1 case, drowsiness in 2 cases
Cai B 2018	None	None
Yuan C 2021	None	Transient tachycardia in 1 case
Chen DM 2020	None	None
Wang LY 2022	Nausea in 1 case	Nausea in 1 case, headache in 1 case, drowsiness in 2 cases
Liu JH 2024	None	None
Mao ZX 2019	None	None
Zhang LH 2011	None	Rash in 1 case, nausea and vomiting in 1 case
Zhang LS 2011	Mild abnormal liver function in 3 cases, abnormal renal function in 1 case, gastrointestinal discomfort in 6 cases	Mild abnormal liver function in 2 cases, abnormal renal function in 2 cases, gastrointestinal discomfort in 4 cases

Chen XJ 2019	Nausea and vomiting in 3 cases, dizziness in 1 case	Nausea and vomiting in 2 cases, dizziness in 1 case
Chen R 2021	None	None
Wang HM 2023	None	None
Zhang MC 2022	Abnormal liver function in 2 cases, gastrointestinal reaction in 5 cases, palpitations in 1 case	Abnormal liver function in 3 cases, gastrointestinal reaction in 4 cases, palpitations in 1 case, rash in 1 case
Li JL 2015	None	None
Sun HW 2021	Fever in 1 case, rash in 1 case, nausea and vomiting in 2 cases	Fever in 1 case, dizziness in 1 case, rash in 1 case, nausea and vomiting in 1 case
Zhang WZ 2014	None	None
Wu CH 2015	None	None
Han WL 2025	Nausea and vomiting in 2 cases, rash in 3 cases, tachycardia in 2 cases	Nausea and vomiting in 2 cases, rash in 1 case, tachycardia in 1 case
Le X 2025	Diarrhea in 1 case, dry mouth in 1 case, rash in 1 case, headache in 1 case	Nausea and vomiting in 1 case, diarrhea in 1 case, dry mouth in 2 cases, rash in 1 case, headache in 1 case
Qiu ZF 2024	None	None

I/C: intervention/control groups

Appendix Table A.20–A.21: Subgroup analysis

Appendix Table A.20: Summary of subgroups with significant between-group effect size difference

Comparison	Criteria	Subgroup	No.of RCTs	Outcomes	MD (95% CrI)	*p-value*	Between-group *p-value*
SZJQ + CT vs CT	RoB	All	18	FEV_1/FVC	4.51 (3.30, 5.72)	<0.001	0.027
		Non-high risk	16		7.42 (6.14, 8.71)	<0.001	
		High risk	2		3.36 (-0.01, 6.73)	0.051	
XQL + CT vs CT	Disease duration	All	18	FEV_1/FVC	6.16 (5.27, 7.05)	<0.001	0.01
		≤ 7	9		4.56 (3.76, 5.35)	<0.001	
		> 7	9		7.41 (5.38, 9.45)	<0.001	
MXSG + CT vs CT	Disease duration	All	11	FEV_1/FVC	4.92 (3.90, 5.94)	<0.001	<0.001
		≤ 7	4		5.95 (5.56, 6.34)	<0.001	
		> 7	7		3.84 (3.13, 4.56)	<0.001	
SZJQ + CT vs CT	Mean of sample size	all	18	FEV_1/FVC	6.93 (5.16, 8.70)	<0.001	<0.001
		≤ 100	15		7.56 (6.35, 8.78)	<0.001	
		> 100	3		2.94 (0.87, 5.01)	0.005	
SZJQ + CT vs CT	Mean of sample size	All	17	FEV_1	0.31 (0.23, 0.39)	<0.001	<0.001
		≤ 100	15		0.35 (0.25, 0.44)	<0.001	
		> 100	2		0.10 (0.06, 0.15)	<0.001	
XQL + CT vs CT	Processing	All	11	FEV_1 %pred	5.14 (4.05, 6.22)	<0.001	0.029
		Standard	3		6.69 (5.41, 7.98)	<0.001	
		Non-standard	8		4.56 (3.15, 5.97)	<0.001	

CT: conventional therapy, MD: mean difference, CrI: credible interval

Appendix Table A.21: Summary of subgroups where stratification reduced overall heterogeneity

Comparison	Criteria	Subgroup	No. of RCTs	Outcomes	MD (95% CrI)	*p-value*	Heterogeneity	
							I^2	*p-value*
MXSG + CT vs CT	Disease duration	All	11	FEV_1/FVC	4.92 (3.90, 5.94)	<0.001	65.90%	0.001
		≤ 7	4		5.95 (5.56, 6.34)	<0.001	0.00%	0.530
		> 7	7		3.84 (3.13, 4.56)	<0.001	0.00%	0.965

CT: conventional therapy, MD: mean difference, CrI: credible interval

Appendix Table A.22: Evidence strengths of pairwise meta-analyses with the GRADE approach

Comparisons	Outcomes	Preliminary	RoB	Inconsistency	Indirectness	Imprecision	Publication Bias	Strength
DC + CT vs CT	FEV_1	High	↓	↓			↓	Very low
ECSZYQ + CT vs CT	FEV_1	High	↓	↓			↓	Very low
MXSG + CT vs CT	FEV_1	High	↓	↓↓			↓	Very low
QQHT + CT vs CT	FEV_1	High	↓				↓	Low
SBP + CT vs CT	FEV_1	High	↓	↓↓			↓	Very low
SZJQ + CT vs CT	FEV_1	High	↓	↓↓			↓	Very low
WJ + CT vs CT	FEV_1	High	↓	↓↓			↓	Very low
XQL + CT vs CT	FEV_1	High	↓	↓↓			↓	Very low
YBBX + CT vs CT	FEV_1	High	↓↓	↓↓		↓	↓	Very low
DC + CT vs CT	FEV_1%pred	High	↓↓	↓↓		↓	↓	Very low
ECSZYQ + CT vs CT	FEV_1%pred	High	↓	↓↓			↓	Very low
MXSG + CT vs CT	FEV_1%pred	High	↓	↓↓			↓	Very low
QQHT + CT vs CT	FEV_1%pred	High	↓	↓			↓	Very low
SBP + CT vs CT	FEV_1%pred	High	↓			↓	↓	Very low
SZJQ + CT vs CT	FEV_1%pred	High	↓				↓	Low
WJ + CT vs CT	FEV_1%pred	High	↓	↓↓			↓	Very low
XBCQ + CT vs CT	FEV_1%pred	High	↓			↓	↓	Very low
XQL + CT vs CT	FEV_1%pred	High	↓	↓			↓	Very low
YBBX + CT vs CT	FEV_1%pred	High	↓	↓↓		↓	↓	Very low
DC + CT vs CT	FEV_1/FVC	High	↓	↓↓		↓	↓	Very low
ECSZYQ + CT vs CT	FEV_1/FVC	High	↓	↓↓			↓	Very low
MXSG + CT vs CT	FEV_1/FVC	High	↓	↓			↓	Very low
QQHT + CT vs CT	FEV_1/FVC	High	↓				↓	Low
SBP + CT vs CT	FEV_1/FVC	High	↓	↓↓			↓	Very low
SZJQ + CT vs CT	FEV_1/FVC	High	↓	↓↓			↓	Very low
WJ + CT vs CT	FEV_1/FVC	High	↓	↓↓			↓	Very low

XBCQ + CT vs CT	FEV_1/FVC	High	↓			↓	↓	Very low
XQL + CT vs CT	FEV_1/FVC	High	↓	↓↓			↓	Very low
YBBX + CT vs CT	FEV_1/FVC	High	↓			↓	↓	Very low
DC + CT vs CT	PaO_2	High	↓	↓↓		↓		Very low
ECSZYQ + CT vs CT	PaO_2	High	↓	↓		↓		Very low
MXSG + CT vs CT	PaO_2	High	↓			↓		Low
QQHT + CT vs CT	PaO_2	High	↓	↓↓				Very low
SBP + CT vs CT	PaO_2	High	↓	↓↓		↓		Very low
SZJQ + CT vs CT	PaO_2	High	↓	↓↓				Very low
XQL + CT vs CT	PaO_2	High	↓	↓↓				Very low
YBBX + CT vs CT	PaO_2	High	↓↓	↓↓		↓		Very low
DC + CT vs CT	$PaCO_2$	High	↓	↓↓		↓	↓	Very low
ECSZYQ + CT vs CT	$PaCO_2$	High	↓	↓		↓	↓	Very low
MXSG + CT vs CT	$PaCO_2$	High	↓	↓		↓	↓	Very low
QQHT + CT vs CT	$PaCO_2$	High	↓	↓			↓	Very low
SBP + CT vs CT	$PaCO_2$	High	↓	↓↓		↓	↓	Very low
SZJQ + CT vs CT	$PaCO_2$	High	↓	↓↓			↓	Very low
XQL + CT vs CT	$PaCO_2$	High	↓	↓↓			↓	Very low
YBBX + CT vs CT	$PaCO_2$	High	↓↓	↓↓		↓	↓	Very low
DC + CT vs CT	ER	High	↓				↓	Low
ECSZYQ + CT vs CT	ER	High	↓				↓	Low
MXSG + CT vs CT	ER	High	↓				↓	Low
QQHT + CT vs CT	ER	High	↓				↓	Low
SBP + CT vs CT	ER	High	↓				↓	Low
SZJQ + CT vs CT	ER	High	↓				↓	Low
WJ + CT vs CT	ER	High	↓				↓	Low
XBCQ + CT vs CT	ER	High	↓				↓	Low

XQL + CT vs CT	ER	High	↓				↓	Low
YBBX + CT vs CT	ER	High	↓			↓	↓	Very low

CT: conventional therapy, ER: effective rate.

Appendix Table A.23–A.28: Sensitivity analysis by excluding all high-risk RCTs

Appendix Table A.23: FEV_1

60.80%											
DC + CT	50.30%										
0.05 (-0.33, 0.43)	ECSZYQ + CT	64.90%									
-0.02 (-0.35, 0.33)	-0.07 (-0.38, 0.24)	MXGS + CT	53.90%								
0.04 (-0.40, 0.46)	-0.01 (-0.42, 0.39)	0.05 (-0.32, 0.44)	QQHT + CT	3.30%							
**0.48 (0.19, 0.76)**	**0.42 (0.18, 0.67)**	**0.49 (0.30, 0.68)**	**0.44 (0.11, 0.77)**	CT	73.40%						
-0.06 (-0.42, 0.28)	-0.12 (-0.43, 0.20)	-0.05 (-0.33, 0.23)	-0.10 (-0.49, 0.29)	**-0.54 (-0.75, -0.33)**	SBP + CT	32.00%					
0.14 (-0.19, 0.47)	0.09 (-0.21, 0.38)	0.15 (-0.09, 0.40)	0.10 (-0.26, 0.46)	**-0.34 (-0.50, -0.18)**	0.20 (-0.06, 0.47)	SZJQ + CT	83.70%				
-0.14 (-0.52, 0.25)	-0.19 (-0.54, 0.17)	-0.12 (-0.44, 0.20)	-0.18 (-0.59, 0.24)	**-0.62 (-0.87, -0.36)**	-0.08 (-0.41, 0.25)	-0.28 (-0.58, 0.02)	WJ + CT	31.20%			
0.23 (-0.43, 0.92)	0.19 (-0.47, 0.83)	0.25 (-0.38, 0.89)	0.20 (-0.49, 0.90)	-0.24 (-0.85, 0.37)	0.30 (-0.33, 0.95)	0.09 (-0.53, 0.73)	0.37 (-0.28, 1.03)	XBCQ + CT	45.90%		
0.07 (-0.26, 0.38)	0.02 (-0.27, 0.30)	0.08 (-0.15, 0.31)	0.03 (-0.33, 0.39)	**-0.41 (-0.55, -0.27)**	0.13 (-0.13, 0.39)	-0.07 (-0.28, 0.14)	0.21 (-0.09, 0.50)	-0.17 (-0.80, 0.45)	XQL + CT	48.90%	
0.08 (-0.61, 0.77)	0.03 (-0.65, 0.71)	0.10 (-0.57, 0.75)	0.04 (-0.65, 0.74)	-0.40 (-1.02, 0.22)	0.14 (-0.51, 0.80)	-0.06 (-0.70, 0.58)	0.22 (-0.44, 0.89)	-0.15 (-1.03, 0.72)	0.02 (-0.63, 0.65)	YBBX + CT	

SUCRA values

Treatments

Appendix Table A.24: FEV_1%pred

43.40%										
DC + CT	80.90%									
-3.19 (-10.66, 4.27)	ECSZYQ + CT	63.40%								
-1.54 (-9.07, 5.50)	1.55 (-2.35, 5.41)	MXGS + CT	85.50%							
-3.70 (-11.48, 3.97)	-0.57 (-5.19, 4.19)	-2.13 (-6.40, 2.48)	QQHT + CT	1.70%						
5.04 (-1.85, 11.76)	**8.15 (5.14, 11.21)**	**6.62 (4.27, 9.17)**	**8.73 (5.04, 12.32)**	CT	28.00%					
1.43 (-6.52, 9.27)	4.56 (-0.52, 9.63)	3.04 (-1.51, 7.82)	5.14 (-0.26, 10.45)	-3.58 (-7.53, 0.34)	SBP + CT	34.80%				
0.55 (-6.77, 7.64)	3.65 (-0.19, 7.40)	2.13 (-1.15, 5.54)	4.22 (-0.11, 8.46)	**-4.52 (-6.86, -2.18)**	-0.93 (-5.56, 3.61)	SZJQ + CT	71.50%			
-2.47 (-10.58, 5.74)	0.66 (-4.44, 6.05)	-0.86 (-5.51, 4.11)	1.25 (-4.33, 6.90)	**-7.48 (-11.67, -3.17)**	-3.90 (-9.53, 1.98)	-2.95 (-7.77, 2.01)	WJ + CT	38.00%		
0.62 (-8.11, 9.26)	3.74 (-2.30, 10.00)	2.19 (-3.62, 8.30)	4.30 (-2.12, 10.83)	-4.44 (-9.80, 1.06)	-0.81 (-7.53, 5.93)	0.08 (-5.64, 6.00)	2.98 (-3.72, 9.85)	XBCQ + CT	43.70%	
-0.13 (-7.42, 6.79)	2.95 (-0.77, 6.77)	1.41 (-1.72, 4.76)	3.52 (-0.70, 7.76)	**-5.22 (-7.32, -3.06)**	-1.62 (-6.05, 2.89)	-0.67 (-3.87, 2.42)	2.29 (-2.64, 6.87)	-0.77 (-6.63, 4.91)	XQL + CT	59.70%
-1.54 (-10.22, 6.87)	1.64 (-4.40, 7.47)	0.13 (-5.33, 5.62)	2.19 (-4.16, 8.29)	**-6.53 (-11.60, -1.56)**	-2.97 (-9.49, 3.48)	-2.04 (-7.51, 3.44)	0.94 (-5.70, 7.37)	-2.13 (-9.56, 5.03)	-1.32 (-6.79, 4.08)	YBBX + CT

SUCRA values

Treatments

Appendix Table A.25: FEV_1/FVC

25.80%											
DC + CT	79.00%										
**-4.80 (-9.03, -0.42)**	ECSZYQ + CT	36.20%									
-1.30 (-5.38, 2.94)	**3.52 (0.77, 6.22)**	MXGS + CT	61.70%								
-3.14 (-8.14, 1.99)	1.62 (-2.46, 5.74)	-1.89 (-5.70, 1.98)	QQHT + CT	0.50%							
**4.60 (0.99, 8.45)**	**9.38 (7.27, 11.52)**	**5.87 (4.13, 7.64)**	**7.76 (4.39, 11.13)**	CT	87.70%						
**-5.83 (-10.44, -1.12)**	-1.05 (-4.43, 2.41)	**-4.57 (-7.69, -1.26)**	-2.70 (-7.04, 1.73)	**-10.45 (-13.04, -7.79)**	SBP + CT	59.30%					
-2.84 (-6.91, 1.37)	1.92 (-0.86, 4.75)	-1.58 (-4.05, 0.87)	0.30 (-3.64, 4.13)	**-7.44 (-9.24, -5.68)**	3.01 (-0.24, 6.21)	SZJQ + CT	95.80%				
**-7.52 (-12.77, -2.05)**	-2.72 (-7.02, 1.75)	**-6.24 (-10.43, -1.92)**	-4.37 (-9.40, 0.89)	**-12.10 (-15.85, -8.15)**	-1.67 (-6.30, 3.00)	**-4.65 (-8.83, -0.30)**	WJ + CT	30.00%			
-0.54 (-5.48, 4.54)	**4.25 (0.27, 8.33)**	0.73 (-3.08, 4.59)	2.58 (-2.32, 7.39)	**-5.12 (-8.53, -1.70)**	**5.31 (0.99, 9.66)**	2.30 (-1.57, 6.22)	**6.95 (1.68, 12.04)**	XBCQ + CT	48.80%		
-2.14 (-6.17, 1.92)	**2.61 (0.04, 5.18)**	-0.90 (-3.18, 1.41)	1.01 (-2.84, 4.70)	**-6.77 (-8.33, -5.25)**	**3.68 (0.56, 6.71)**	0.68 (-1.63, 3.01)	**5.37 (1.11, 9.38)**	-1.62 (-5.44, 2.09)	XQL + CT	24.30%	
0.40 (-5.54, 6.60)	**5.22 (0.01, 10.39)**	1.67 (-3.23, 6.72)	3.55 (-2.24, 9.45)	-4.14 (-8.84, 0.56)	**6.27 (0.80, 11.61)**	3.30 (-1.73, 8.27)	**7.91 (1.76, 13.97)**	0.96 (-5.02, 6.85)	2.58 (-2.40, 7.54)	YBBX + CT

SUCRA values

Treatments

Appendix Table A.26: PaO_2

48.13%									
DC + CT	30.75%								
2.51 (-7.41, 12.47)	ECSZYQ + CT	84.38%							
-4.67 (-13.34, 4.15)	-7.20 (-16.20, 1.75)	MXGS + CT	52.63%						
-0.50 (-9.47, 8.55)	-3.00 (-12.02, 6.15)	4.24 (-3.82, 12.19)	QQHT + CT	1.13%					
**7.85 (0.99, 14.81)**	5.27 (-1.47, 12.28)	**12.54 (6.97, 18.19)**	**8.34 (2.47, 14.10)**	CT	37.38%				
1.39 (-7.50, 10.13)	-1.15 (-10.07, 8.03)	6.07 (-1.87, 14.03)	1.90 (-6.18, 9.90)	**-6.44 (-12.01, -0.81)**	SBP + CT	42.63%			
0.37 (-7.27, 7.82)	-2.16 (-9.58, 5.43)	5.09 (-1.39, 11.46)	0.91 (-5.82, 7.35)	**-7.46 (-10.61, -4.39)**	-0.98 (-7.51, 5.42)	SZJQ + CT	73.38%		
-2.77 (-10.56, 4.88)	-5.29 (-12.81, 2.37)	1.92 (-4.53, 8.47)	-2.30 (-8.83, 4.38)	**-10.62 (-13.96, -7.34)**	-4.16 (-10.69, 2.26)	-3.17 (-7.81, 1.45)	XQL + CT	78.63%	
-4.63 (-16.92, 7.59)	-7.20 (-19.32, 4.71)	0.03 (-11.53, 11.54)	-4.21 (-15.72, 7.39)	**-12.54 (-22.55, -2.39)**	-6.12 (-17.54, 5.46)	-5.13 (-15.52, 5.64)	-1.91 (-12.49, 8.56)	YBBX + CT	

SUCRA values

Treatments

Appendix Table A.27: $PaCO_2$

45.13%								
DC + CT	39.50%							
-0.68 (-9.00, 7.73)	ECSZYQ + CT	78.75%						
3.78 (-3.67, 11.21)	4.42 (-3.26, 12.12)	MXGS + CT	31.50%					
-1.51 (-9.05, 6.26)	-0.84 (-8.36, 7.04)	-5.23 (-12.06, 1.75)	QQHT + CT	1.13%				
**-6.28 (-12.08, -0.33)**	-5.62 (-11.58, 0.52)	**-10.05 (-14.75, -5.20)**	-4.80 (-9.72, 0.13)	CT	39.88%			
-0.48 (-7.93, 7.05)	0.17 (-7.47, 8.03)	-4.30 (-11.00, 2.56)	1.02 (-5.87, 7.91)	**5.77 (0.98, 10.59)**	SBP + CT	52.13%		
0.88 (-5.60, 7.39)	1.49 (-4.96, 8.05)	-2.91 (-8.47, 2.52)	2.31 (-3.27, 7.91)	**7.10 (4.42, 9.75)**	1.32 (-4.22, 6.83)	SZJQ + CT	73.75%	
2.78 (-3.65, 9.48)	3.44 (-3.04, 10.28)	-0.98 (-6.45, 4.74)	4.28 (-1.46, 10.03)	**9.10 (6.25, 11.92)**	3.32 (-2.38, 8.97)	1.99 (-1.92, 5.97)	XQL + CT	89.13%
6.96 (-3.73, 17.98)	7.59 (-3.09, 18.44)	3.09 (-7.06, 13.49)	8.33 (-1.92, 18.86)	**13.19 (3.97, 22.41)**	7.37 (-3.03, 17.63)	6.10 (-3.56, 15.61)	4.10 (-5.59, 13.68)	YBBX + CT

SUCRA values

Treatments

Appendix Table A.28: Effective rate

69.90%										
DC + CT	44.70%									
1.33 (0.62, 2.80)	ECSZYQ + CT	29.50%								
1.54 (0.77, 3.23)	1.18 (0.67, 2.00)	MXGS + CT	90.60%							
0.70 (0.32, 1.67)	0.54 (0.27, 1.02)	**0.46 (0.23, 0.88)**	QQHT + CT	0.00%						
**5.85 (3.28, 11.68)**	**4.45 (2.95, 6.83)**	**3.86 (2.75, 5.65)**	**8.48 (5.11, 14.34)**	CT	37.50%					
1.42 (0.69, 3.25)	1.08 (0.60, 1.97)	0.93 (0.53, 1.74)	**2.03 (1.04, 4.31)**	**0.24 (0.16, 0.38)**	SBP + CT	43.20%				
1.33 (0.69, 2.91)	1.00 (0.59, 1.78)	0.87 (0.55, 1.43)	**1.91 (1.03, 3.61)**	**0.23 (0.16, 0.31)**	0.95 (0.55, 1.61)	SZJQ + CT	47.80%			
1.29 (0.56, 2.91)	0.98 (0.48, 1.86)	0.83 (0.42, 1.57)	1.81 (0.85, 4.00)	**0.22 (0.12, 0.37)**	0.91 (0.44, 1.79)	0.94 (0.49, 1.80)	WJ + CT	48.00%		
1.26 (0.51, 3.28)	0.98 (0.46, 2.05)	0.85 (0.39, 1.77)	1.85 (0.78, 4.22)	**0.22 (0.11, 0.42)**	0.90 (0.40, 1.97)	0.97 (0.44, 1.93)	0.99 (0.44, 2.42)	XBCQ + CT	63.20%	
1.10 (0.57, 2.39)	0.84 (0.51, 1.39)	0.73 (0.47, 1.17)	1.60 (0.89, 2.95)	**0.19 (0.14, 0.25)**	0.78 (0.46, 1.34)	0.83 (0.54, 1.30)	0.87 (0.49, 1.64)	0.88 (0.42, 1.83)	XQL + CT	77.40%
0.81 (0.16, 2.68)	0.62 (0.13, 1.94)	0.52 (0.12, 1.57)	1.15 (0.21, 3.78)	**0.14 (0.03, 0.38)**	0.57 (0.12, 1.74)	0.61 (0.14, 1.78)	0.63 (0.12, 2.08)	0.62 (0.13, 2.24)	0.71 (0.16, 2.20)	YBBX + CT

SUCRA values

Treatments

Appendix Table A.29–A.31: Sensitivity analysis by protocol-defined CT regimen
Appendix Table A.29: FEV_1

49.67%									
DC + CT	44.56%								
0.03 (-0.60, 0.68)	ECSZYQ + CT	58.00%							
-0.07 (-0.69, 0.52)	-0.11 (-0.60, 0.37)	MXGS + CT	55.11%						
-0.07 (-0.92, 0.81)	-0.10 (-0.90, 0.70)	0.01 (-0.77, 0.79)	QQHT + CT	4.56%					
0.41 (-0.12, 0.92)	0.37 (-0.00, 0.74)	**0.49 (0.17, 0.80)**	0.47 (-0.24, 1.18)	CT	84.56%				
-0.36 (-1.02, 0.30)	-0.39 (-0.95, 0.18)	-0.28 (-0.80, 0.25)	-0.29 (-1.12, 0.54)	**-0.76 (-1.18, -0.34)**	SBP + CT	37.00%			
0.09 (-0.49, 0.65)	0.05 (-0.39, 0.50)	0.17 (-0.24, 0.58)	0.15 (-0.60, 0.90)	**-0.32 (-0.57, -0.07)**	0.45 (-0.04, 0.94)	SZJQ + CT	83.56%		
-0.41 (-1.29, 0.47)	-0.45 (-1.25, 0.36)	-0.33 (-1.12, 0.45)	-0.35 (-1.35, 0.66)	**-0.82 (-1.53, -0.11)**	-0.05 (-0.89, 0.78)	-0.50 (-1.26, 0.25)	WJ + CT	34.00%	
0.15 (-0.71, 1.01)	0.12 (-0.66, 0.90)	0.24 (-0.53, 0.99)	0.22 (-0.75, 1.20)	-0.25 (-0.93, 0.43)	0.51 (-0.28, 1.31)	0.06 (-0.66, 0.80)	0.57 (-0.43, 1.58)	XBCQ + CT	47.67%
0.01 (-0.58, 0.57)	-0.03 (-0.48, 0.40)	0.08 (-0.33, 0.50)	0.07 (-0.68, 0.81)	**-0.40 (-0.66, -0.15)**	0.36 (-0.13, 0.86)	-0.09 (-0.44, 0.27)	0.41 (-0.33, 1.17)	-0.15 (-0.88, 0.58)	XQL + CT

SUCRA values

Treatments

Appendix Table A.30: FEV_1%pred

79.50%						
DC + CT	70.00%					
2.14 (-8.08, 12.23)	ECSZYQ + CT	59.00%				
3.35 (-7.07, 13.10)	1.21 (-5.29, 7.25)	MXGS + CT	76.00%			
1.44 (-9.04, 11.81)	-0.70 (-7.41, 6.05)	-1.85 (-8.12, 4.76)	QQHT + CT	0.67%		
**10.82 (1.34, 19.96)**	**8.65 (3.90, 13.28)**	**7.46 (3.46, 11.84)**	**9.32 (4.40, 14.07)**	CT	25.50%	
6.80 (-3.40, 16.53)	4.63 (-1.31, 10.59)	3.40 (-1.81, 9.09)	5.27 (-0.79, 11.46)	**-4.00 (-7.62, -0.34)**	SZJQ + CT	39.33%
5.31 (-4.80, 15.34)	3.16 (-3.28, 9.44)	1.94 (-3.79, 7.93)	3.80 (-2.59, 10.08)	**-5.51 (-9.55, -1.28)**	-1.50 (-6.99, 3.95)	XQL + CT

SUCRA values

Treatments

Appendix Table A.31: FEV_1/FVC

26.75%								
DC + CT	83.90%							
-6.41 (-14.19, 1.80)	ECSZYQ + CT	39.75%						
-2.20 (-10.16, 6.12)	4.30 (-0.11, 8.59)	MXGS + CT	59.50%					
-4.24 (-13.68, 5.12)	2.16 (-4.42, 8.61)	-2.14 (-8.79, 4.52)	QQHT + CT	3.00%				
3.34 (-3.94, 10.86)	**9.82 (6.87, 12.68)**	**5.54 (2.35, 8.73)**	**7.69 (1.87, 13.58)**	CT	97.63%			
**-11.17 (-19.73, -2.25)**	-4.63 (-10.09, 0.84)	**-8.96 (-14.55, -3.26)**	-6.84 (-14.10, 0.81)	**-14.50 (-18.94, -9.89)**	SBP + CT	44.50%		
-2.59 (-10.41, 5.27)	**3.90 (0.06, 7.75)**	-0.41 (-4.48, 3.77)	1.75 (-4.57, 8.13)	**-5.95 (-8.55, -3.34)**	**8.54 (3.22, 13.71)**	SZJQ + CT	57.60%	
-2.77 (-13.18, 8.24)	3.72 (-4.49, 12.02)	-0.54 (-8.77, 8.02)	1.60 (-8.31, 11.17)	-6.05 (-13.85, 1.71)	8.40 (-0.54, 17.62)	-0.15 (-8.18, 7.96)	XBCQ + CT	49.88%
-3.07 (-10.74, 4.79)	3.38 (-0.38, 7.12)	-0.86 (-4.88, 3.03)	1.21 (-4.97, 7.47)	**-6.42 (-8.76, -4.06)**	**8.08 (2.81, 13.15)**	-0.47 (-3.90, 2.99)	-0.38 (-8.41, 7.62)	XQL + CT

SUCRA values

Treatments

Appendix Table A.32: Sensitivity analysis of FEV_1 by consistent pharmacognostic definition

44.75%							
DC + CT	57.38%						
-0.11 (-0.70, 0.45)	MXGS + CT	67.00%					
-0.21 (-0.79, 0.36)	-0.10 (-0.74, 0.56)	QQHT + CT	15.25%				
0.30 (-0.06, 0.67)	0.41 (-0.03, 0.86)	**0.51 (0.06, 0.98)**	CT	93.75%			
**-0.55 (-1.08, -0.02)**	-0.43 (-1.02, 0.15)	-0.33 (-0.93, 0.23)	**-0.85 (-1.23, -0.49)**	SBP + CT	79.50%		
-0.34 (-0.87, 0.19)	-0.23 (-0.81, 0.38)	-0.13 (-0.73, 0.48)	**-0.64 (-1.03, -0.25)**	0.21 (-0.33, 0.75)	SZJQ + CT	52.13%	
-0.08 (-0.56, 0.42)	0.04 (-0.50, 0.59)	0.14 (-0.43, 0.71)	**-0.38 (-0.69, -0.06)**	0.47 (-0.00, 0.96)	0.27 (-0.23, 0.76)	XQL + CT	40.25%
0.05 (-0.52, 0.61)	0.16 (-0.47, 0.79)	0.26 (-0.36, 0.89)	-0.25 (-0.69, 0.19)	**0.60 (0.03, 1.17)**	0.39 (-0.20, 0.96)	0.13 (-0.42, 0.66)	YBBX + CT

SUCRA values (green)

Treatments (grey)

Appendix Table A.33: Comparison with Previous network meta-analysis

Items	This study	Liu S, et al[1]
PRISMA checklists	PRISMA statements	PRISMA-NMA
Registered protocol	CRD 42024622734	CRD 42016052699
Participant	AECOPD patients	AECOPD patients
Interventions	Conventional therapy with traditional Chinese herbal formulas	Routine pharmacotherapy with traditional Chinese herbal formulas
Dosage forms	Only aqueous decoctions (traditional decoctions, not ready-to-use granules)	Undefined
Outcomes	Primary outcomes: FEV_1, FEV_1%pred, FEV_1/FVC, PaO_2, $PaCO_2$;<br>Secondary outcomes: effective rate, quality of life evaluation, post-treatment recurrence situation, adverse events	Primary outcomes: FEV_1, PaO_2, $PaCO_2$, length of hospital stay;<br>Secondary outcomes: dyspnoea, health-related quality of life; hospital readmission for acute exacerbation, effective rate, adverse events
Databases	CNKI, VIP Database, Wanfang Database, PubMed, CENTRAL, CBM, EMBASE, CINAHL, Web of Science	PubMed, EMBASE, CENTRAL, CINAHL, AMED, CBM, CNKI, VIP Database, Wanfang database
Last search date	August 20, 2024 (search update conducted on November 28, 2025)	December 2016
Computational frameworks	Bayesian methods	Bayesian methods
Data synthesis	Pairwise meta-analysis and network meta-analysis	Pairwise meta-analysis and network meta-analysis
Model selection	Random-effects model	Random-effects model
Publication bias analysis	Egger's test, Funnel plots	Only funnel plots
Additional statistical analysis	Cluster Analysis, Meta-regression, Subgroup Analysis, Sensitivity analysis	Sensitivity analysis
Ranking of treatments	SUCRA	SUCRA
Strength of evidence	The GRADE approaches	No
No. of including ten traditional Chinese herbal formulas	10	6
No. of RCTs	132	55
Sample sizes	13,241	4,560
RCTs quality	Moderate to low	Moderate to low
The most promising regimens	QQHT, XQL	QQHT, MXSG
Quality of evidence	Low to very low	No
Results on FEV_1		
No. of available RCTs	89	18
First ranking	WJ	SBP
No. of first ranking's RCTs	9	2
Results on PaO_2		
No. of available RCTs	38	20
First ranking	MXSG	QQHT
No. of first ranking's RCTs	3	3
Results on $PaCO_2$		
No. of available RCTs	38	20
First ranking	MXSG	MXSG
No. of first ranking's RCTs	3	2
Results on effective rate		
No. of available RCTs	116	49
First ranking	QQHT	QQHT
No. of first ranking's RCTs	8	7

GRADE: Grading of Recommendations and Assessment, Development, and Evaluation. PRISMA: Preferred Reporting Items for Systematic Review and Meta-analysis. SUCRA: Surface Under the Cumulative Ranking Curve. AMED: Allied and Complementary Medicine Database. CENTRAL: Cochrane Central Register of Controlled Trials. CBM: Chinese Biomedical Database. CINAHL: Cumulative Index to Nursing and Allied Health Literature. CNKI: China National Knowledge Infrastructure. VIP Database: Chongqing VIP Information Chinese Science and Technology Journal Databse. Wanfang Database: Wanfang Data Knowledge Service Platform.

References

1. Liu, S., Chen, J., Zuo, J., Lai, J., Wu, L., and Guo, X. (2019). Comparative effectiveness of six Chinese herb formulas for acute exacerbation of chronic obstructive pulmonary disease: a systematic review and network meta-analysis. BMC Complement Altern Med 19(1), 226. doi: 10.1186/s12906-019

Appendix Table A.34–A.35: Raw data of outcome measures after treatment

Appendix Table A.34. FEV_1, FEV_1%pred, and FEV_1/FVC data

Intervention Group						Control Group					
FEV_1 (L)		FEV_1%pred (%)		FEV_1/FVC (%)		FEV_1 (L)		FEV_1%pred (%)		FEV_1/FVC (%)	
Mean	SD	Mean	SD	Mean	SD	Mean	SD	Mean	SD	Mean	SD
2.91	0.88					2.48	0.74				
2.76	0.88			72.52	1.85	2.02	0.61			65.36	1.41
2.39	0.28					1.81	0.59				
		69.61	11.74					58.87	11.81		
0.9	0.3			80	30	0.7	0.2			60	20
2.01	0.31			69.36	7.29	1.71	0.27			61.3	6.8
		70.51	6.58	51.85	6.12			64.63	5.96	44.43	6.31
1.73	0.88					1.31	0.59				
		46.09	6.86	62.3	4.48			44.01	6.12	59.98	5.62
		63.12	9.63	52.14	5.01			50.1	2.71	40.04	3.12
2.02	0.42			67.43	7.46	1.79	0.37			63.51	7.11
		63.12	9.68	52.14	5.02			50.09	2.74	40.04	3.15
2.52	0.48					1.93	0.54				
		69.37	4.81	67.39	5.61			62.38	5.69	62.34	4.12
3.41	0.46			87.32	9.72	3.01	0.56			80.32	8.89
2.89	0.36			71.3	11.26	1.37	0.21			52.4	7.34
		65.04	7.57	63.62	4.84			58.72	7.36	58.13	5.09
		70.62	6.71	51.92	6.33			65.26	5.18	43.29	6.18
3.58	1.68	49.39	5.01	78.02	12.45	2.18	1.4	42.36	4.23	62.16	10.12
		49.81	7.6	58.81	7.35			44.76	5.46	55.79	6.43
2.5	0.25			69.31	3.34	2.12	0.3			65.93	3.5
2.74	0.25			71	16	2.06	0.33			65	12
2.91	1.06			71.18	8.55	2.25	0.81			66.28	8.04
		76.89	7.95	79.47	8.38			69.58	7.03	70.19	7.12
2.77	0.41			72.1	12.32	1.53	0.38			53.8	8.59
2.78	0.21					1.96	0.26				
1.91	0.34	61.91	9.54	54.02	12.72	1.74	0.29	57.16	10.49	48.26	11.38
		57.69	11.43	78.61	12.35			51.63	9.62	72.21	11.36
2.89	0.42	67.16	9.87	68.15	7.26	2.51	0.27	63.42	6.19	65.34	5.21
1.51	0.25			67.31	8.18	1.38	0.24			60.42	9.33
1.85	0.46			74.8	6.4	1.49	0.2			69.45	7.3
2.47	0.57			68	20	1.89	0.55			55	19
2.01	0.16			68.54	7.25	1.76	0.18			62.45	7.31
		63.7	5.5	64.8	6.2			58.3	6.2	60.2	5.8
1.9	0.4					1.5	0.3				
		71.4	4.4	65.2	4.1			68.2	3.8	61.6	3.9
		75.4	5.8	85.7	10.3			62.8	6.9	71.9	15.4
		71.42	4.38	65.19	4.06			68.22	3.83	61.54	3.92
2.65	0.22			71.23	0.94	2.05	0.15			65.33	0.87
2.55	0.59			80.18	10.21	1.97	0.34			72.01	8.59
2.51	0.76			80.41	4.12	1.77	0.53			75.54	5.27
		72.45	4.52	66.58	4.01			69.04	3.49	61.45	3.89
		71.41	4.39	65.18	4.04			68.21	3.81	61.52	3.91
1.88	0.43	88.64	12.48	81.93	11.34	1.53	0.14	69.16	9.88	77.14	10.77
		72.48	4.51	66.69	4.02			65.37	3.48	61.34	3.27

1.14	0.07			57.57	10.06	1.02	0.07			52.41	11.53
1.72	0.37			66.51	8.2	1.44	0.32			59.16	7.28
1.84	0.51			84.12	7.25	1.3	0.48			76.13	6.89
1.37	0.37			78.58	4.67	1.19	0.13			72.42	9.24
3.42	0.73					2.75	0.55				
		60.25	9.68	52.16	6.12			50.12	5.24	40.26	4.58
2.12	0.4			69.4	7.3	1.82	0.38			61.41	6.91
		62.1	2.9	75.1	9.4			59.7	3.2	63.2	9.1
1.87	0.45	58.41	6.04			1.63	0.39	49.12	5.77		
1.93	0.76					1.5	0.57				
		65.14	10.27	60.75	11.37			58.33	9.69	53.25	10.65
		65.09	7.13	58.33	6.41			57.27	3.58	50.38	5.14
		55	19	61.15	18.45			46	18	53.98	17.62
2.02	0.65					1.43	0.57				
1.98	0.42			72.94	7.31	1.58	0.45			64.47	7.07
1.05	0.82	63.4	8.43			0.58	0.28	52.4	7.45		
2.1	0.85			70.95	15.34	1.63	0.74			63.92	14.23
2.13	0.42			94.25	2.36	1.75	0.4			77.09	2.95
4.23	1.39			76.86	11.29	3.57	1.06			62.52	9.63
2.29	0.44			79.51	9.24	1.68	0.47			69.71	10.16
2.08	0.32			70.21	3.06	1.68	0.3			61.32	3.18
2.46	0.47			94	12	1.98	0.43			86	14
1.6	0.83			59.8	8.88	1.23	0.54			54.27	7.89
3.69	1.17			88.37	1.47	2.24	0.81			72.52	1.31
1.85	0.16	43.5	4.35			0.97	0.11	41.23	4.5		
2.34	0.65			78	14	2.1	0.57			60	11
		50.25	4.53					46.26	4.34		
1.51	0.2			67.2	14.41	1.38	0.24			60.35	13.43
		73.11	3.51					68.77	2.89		
1.56	0.42	59.13	7.19	62.12	7.83	1.32	0.38	54.42	9.23	56.27	6.42
1.53	0.17			66.25	6.82	1.44	0.15			62.76	6.32
2.65	0.36	93.51	8.23	88.11	8.28	1.96	0.33	87.56	8.19	80.31	7.37
2.79	0.61			65.24	5.28	2.08	0.66			56.27	4.67
		54.82	2.65					52.1	2.8		
2.79	0.982			59.616	10.112	2.3	0.911			48.563	8.344
1.76	0.31			61.7	6.95	1.55	0.36			55.29	7.16
2.38	0.34			83.35	5.18	2.01	0.21			74.35	4.48
2.4	0.45			83.27	7.06	1.74	0.32			72.65	6.18
2.84	0.24			65.87	5.37	2.46	0.94			58.38	4.26
1.58	0.32			67.54	8.19	1.4	0.29			62.18	7.05
		66.85	2.56	47.3	2.12			62.74	2.62	45.55	1.62
1.64	0.14			55.6	4	1.5	0.14			50.4	4.5
1.56	0.19			68.96	8.36	1.35	0.22			60.01	6.63
		72.22	3.33					69.03	2.91		
1.86	0.53			69.21	7.85	1.57	0.51			57.92	6.22
1.84	0.48			74.81	6.43	1.42	0.33			69.43	7.34
1.86	0.47	58.39	6.06			1.62	0.45	49.01	5.81		
1.41	0.42	69.6	8.85			1.25	0.32	60.34	6.61		
1.89	0.57			82.75	8.13	1.52	0.61			75.94	7.92
1.9	0.35					1.53	0.31				
1.57	0.22					0.94	0.2				

1.28	0.38					1.18	0.28				
1.89	0.72			85.13	8.19	1.59	0.42			75.78	8.16
		69.32	9.42	70.32	6.89			60.98	10.34	63.71	5.82
		84	4	70	3			77	5	66	6
2.21	0.21	68.23	5.87	70.45	7.11	1.97	0.33	62.86	6.74	65.82	8.09
2.31	0.77	65.51	8.11	66.2	7.86	2.41	0.67	63.35	9.32	62.85	8.35
1.48	0.25			48.24	0.29	1.28	0.15			45.51	0.31
1.49	0.34			61.45	11.75	1.25	0.35			52.85	8.81
1.48	0.21			63.32	6.54	1.23	0.23			54.43	5.21
2.32	0.26			69.49	5.47	2.18	0.32			65.52	4.56
		83.15	4.64	71.26	4.08			76.91	6.83	67.15	5.27
1.46	0.21	56.95	0.06	48.62	0.31	1.3	0.14	51.32	0.07	45.21	0.67
1.88	0.62			78.66	8.25	1.53	0.63			73.56	7.94
1.85	0.32			57.26	6.57	1.49	0.22			49.43	6.24
2.57	0.26			73.11	1.15	2.27	0.19			66.82	0.99
				83.56	7.92					78.15	7.41
2.65	1.02			58.63	7.63	1.03	0.34			42.55	5.65
		77.42	4.62	80.1	5.13			77.35	6.19	72.96	4.89
2.61	0.46					1.97	0.41				
		76.8	5.9	73.1	5.8			71.9	5.2	69.3	5.4
				82.71	3.24					76.55	3.52
		60.19	14.05	64.46	10.54			56.56	11.97	60.26	11.94
1.27	0.42			64.72	10.12	0.98	0.46			54.16	9.51
1.43	0.23					1.32	0.25				
2.2	0.4			66.2	2.3	1.8	0.5			61	2.2
		58.72	6.78	60.13	6.38			56.14	1.46	57.32	7.82
		70.87	8.93					67.16	12.43		
		73.26	8.04	63.35	6.29			69.98	7.35	58.84	6.23
		58.93	2.13					53.56	4.35		
1.72	0.22	69.16	16.21	77.15	20.14	1.73	0.34	68.39	17.52	76.22	19.28
		60.3	8.7					49.1	8.1		
		63.77	5.59	64.8	6.52			58.39	6.26	60.29	5.18
1.65	0.08					1.34	0.06				
		55.42	5.31					51.26	5.14		
2.11	0.22	86.35	8.15			1.74	0.25	80.86	7.96		
3.01	0.59					2.37	0.42				

SD: Standard deviation.

Appendix Table A.35. PaO_2, $PaCO_2$, and effective rate data

Intervention Group						Control Group					
PaO_2 (mmHg)		$PaCO_2$ (mmHg)		Effective rate		PaO_2 (mmHg)		$PaCO_2$ (mmHg)		Effective rate	
Mean	SD	Mean	SD	Sample	Events	Mean	SD	Mean	SD	Sample	Events
				44	41					44	33
83.26	3.74	42.36	4.42	36	33	71.56	4.75	52.01	3.45	36	26
				48	44					48	33
				51	47					51	42
82.17	8.22	43.19	4.32	73	71	75.19	7.52	50.11	5.01	73	60
				56	54					56	45
				42	40					42	33
58.4	6.94	56.45	8.98	46	42	54.88	5.98	60.35	8.21	40	30
				40	39					40	31
				35	34					35	27
				70	61					70	56
				37	35					37	28
				43	40					43	31
				85	82					85	73
79.89	8.58	49.56	4.82	42	40	70.13	9.27	56.98	6.15	42	30
				38	37					38	32
				65	60					64	42
72.11	11.02	56.99	5.28	60	56	68.78	10.96	59.78	5.73	56	35
				45	43					45	34
87.33	7.85	46.78	5.74	49	45	75.08	8.29	55.62	6.88	48	34
86.68	7.6	41.64	3.53	55	54	75.49	7.35	53.46	3.33	55	46
				60	57					60	49
				47	45					47	40
				42	40					42	34
				48	45					47	37
				44	42					44	36
				43	41					43	33
				50	48					50	40
				60	55					60	47
				40	38					39	33
				40	39					40	34
				45	38					41	30
				50	48					50	43
				40	38					40	32
				45	44					45	37
				49	44					49	36
				38	36					38	35
				106	102					106	98
				46	45					46	35
				100	96					100	88
				42	40					40	36
				40	37					40	31

84.12	6.17	47.53	5.48	36	33	69.85	6.41	56.82	6.11	36	26
				39	38					39	34
				75	69					75	62
				43	40					43	27
				40	39					40	34
				60	57					60	43
				40	35					40	22
				51	50					51	43
85.44	10.23	49.57	9.02	47	45	72.36	8.61	54.63	9.27	47	38
				158	153					158	126
74.83	8.51	49.76	9.09	60	56	73.23	8.93	51.43	9.16	60	47
90.26	8.87	44.81	5.74	60	57	76.07	9.23	50.31	6.29	60	49
				45	43					45	36
				104	102					104	66
87.35	12.35	43.25	3.43	38	35	76.21	11.34	50.55	4.1	38	28
				60	56					60	48
				46	44					46	36
				43	40					43	31
				42	40					41	32
				43	40					43	33
77.45	6.13	33.75	7.24			71.07	6.22	46.27	7.27		
				100	92					100	77
82.42	7.72	43.7	4.45	40	38	82.12	8.44	43.26	5.06	40	32
84.12	5.65	38.42	2.85	40	37	72.04	4.3	44.35	3.29	40	30
				75	70					75	59
				35	32					35	25
				46	42					46	38
68.38	6.93	40.54	5.46	69	65	64.98	6.64	43.05	4.53	69	56
				40	38					40	31
80.62	5.51	42.59	4.25	47	45	68.3	4.72	48.23	3.94	47	37
64.31	11.25	48.2	11.34	42	40	53.12	10.43	54.35	10.54	42	33
76.441	9.294	45.841	5.223			71.445	7.752	57.673	7.233		
83.16	9.26	45.44	9.7	48	39	76.91	8.15	61.13	11.97	48	28
84.68	6.48	40.24	2.68	40	38	78.34	5.35	47.64	3.478	40	32
86.7	7.16	40.1	2.13	39	37	78.95	6.45	48.19	4.02	39	29
				50	48					50	38
69.05	5.36	40.26	5.19	39	37	63.19	6.08	43.18	6.03	39	31
88.9	7.64	44.02	3.46	60	58	68.6	7.64	55.42	4.76	60	53
83.8	6.6	40.1	5.6	40	35	80	4.5	42.4	4.7	40	33
69.36	4.21	40.14	5.5	44	42	62.28	3.36	47.96	3.35	44	35
				40	35					40	29
				41	37					41	28
				41	39					40	32
				40	38					40	32
75.23	3.51	44.03	4.22			72.26	3.46	47.98	4.15		
78.52	9.38	55.48	8.52	63	59	59.85	8.96	68.59	8.35	63	48
				37	35					37	29
81.5	10.5	53.4	9.7	40	37	68.3	12.2	66.7	10.1	40	22
				48	44					48	35

				38	36					38	30
				47	45					47	35
				50	49					50	36
70.34	5.98	43.24	5.83			71.35	3.13	43.21	5.61		
				40	38					40	30
				35	32					35	26
83.34	7.52	45.42	5.47	45	42	72.22	7.23	53.27	6.19	45	37
				40	37					40	30
				49	46					49	35
				59	56					59	46
80.85	11.57	57.16	7.85			73.43	9.31	69.29	9.84		
69.16	9.54	56.47	6.39	76	71	55.36	8.16	66.15	7.85	76	58
				44	42					43	32
				35	32					35	25
71.46	10.53	55.54	5.64	46	43	56.52	9.31	65.24	6.39	46	33
				40	36					40	32
74.17	8.73	46.93	7.42	58	53	65.14	8.13	57.35	7.64	58	44
				42	40					42	31
				44	42					44	36
				46	44					46	38
82.2	9.38	40.05	4.58			74.33	10.05	40.28	5.33		
				60	59					60	52
				35	31					35	25
				40	38					40	32
76.12	15.49	48.96	11.71	35	33	65.79	16.35	52.15	14.36	35	29
				50	47					50	40
79.6	9.7	46.5	10.3	45	42	67.1	8.8	59.7	11.1	45	32
				40	39					40	32
				40	38					40	32
				40	38					40	32
				36	34					36	27

SD: Standard deviation.

Appendix Table A.36. Program code for network meta-analysis using WinBUGS

model:

```
model {
	for (i in 1:ns) {
		# Likelihood for each arm
		for (k in 1:na[i]) {
			m[i, k] ~ dnorm(theta[i, k], prec[i, k])
			theta[i, k] <- mu[i] + delta[i, k]
			prec[i, k] <- pow(e[i, k], -2)
		}

		# Study-level relative effects
		# The arms are given in the order (arm_1, arm_2, ..., arm_{n_a-1}, arm_{n_a}).
		# The relative effects are parameterized as d[arm_1, arm_k].
		w[i, 1] <- 0
		delta[i, 1] <- 0
		for (k in 2:na[i]) { # parameterize multi-arm trials using a trick to avoid dmnorm
			delta[i, k] ~ dnorm(md[i, k], taud[i, k])
			md[i, k] <- d[t[i, 1], t[i, k]] + sw[i, k]
			taud[i, k] <- tau.d * 2 * (k - 1) / k
			w[i, k] <- delta[i, k] - d[t[i, 1], t[i, k]]
			sw[i, k] <- sum(w[i, 1:k-1]) / (k - 1)
		}
	}

	# Relative effect matrix
	d[1,1] <- 0
	d[1,2] <- -d.CT.DC + d.CT.ECSZYQ
	d[1,3] <- -d.CT.DC + d.CT.MXGS
	d[1,4] <- -d.CT.DC + d.CT.QQHT
	d[1,5] <- -d.CT.DC
	d[1,6] <- -d.CT.DC + d.CT.SBP
	d[1,7] <- -d.CT.DC + d.CT.SZJQ
	d[1,8] <- -d.CT.DC + d.CT.WJ
	d[1,9] <- -d.CT.DC + d.CT.XBCQ
	d[1,10] <- -d.CT.DC + d.CT.XQL
	d[1,11] <- -d.CT.DC + d.CT.YBBX
	d[2,1] <- d.CT.DC + -d.CT.ECSZYQ
	d[2,2] <- 0
	d[2,3] <- -d.CT.ECSZYQ + d.CT.MXGS
	d[2,4] <- -d.CT.ECSZYQ + d.CT.QQHT
	d[2,5] <- -d.CT.ECSZYQ
	d[2,6] <- -d.CT.ECSZYQ + d.CT.SBP
	d[2,7] <- -d.CT.ECSZYQ + d.CT.SZJQ
	d[2,8] <- -d.CT.ECSZYQ + d.CT.WJ
	d[2,9] <- -d.CT.ECSZYQ + d.CT.XBCQ
	d[2,10] <- -d.CT.ECSZYQ + d.CT.XQL
	d[2,11] <- -d.CT.ECSZYQ + d.CT.YBBX
	d[3,1] <- d.CT.DC + -d.CT.MXGS
	d[3,2] <- d.CT.ECSZYQ + -d.CT.MXGS
```

```
d[3,3] <- 0
d[3,4] <- -d.CT.MXGS + d.CT.QQHT
d[3,5] <- -d.CT.MXGS
d[3,6] <- -d.CT.MXGS + d.CT.SBP
d[3,7] <- -d.CT.MXGS + d.CT.SZJQ
d[3,8] <- -d.CT.MXGS + d.CT.WJ
d[3,9] <- -d.CT.MXGS + d.CT.XBCQ
d[3,10] <- -d.CT.MXGS + d.CT.XQL
d[3,11] <- -d.CT.MXGS + d.CT.YBBX
d[4,1] <- d.CT.DC + -d.CT.QQHT
d[4,2] <- d.CT.ECSZYQ + -d.CT.QQHT
d[4,3] <- d.CT.MXGS + -d.CT.QQHT
d[4,4] <- 0
d[4,5] <- -d.CT.QQHT
d[4,6] <- -d.CT.QQHT + d.CT.SBP
d[4,7] <- -d.CT.QQHT + d.CT.SZJQ
d[4,8] <- -d.CT.QQHT + d.CT.WJ
d[4,9] <- -d.CT.QQHT + d.CT.XBCQ
d[4,10] <- -d.CT.QQHT + d.CT.XQL
d[4,11] <- -d.CT.QQHT + d.CT.YBBX
d[5,1] <- d.CT.DC
d[5,2] <- d.CT.ECSZYQ
d[5,3] <- d.CT.MXGS
d[5,4] <- d.CT.QQHT
d[5,5] <- 0
d[5,6] <- d.CT.SBP
d[5,7] <- d.CT.SZJQ
d[5,8] <- d.CT.WJ
d[5,9] <- d.CT.XBCQ
d[5,10] <- d.CT.XQL
d[5,11] <- d.CT.YBBX
d[6,1] <- d.CT.DC + -d.CT.SBP
d[6,2] <- d.CT.ECSZYQ + -d.CT.SBP
d[6,3] <- d.CT.MXGS + -d.CT.SBP
d[6,4] <- d.CT.QQHT + -d.CT.SBP
d[6,5] <- -d.CT.SBP
d[6,6] <- 0
d[6,7] <- -d.CT.SBP + d.CT.SZJQ
d[6,8] <- -d.CT.SBP + d.CT.WJ
d[6,9] <- -d.CT.SBP + d.CT.XBCQ
d[6,10] <- -d.CT.SBP + d.CT.XQL
d[6,11] <- -d.CT.SBP + d.CT.YBBX
d[7,1] <- d.CT.DC + -d.CT.SZJQ
d[7,2] <- d.CT.ECSZYQ + -d.CT.SZJQ
d[7,3] <- d.CT.MXGS + -d.CT.SZJQ
d[7,4] <- d.CT.QQHT + -d.CT.SZJQ
d[7,5] <- -d.CT.SZJQ
d[7,6] <- d.CT.SBP + -d.CT.SZJQ
d[7,7] <- 0
d[7,8] <- -d.CT.SZJQ + d.CT.WJ
d[7,9] <- -d.CT.SZJQ + d.CT.XBCQ
```

```
d[7,10] <- -d.CT.SZJQ + d.CT.XQL
d[7,11] <- -d.CT.SZJQ + d.CT.YBBX
d[8,1] <- d.CT.DC + -d.CT.WJ
d[8,2] <- d.CT.ECSZYQ + -d.CT.WJ
d[8,3] <- d.CT.MXGS + -d.CT.WJ
d[8,4] <- d.CT.QQHT + -d.CT.WJ
d[8,5] <- -d.CT.WJ
d[8,6] <- d.CT.SBP + -d.CT.WJ
d[8,7] <- d.CT.SZJQ + -d.CT.WJ
d[8,8] <- 0
d[8,9] <- -d.CT.WJ + d.CT.XBCQ
d[8,10] <- -d.CT.WJ + d.CT.XQL
d[8,11] <- -d.CT.WJ + d.CT.YBBX
d[9,1] <- d.CT.DC + -d.CT.XBCQ
d[9,2] <- d.CT.ECSZYQ + -d.CT.XBCQ
d[9,3] <- d.CT.MXGS + -d.CT.XBCQ
d[9,4] <- d.CT.QQHT + -d.CT.XBCQ
d[9,5] <- -d.CT.XBCQ
d[9,6] <- d.CT.SBP + -d.CT.XBCQ
d[9,7] <- d.CT.SZJQ + -d.CT.XBCQ
d[9,8] <- d.CT.WJ + -d.CT.XBCQ
d[9,9] <- 0
d[9,10] <- -d.CT.XBCQ + d.CT.XQL
d[9,11] <- -d.CT.XBCQ + d.CT.YBBX
d[10,1] <- d.CT.DC + -d.CT.XQL
d[10,2] <- d.CT.ECSZYQ + -d.CT.XQL
d[10,3] <- d.CT.MXGS + -d.CT.XQL
d[10,4] <- d.CT.QQHT + -d.CT.XQL
d[10,5] <- -d.CT.XQL
d[10,6] <- d.CT.SBP + -d.CT.XQL
d[10,7] <- d.CT.SZJQ + -d.CT.XQL
d[10,8] <- d.CT.WJ + -d.CT.XQL
d[10,9] <- d.CT.XBCQ + -d.CT.XQL
d[10,10] <- 0
d[10,11] <- -d.CT.XQL + d.CT.YBBX
d[11,1] <- d.CT.DC + -d.CT.YBBX
d[11,2] <- d.CT.ECSZYQ + -d.CT.YBBX
d[11,3] <- d.CT.MXGS + -d.CT.YBBX
d[11,4] <- d.CT.QQHT + -d.CT.YBBX
d[11,5] <- -d.CT.YBBX
d[11,6] <- d.CT.SBP + -d.CT.YBBX
d[11,7] <- d.CT.SZJQ + -d.CT.YBBX
d[11,8] <- d.CT.WJ + -d.CT.YBBX
d[11,9] <- d.CT.XBCQ + -d.CT.YBBX
d[11,10] <- d.CT.XQL + -d.CT.YBBX
d[11,11] <- 0

# Study baseline priors
for (i in 1:ns) {
	mu[i] ~ dnorm(0, 1.694E-3)
}
```

```
        # Variance prior
        sd.d ~ dunif(0, 1.62E0)
        tau.d <- pow(sd.d, -2)

        # Effect parameter priors
        d.CT.DC ~ dnorm(0, 1.694E-3)
        d.CT.ECSZYQ ~ dnorm(0, 1.694E-3)
        d.CT.MXGS ~ dnorm(0, 1.694E-3)
        d.CT.QQHT ~ dnorm(0, 1.694E-3)
        d.CT.SBP ~ dnorm(0, 1.694E-3)
        d.CT.SZJQ ~ dnorm(0, 1.694E-3)
        d.CT.WJ ~ dnorm(0, 1.694E-3)
        d.CT.XBCQ ~ dnorm(0, 1.694E-3)
        d.CT.XQL ~ dnorm(0, 1.694E-3)
        d.CT.YBBX ~ dnorm(0, 1.694E-3)
}
```

data:

```
list(
ns = 89,
na = c(2, 2, 2, 2, 2, 2, 2, 2, 2, 2, 2, 2, 2, 2, 2, 2, 2, 2, 2, 2, 2, 2, 2, 2, 2, 2, 2, 2, 2, 2, 2, 2, 2, 2, 2, 2, 2, 2, 2, 2, 2, 2, 2, 2, 2, 2, 2, 2,
2, 2, 2, 2, 2, 2, 2, 2, 2, 2, 2, 2, 2, 2, 2, 2, 2, 2, 2, 2, 2, 2, 2, 2, 2, 2, 2, 2, 2, 2, 2, 2, 2, 2, 2, 2, 2, 2, 2, 2, 2),
t = structure(.Data = c(5, 1, 5, 1, 5, 1, 5, 1, 5, 2, 5, 2, 5, 10, 5, 10, 5, 10, 5, 10, 5, 10, 5, 10, 5, 10, 5, 10, 5, 10, 5, 10, 5, 10, 5,
10, 5, 10, 5, 10, 5, 2, 5, 10, 5, 10, 5, 6, 5, 11, 5, 11, 5, 8, 5, 1, 5, 2, 5, 7, 5, 10, 5, 2, 5, 8, 5, 10, 5, 1, 5, 3, 5, 3, 5, 8, 5, 8, 5, 6, 5, 7, 5,
7, 5, 8, 5, 10, 5, 9, 5, 3, 5, 3, 5, 3, 5, 3, 5, 3, 5, 3, 5, 3, 5, 3, 5, 3, 5, 3, 5, 2, 5, 8, 5, 4, 5, 4, 5, 4, 5, 4, 5, 4, 5, 2, 5, 2, 5, 6, 5, 6, 5, 6, 5,
6, 5, 6, 5, 6, 5, 6, 5, 7, 5, 7, 5, 7, 5, 7, 5, 7, 5, 7, 5, 7, 5, 7, 5, 7, 5, 7, 5, 7, 5, 7, 5, 7, 5, 7, 5, 8, 5, 8, 5, 8), .Dim = c(89, 2)),
m = structure(.Data = c(2.48, 2.91, 2.02, 2.76, 1.81, 2.39, 0.7, 0.9, 1.71, 2.01, 1.31, 1.73, 1.52, 1.89, 1.53, 1.9, 0.94, 1.57, 1.18,
1.28, 1.59, 1.89, 1.97, 2.21, 2.41, 2.31, 1.28, 1.48, 1.25, 1.49, 1.23, 1.48, 2.18, 2.32, 1.3, 1.46, 1.53, 1.88, 1.49, 1.85, 2.27, 2.57,
1.79, 2.02, 1.03, 2.65, 1.97, 2.61, 0.98, 1.27, 1.32, 1.43, 1.8, 2.2, 1.73, 1.72, 1.34, 1.65, 1.93, 2.52, 1.74, 2.11, 2.37, 3.01, 3.01, 3.41,
1.37, 2.89, 2.18, 3.58, 2.12, 2.5, 2.06, 2.74, 2.25, 2.91, 1.53, 2.77, 1.96, 2.78, 1.74, 1.91, 2.51, 2.89, 1.38, 1.51, 1.49, 1.85, 1.89,
2.47, 1.76, 2.01, 1.5, 1.9, 2.05, 2.65, 1.97, 2.55, 1.77, 2.51, 1.53, 1.88, 1.02, 1.14, 1.44, 1.72, 1.3, 1.84, 1.19, 1.37, 2.75, 3.42, 1.82,
2.12, 1.63, 1.87, 1.5, 1.93, 1.43, 2.02, 1.58, 1.98, 0.58, 1.05, 1.63, 2.1, 1.75, 2.13, 3.57, 4.23, 1.68, 2.29, 1.68, 2.08, 1.98, 2.46, 1.23,
1.6, 2.24, 3.69, 0.97, 1.85, 2.1, 2.34, 1.38, 1.51, 1.32, 1.56, 1.44, 1.53, 1.96, 2.65, 2.08, 2.79, 2.3, 2.79, 1.55, 1.76, 2.01, 2.38, 1.74,
2.4, 2.46, 2.84, 1.4, 1.58, 1.5, 1.64, 1.35, 1.56, 1.57, 1.86, 1.42, 1.84, 1.62, 1.86, 1.25, 1.41), .Dim = c(89, 2)),
e = structure(.Data = c(0.11155919749377254, 0.132664991614216, 0.09644946863513557, 0.13914021704740867,
0.09833333333333333, 0.04666666666666667, 0.028005601680560196, 0.04200840252084029, 0.031601109742955255,
0.03628275563080048, 0.09103897647763422, 0.13578694796664087, 0.07685277617854286, 0.07181324987175317,
0.050963686064660764, 0.05753964555687505, 0.04, 0.044, 0.04041451884327381, 0.05484827557301445,
0.0648074069840786, 0.10979897063947536, 0.04666904755831214, 0.029698484809834995, 0.10338324447460158,
0.0933762156389882, 0.023717082451262844, 0.03952847075210474, 0.059160797830996155, 0.05747048932153913,
0.03428637565499677, 0.03130495168499705, 0.05059644256269407, 0.04110960958218893, 0.018226447537315345,
0.027339671305973014, 0.08909545442950499, 0.08768124086713189, 0.025235730725761792, 0.03670651741928988,
0.02897472836319489, 0.03919647479510927, 0.06254141484991023, 0.07099295739719538, 0.05013026509266503,
0.15039079527799507, 0.05383563747248624, 0.06040095911547238, 0.06857275130999355, 0.0626099033699941,
0.03952847075210474, 0.03636619309193636, 0.06454972243679027, 0.051639777949432225, 0.048083261120685235,
0.03111269837220809, 0.009486832980505138, 0.012649110640673518, 0.04409081537009721, 0.039191835884530846,
0.03952847075210474, 0.03478505426185217, 0.06999999999999999, 0.09833333333333333, 0.08539919938625863,
0.07014934235299815, 0.02277698070958897, 0.039047482407358114, 0.175, 0.2083783541098702, 0.04472135954999579,
0.037267799624996496, 0.04763139720814413, 0.03571428571428571, 0.10922037771904522, 0.14293037084220733,
```

0.05542869676919993, 0.059804646514136764, 0.0401188709901439, 0.0324037034920393, 0.04230084753438942, 0.049074772881118195, 0.041174613989803266, 0.06404939953969396, 0.04056740422696879, 0.042257712736425826, 0.0282842712474619, 0.06505382386916238, 0.07778174593052023, 0.08061017305526641, 0.0232379000772445, 0.02065591117977289, 0.04803844614152614, 0.06324555320336758, 0.023717082451262844, 0.03478505426185217, 0.05125692857821982, 0.08795200711499172, 0.07571428571428572, 0.10857142857142857, 0.0206418738616856, 0.06340004114660576, 0.011067971810589328, 0.010801234497346435, 0.05059644256269407, 0.058502136713115015, 0.08, 0.085, 0.020816659994661327, 0.05924741690788224, 0.06350852961085883, 0.08429313930168535, 0.060083275543199206, 0.06324555320336758, 0.0616644143732834, 0.07115124735378853, 0.07981596478959654, 0.10642128638612874, 0.07358668357794092, 0.08391463916782736, 0.0670820393249937, 0.0626099033699941, 0.02745625891934577, 0.08040761540665545, 0.12004385163676429, 0.13788820796114817, 0.051639777949432225, 0.05422176684690383, 0.15628847352419098, 0.204944319055307, 0.07167432805632419, 0.06709937094634606, 0.046852128566581816, 0.04937707198786941, 0.06557438524302, 0.07167432805632419, 0.08538149682454624, 0.13123452289698773, 0.081, 0.11699999999999999, 0.017392527130926084, 0.025298221281347035, 0.0901249133147988, 0.10277402395547233, 0.03394112549695428, 0.0282842712474619, 0.05602794333886091, 0.061925621585056795, 0.01805787796286538, 0.020465595024580764, 0.052177581392778255, 0.05692099788303082, 0.09627089438861042, 0.08897764481371569, 0.14404174742066966, 0.15526783311426742, 0.05196152422706632, 0.044744645862196, 0.03320391543176798, 0.05375872022286245, 0.051241009217627885, 0.0720576692122892, 0.13293607486307094, 0.03394112549695428, 0.046437164603475266, 0.051241009217627885, 0.022135943621178655, 0.022135943621178655, 0.033166247903554, 0.028643577734887543, 0.0796486185631891, 0.08277209380096122, 0.052177581392778255, 0.07496340570653091, 0.07115124735378853, 0.07431352501395691, 0.042018058515111215, 0.05514870180108347), .Dim = c(89, 2))
)

ints1:
list(
d.CT.DC = 0.4657782498852676,
d.CT.ECSZYQ = 0.3002131549902316,
d.CT.MXGS = 0.3529568054779419,
d.CT.QQHT = 0.3112377374840448,
d.CT.SBP = 0.5453795226212584,
d.CT.SZJQ = 0.3313305582690305,
d.CT.WJ = 0.694015483877291,
d.CT.XBCQ = 0.2112564796609352,
d.CT.XQL = 0.3470167280629633,
d.CT.YBBX = 0.5125255187427272,
mu = c(2.3818943189141875, 1.870768115149672, 1.9608627837037016, 0.6390247525859614, 1.5800557984733903, 1.1280632332076577, 1.6008540695206448, 1.5074134190835622, 0.922624268698669, 1.239563888229044, 1.5027378114973478, 2.1036558969552406, 2.4548514733238336, 1.2600088908095552, 1.4077377923897263, 1.2479555766862653, 1.9299293654039218, 1.306858832437694, 1.0653657439502706, 1.4721232835691371, 2.3119695125034614, 1.8623680669891471, 1.145627997069961, 1.9973495660473137, 1.1145909212257148, 1.3852020678683932, 1.605699563700494, 1.7508679840121164, 1.3547673266331204, 1.7435557610225811, 1.9265217209167491, 2.3340668800089364, 2.9058813649622097, 1.3168524712764744, 2.1079002423514623, 1.9553009413273903, 2.051247796992843, 2.1339084072971217, 1.57951814143095, 1.8524020162472432, 1.7824273154589394, 2.4680404778035783, 1.3908922267146833, 1.5239919117955938, 1.8407151544094005, 1.7483426703658889, 1.3300670100048044, 2.0422188436396476, 2.0132474968813256, 2.012630602739931, 1.6030885693059347, 1.0233461335662248, 1.5066555982879908, 1.4506106475064267, 1.1133524349006194, 2.460671813595763, 1.8274445938632111, 1.820551170104349, 1.8312652399411902, 1.3408382859595223, 1.6958935374458497, 0.47084276415897297, 1.5347552454217626, 1.7481849150249102, 3.56299520999219, 1.8169396408588787, 1.6637556406068144, 2.010319838325392, 1.0874660967081837, 2.223405912786685, 0.9221269777689651, 2.0147369080621518, 1.4664920283412024, 1.43649427555503, 1.4282235076637542, 1.9772506050371597, 2.0401237576012226, 1.7474563275061281, 1.5282320516237442, 1.9816810166134466,

1.7313655538457504, 2.9895974492691106, 1.3096671585547128, 1.4605767630579929, 1.379514882252611, 1.9230959278779411, 1.651662237144528, 1.7053645072942183, 1.3903328585675763),
delta = structure(.Data = c(NA, 0.6361611947468091, NA, 1.1183465468376421, NA, 0.33536822795076004, NA, 0.4361273886430812, NA, 0.2686725555576458, NA, 0.25577727177439347, NA, 0.10670085745296204, NA, 0.3894252537720172, NA, 0.7611899032558204, NA, 0.3374296464538501, NA, 0.4996779586905855, NA, 0.2142683739702906, NA, 0.39399896217756664, NA, 0.1304778235277688, NA, 0.013704447190309554, NA, 0.20875690620836432, NA, -0.3785471153098705, NA, 0.2219657471950819, NA, 0.4753734010698172, NA, 0.2750234693260513, NA, 0.3224922682080117, NA, 0.6029641504419612, NA, 2.1210116811767623, NA, 0.37335449781658814, NA, 0.12110768465417851, NA, 0.14872597677306598, NA, 0.21411989083849411, NA, 0.02846378776733366, NA, 0.3352282075377949, NA, 0.6421598775919821, NA, 0.319213574297453, NA, 0.49278441132824047, NA, 0.35506683873413897, NA, 1.5841182783796228, NA, 1.8921881797944762, NA, 0.4173421544449261, NA, 0.5407967742015799, NA, 0.5521742854280728, NA, 1.2363932672085602, NA, 0.9418342893167132, NA, 0.07092082943371641, NA, 0.4145488663790378, NA, 0.1633922881291103, NA, 0.4291733590179853, NA, 0.8847876649962996, NA, 0.27331191095760016, NA, 0.3402524030031566, NA, 0.6454253584494475, NA, 0.3682742796010763, NA, 0.3680300548597721, NA, 0.1989582337391186, NA, 0.17250555755504993, NA, 0.49727119724126884, NA, 0.3007611947384505, NA, 0.38710990649756405, NA, 0.46038637565730467, NA, 0.5563625436899209, NA, -0.17965996038756654, NA, -0.31202722281857076, NA, 0.9791386484269525, NA, 0.29270418759748096, NA, 0.7373718974882073, NA, 0.5156351454906846, NA, -0.019102140690603397, NA, 0.2944587876117374, NA, 0.5912506589199384, NA, 0.48159146994419666, NA, 0.06587299535905977, NA, -0.03651915059499422, NA, 1.4348845120742295, NA, 0.9013719126292, NA, 0.7746868936482255, NA, 0.26922098590702054, NA, 0.1503744131115743, NA, -0.08914944411582895, NA, 0.6283774375516252, NA, 0.8002709370930436, NA, 0.9635390190385513, NA, 0.1912696201057515, NA, 0.1798509415365237, NA, 0.5327622305082469, NA, 0.12095192719134712, NA, 0.21878303696014262, NA, 0.2046653770157797, NA, 0.3895086918630891, NA, 0.5103214400936422, NA, 0.2728922256738596, NA, 0.17987616478198043, NA, 0.22278322592854038), .Dim = c(89, 2)),
sd.d = 0.9780321235187649
)

ints2:
list(
d.CT.DC = 0.3094496787012458,
d.CT.ECSZYQ = 0.0448176074795511,
d.CT.MXGS = 0.5053726541680928,
d.CT.QQHT = 0.6586128078210975,
d.CT.SBP = 0.549735659129423,
d.CT.SZJQ = 0.22438308968975354,
d.CT.WJ = -0.6804428639499578,
d.CT.XBCQ = 0.30748905698481144,
d.CT.XQL = 0.38642633548123656,
d.CT.YBBX = 0.27106673042639906,
mu = c(2.385536030312519, 1.7930707632671263, 1.7150600406453385, 0.8236545530059999, 1.6702242068355513, 1.7068057486761758, 1.2193872380149196, 1.4025627900358286, 0.8089506476580834, 1.300253143329922, 1.4169525267258014, 1.7136676981043597, 2.6527978273159203, 1.2987968841642035, 1.1157752407319215, 1.2427269118851023, 1.8971883552471578, 1.4035993999919962, 1.323946544946946, 1.5763201103558129, 2.306119573182155, 1.6634830757770742, 1.0628888517214259, 1.8698551767283635, 0.9307587716783983, 1.2100502841696097, 1.59237996679746, 1.5022063720960046, 1.321302678567349, 1.895112688847031, 1.6920556107815579, 2.1830880844810405, 2.931770836522446, 1.3605284654284262, 1.8367677388280805, 2.001485709326731, 1.9287652679742864, 2.0553856067547884, 1.3765509391137696, 2.047222366419076, 1.6972029206236388, 2.5383516978657155, 1.2945711837019278, 1.4655634519629142, 2.233883780518199, 1.7214033804476019, 1.3513604840259692, 1.9523750919127407, 1.9668130967999418, 1.8501865246314784, 1.6102962863239068, 1.0447610370396763, 1.6548348223625842, 1.1915256157138376, 1.1647110800103861,

2.9495263986201157, 1.7558344175445069, 1.7856064127221702, 1.1852324793986557, 1.7840457805286036, 1.5975510397625265, 0.7185467090812994, 2.11309886478286, 1.8632592169559117, 3.2870753679704707, 1.7381092273645335, 1.6572402342305999, 1.8227631139124254, 1.1571125790135108, 2.0713711768730354, 1.010643024532436, 2.095875620301145, 1.5040522402853398, 1.1467615965591196, 1.4449277732949335, 2.0847076906036732, 2.164782264664229, 2.614214813535976, 1.4108904252669778, 1.965528935774734, 1.756462685176489, 3.2935493871113732, 1.5479755025699768, 1.4365834631616812, 1.316650091970546, 1.7880033534825963, 1.476913057979948, 1.5931331421525743, 1.2426489255593187),
delta = structure(.Data = c(NA, -0.13275951056814328, NA, 0.3645278160249455, NA, 0.1583267574322828, NA, 0.12423511408870606, NA, 0.15478052176905605, NA, -0.11143936286475653, NA, 0.9539420512999691, NA, 0.4866833085178103, NA, 0.5827584537752655, NA, 0.0807887483998434, NA, 0.4055496872102765, NA, 0.31645723520011704, NA, -0.41709326418331927, NA, 0.1691069932265721, NA, 0.47434197479347884, NA, 0.40091284300987495, NA, 0.13977771068753683, NA, 0.05213571235567234, NA, 0.9578284071278397, NA, 0.37413730606031265, NA, 0.4521331571673659, NA, 0.29072310019245196, NA, 1.6597763286913274, NA, 0.3954770070259491, NA, 0.2560162778372418, NA, 0.08784617566866385, NA, 0.19811880538449933, NA, -0.13742329610093412, NA, 0.2576335434925625, NA, 0.3005024527657093, NA, 0.345493710876284, NA, 1.1727176589791073, NA, 0.7928284397406749, NA, 1.4652611937423765, NA, 0.6009142110677332, NA, 0.3287792813258647, NA, 0.8388610121001517, NA, 1.4480904989335472, NA, 1.3853352184988204, NA, 0.9287723764957061, NA, 0.3066049752520926, NA, 0.22133791009733025, NA, 0.09892674761586137, NA, 0.2734191329550447, NA, 0.7213765858700494, NA, 0.23225595160974963, NA, 0.8229200996393766, NA, 0.6385129849367501, NA, 0.35196543338347797, NA, 0.9161663348797454, NA, 0.28479214961640387, NA, 0.1335258276820577, NA, 0.42498203582082994, NA, 0.4639447156576927, NA, 0.10935052003469804, NA, 0.6834429896796056, NA, 0.4878402236383244, NA, 0.3932916363441233, NA, -0.10720406469940658, NA, 0.9471050739578089, NA, 0.2281236145707431, NA, 0.36488131503243904, NA, 0.1978694239585187, NA, 0.14800418036108784, NA, 1.1992447739797707, NA, 0.641289340620445, NA, 0.23729423023123028, NA, 0.8640883591700532, NA, 0.27652530485943677, NA, 0.31956846265623984, NA, 0.8840313550544348, NA, 0.3021080421833202, NA, 0.04706060136739226, NA, 0.46200941777856896, NA, 0.131174864247941, NA, 0.7107283229132315, NA, 1.163072555417782, NA, 0.1881501980038463, NA, 0.24494545080158442, NA, -0.10179560002254184, NA, 0.791563897351882, NA, -0.08325136398783389, NA, 0.264695352970516, NA, 0.13629029579654903, NA, 0.28958722549293825, NA, 0.1379789218499909, NA, -0.01922108451543686, NA, 0.24035579245549493, NA, -0.13240056344487078), .Dim = c(89, 2)),
sd.d = 0.09740479092849078
)

ints3:
list(
d.CT.DC = 0.155301808816027,
d.CT.ECSZYQ = 0.3280157656402745,
d.CT.MXGS = 0.5837976333396927,
d.CT.QQHT = 0.41819526818033403,
d.CT.SBP = 0.6833815704002434,
d.CT.SZJQ = 0.284504849467619,
d.CT.WJ = 1.3879021841748052,
d.CT.XBCQ = 0.07606825782464843,
d.CT.XQL = 0.3166467739784864,
d.CT.YBBX = 0.6356589074306886,
mu = c(2.336763494255897, 2.249095133531146, 2.070002125291421, 0.8099884839501627, 1.605282826606936, 1.154967739347709, 1.6297223689135565, 1.4863721774937948, 0.8648431842890879, 1.1864438236003672, 1.5725960782330048, 1.9024651224032598, 2.4821983212311296, 1.3308224672340567, 1.2741634347144968, 1.2764853300814667, 2.206026354282791, 1.3486381880599245, 1.264633841656989, 1.4834392980683244, 2.182285728839637, 1.6547689684902835, 0.9432272062248457, 1.937747403280735, 1.2051114441157673, 1.3750189958273498, 1.6061275906142924, 1.7005121010879944, 1.322627227669802, 1.8200240144261963,

1.7979552345591436, 2.342325073133636, 2.9255936932193243, 1.4370069990012544, 2.666027961397986, 2.148949347882312, 2.1123123830653787, 1.9566816378085734, 1.6137433918076776, 1.7458498178715107, 1.7189467768309048, 2.4447426456749177, 1.2479030024014426, 1.5397103465251702, 1.6783885167117505, 1.84577391459934, 1.395310795967306, 2.103702113003199, 2.0114149316211147, 1.736067775601865, 1.4767560493664398, 1.014047120984332, 1.2541873924173512, 1.3045117326370563, 1.1731070045824763, 2.768504963475198, 1.8654205585039838, 1.6980997244005214, 1.3966107206907294, 1.2832573871190305, 1.4844749919188551, 0.4158211700830976, 1.6101468840709314, 1.6892965873771184, 3.039773081521402, 1.7263640710500179, 1.589166658210611, 2.293110821556261, 1.2322634325847355, 2.284259619070489, 1.1216320912100133, 2.520169993418884, 1.3329712918704337, 1.5349671025651919, 1.4279678072632551, 1.7333043213324166, 1.7806927929604384, 2.796793623588461, 1.5437931547041057, 1.8798539222882225, 1.6196844930366954, 2.1259675677641505, 1.3392658473208616, 1.491878046874434, 1.425991069243645, 1.3612555151530314, 1.3550029460562434, 1.7905801325425712, 1.1341596291349854),
delta = structure(.Data = c(NA, 0.8855153510457915, NA, 0.6925653056804557, NA, 0.8537656845276239, NA, 0.2611738069382496, NA, 0.02736043693686574, NA, 0.09742579434324944, NA, 0.15684523016901666, NA, 0.5126431089892519, NA, 0.6604709461487249, NA, 0.2921637771221849, NA, -0.15113670924315792, NA, 0.13049058970456398, NA, -0.3777604774117876, NA, 0.15703438117109247, NA, 0.5284109671204682, NA, 0.2216654876233402, NA, 0.5992034233409512, NA, 0.24872858703356676, NA, 0.3389717873324671, NA, 0.44724289260785965, NA, 0.4861413707171681, NA, 0.317730234477292, NA, 2.11020323397694, NA, 0.7091254211628129, NA, 0.34184666406717745, NA, 0.3833614366932879, NA, 0.5267887021991564, NA, 0.05572767682258617, NA, 0.23502323526180352, NA, 0.30641130593586824, NA, 0.4089102533535669, NA, 0.9639122242773192, NA, 0.5734904473118418, NA, 1.5027536417423184, NA, 1.2429843720810436, NA, 0.3676697681934971, NA, 0.6636983619073433, NA, 1.0275309496570126, NA, 0.8416770983647197, NA, 0.5802657205828541, NA, 0.3472280605347424, NA, 0.3159896631489835, NA, -0.09424323689604122, NA, 0.2985370102542433, NA, 0.10110362908787618, NA, 0.1585087962088919, NA, 0.35390347653925186, NA, 0.7167566453001628, NA, 0.7907808864729875, NA, 0.8846341507801819, NA, 0.34999619902139206, NA, 0.11458560462975743, NA, 0.2647305013276328, NA, 0.8891647343308056, NA, 0.18724525958467383, NA, 0.9485157657088445, NA, 0.4467778150544422, NA, 0.11781561140625066, NA, 0.440194677814738, NA, 0.4774167683533034, NA, 0.1686793966402962, NA, 0.38937803072820054, NA, 0.2659089108277404, NA, 0.6787226269985924, NA, 2.002438731563073, NA, 0.5497801349448239, NA, 0.41389805720671946, NA, 0.6218323852520442, NA, 0.7783443480692004, NA, 1.1224022317165323, NA, 1.003688496038017, NA, 0.6669953560137798, NA, -0.025817097627396562, NA, 0.14332866603407435, NA, 0.1043447430663623, NA, 1.098861639715618, NA, 1.0526768568475704, NA, -0.02624215927835316, NA, 0.4682877872927251, NA, 0.1182890701669907, NA, 0.6541398017994072, NA, 0.14748685425413366, NA, 0.35022149488670457, NA, 0.019995104333641073, NA, 0.19146387404349655, NA, 0.14021145469349244, NA, 0.4498567518669253, NA, 0.43381571656828877, NA, 0.14374625445375916), .Dim = c(89, 2)),
sd.d = 0.6076957476486425
)

ints4:
list(
d.CT.DC = 0.34662039099629627,
d.CT.ECSZYQ = 0.3739986477396033,
d.CT.MXGS = 0.40214291210290076,
d.CT.QQHT = 0.4877130167589763,
d.CT.SBP = 0.34732682173709095,
d.CT.SZJQ = 0.17235461195819216,
d.CT.WJ = 1.3392706470281603,
d.CT.XBCQ = 0.40307826779924105,
d.CT.XQL = 0.27591168887870116,
d.CT.YBBX = 0.15756882757566565,

mu = c(2.7583670231470503, 1.983315254338271, 1.692407120951206, 0.6158716697553238, 1.8197963437510547, 1.151571235165827, 1.4109198980715134, 1.4377538665440641, 1.0295099558400327, 1.2393106787546098, 1.4619171927480887, 1.8938808451902918, 2.1715753726337192, 1.2586795777576778, 1.155095747989843, 1.2342612546234848, 2.3576769224599237, 1.3070598993981837, 1.1888396935039633, 1.4404151495730384, 2.1888753091192323, 1.7642388569378917, 1.0372632604017127, 1.7842254287960952, 0.727891214316484, 1.2968389285390263, 1.6597504256773656, 1.638923796210107, 1.3503858556717532, 2.0006745083361674, 1.70259484700533, 2.285670148931208, 3.194809105566I405, 1.387122861119717, 2.358984407193668, 2.2544340904146125, 2.1132315672592243, 1.9825923755802406, 1.5418101224631655, 1.9211427997636648, 1.8562690904263812, 2.256720752026681, 1.4091561428376413, 1.5523504213027588, 1.9773685436109623, 1.7698529662223357, 1.4086283137357711, 2.0217211493385947, 1.9069714226468877, 1.7326856080633726, 1.5008212478622054, 0.9400419192312216, 1.3399846618555393, 1.602416119682447, 1.105934386866521, 2.661283012230096, 1.6877537996150815, 1.6155337343622107, 1.4282839649344363, 1.6644418391986457, 1.7297540820077069, 0.6840263618149681, 1.520636357541646, 1.7066242083538825, 3.2095102045673607, 1.592521398616895, 1.8043830448225955, 1.8312700570695728, 1.1193611554404976, 1.8864618240669093, 0.9907572861119763, 2.2222060487284936, 1.227233429776046, 1.401566618596072, 1.4851202198654814, 1.7242195671187777, 1.8401794122580706, 2.4459839175112372, 1.4978732663827217, 2.02775237449652, 1.606424172435037, 1.8711460014673085, 1.5740393450442876, 1.5264799360063868, 1.367727242058334, 1.5290409129515614, 1.295928075489177, 1.6113100966126415, 1.1152991280547677),
delta = structure(.Data = c(NA, 0.0755479312581605, NA, 0.2396354631948725, NA, 0.5905822023351268, NA, 0.02131073092495031, NA, 0.021092817028736888, NA, 0.6795917693370015, NA, 0.5333739088481515, NA, 0.38958769991315595, NA, 0.7094214574722851, NA, 0.5753866855546682, NA, 0.17203078932467314, NA, 0.10374192077748362, NA, -0.23309693285529373, NA, 0.028387005193233755, NA, 0.11995760113813093, NA, 0.14085969551239536, NA, 0.2573686428731564, NA, 0.09501501180238128, NA, 0.25249416642812417, NA, 0.5050667213985003, NA, 0.42626930593686263, NA, -0.05460995785891032, NA, 2.0747458366590465, NA, 0.6112994824364958, NA, 0.2522290046210264, NA, 0.22656049163481545, NA, 0.3302735574577246, NA, 0.10256249623142417, NA, 0.2616449129766567, NA, 0.5794117253935259, NA, 0.48147431680561603, NA, 0.7527992793037198, NA, 0.5004058883394702, NA, 1.630662751483457, NA, 1.1762773192050893, NA, 0.21015945754553778, NA, 0.7435646573326145, NA, 0.8845303920999631, NA, 1.1007068709671421, NA, 0.791753241200636, NA, 0.25510013511004903, NA, 0.007480551341565778, NA, 0.2006676928872151, NA, 0.3351365673345485, NA, 0.23323235912327978, NA, 0.23786115434384056, NA, 0.4037519919095681, NA, 0.6358689154540584, NA, 0.4068651695368285, NA, 1.221779921175851, NA, 0.3673708179064212, NA, 0.14143646341042276, NA, 0.2318124193592119, NA, 0.4752693212618374, NA, 0.09178574598434278, NA, 0.987124128846643, NA, 0.046362657050727174, NA, 0.12086103209167977, NA, 0.086001197343204, NA, 0.6229184794037573, NA, 0.2187122163404606, NA, 0.5457220257338942, NA, 0.21938245832249054, NA, 0.6017572253218758, NA, -0.45687596490466365, NA, 0.9181696038409419, NA, 0.45739519325694156, NA, 0.4978426440962267, NA, 0.34363879827782584, NA, 1.1225884194279414, NA, 0.8662104750658972, NA, 0.8634149498986152, NA, 0.27293801216806723, NA, 0.4418320627041713, NA, 0.10885165371576319, NA, 0.8620396949035435, NA, 0.9490762882933172, NA, 0.7463899139487239, NA, 0.1370325422390818, NA, 0.4589795770844629, NA, 0.4435043385853081, NA, 0.2950987853167219, NA, 0.15929874664213578, NA, 0.2651588647125921, NA, 0.31359395746437424, NA, 0.2945554957574039, NA, 0.7753717399457507, NA, 0.3819261710296165, NA, 0.40446340073378156), .Dim = c(89, 2)),
sd.d = 0.4603767621589299
)

Appendix Fig A.1 Bias risk assessment of included studies

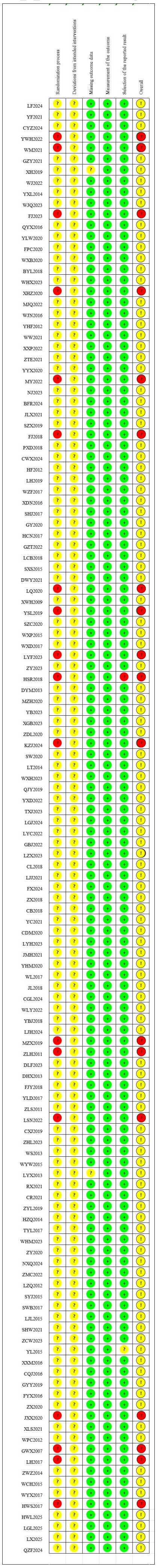

Appendix Fig A.2–A.9 Meta-regression analysis

Appendix Fig A.2 Overall risk of bias

	2.5%	25%	50%	75%	97.5%
d.11.1	-0.3053	0.25283	0.47050	6.886e-01	1.2743
d.11.10	0.1765	0.32767	0.40553	4.837e-01	0.6349
d.11.2	0.2939	0.41365	0.47442	5.360e-01	0.6575
d.11.3	0.2629	0.35635	0.40477	4.535e-01	0.5488
d.11.4	-1.1077	-0.06381	0.25131	5.685e-01	1.7272
d.11.5	-0.7852	0.24506	0.51334	8.493e-01	3.2068
d.11.6	0.3616	0.51793	0.59764	6.778e-01	0.8327
d.11.7	-1.0119	-0.05821	0.30090	6.775e-01	1.8047
d.11.8	0.3317	0.47108	0.54275	6.150e-01	0.7555
d.11.9	0.1680	0.26872	0.32059	3.726e-01	0.4738
sd.d	0.2532	0.28438	0.30307	3.233e-01	0.3678
beta[1]	-4.3268	-0.89754	0.27409	1.496e+00	5.2299
beta[2]	-0.4988	-0.24354	-0.11098	2.015e-02	0.2667
beta[4]	-0.3146	-0.06531	0.06312	1.934e-01	0.4463
beta[5]	-0.4237	-0.19467	-0.07875	4.010e-02	0.2842
beta[6]	-8.4391	-1.57839	-0.02113	1.588e+00	9.0347
beta[7]	-7.8382	-1.22121	0.21545	2.170e+00	17.4294
beta[8]	-0.3435	-0.17670	-0.08755	-1.229e-05	0.1706
beta[9]	-7.4948	-1.31972	0.24543	2.009e+00	9.3157
beta[10]	-0.5826	-0.31926	-0.18407	-4.977e-02	0.2034
beta[11]	-0.4724	-0.23092	-0.10621	9.740e-03	0.2345

```
-- Model fit (residual deviance):

    Dbar        pD       DIC
181.0119 171.1769 352.1888

178 data points, ratio 1.017, I^2 = 2%
```

Appendix Fig A.3 Sample size

	2.5%	25%	50%	75%	97.5%
d.11.1	0.04457	0.239218	0.33923	0.43940	0.6342
d.11.10	0.14757	0.294277	0.37017	0.44727	0.5961
d.11.2	0.33313	0.454039	0.51640	0.57879	0.7004
d.11.3	0.27367	0.359687	0.40433	0.44947	0.5379
d.11.4	-0.25565	0.043391	0.19570	0.34700	0.6435
d.11.5	0.19619	0.379657	0.47449	0.56847	0.7510
d.11.6	0.36292	0.489659	0.55407	0.61948	0.7460
d.11.7	-2.32590	-0.331934	0.18486	0.63661	1.9599
d.11.8	0.12587	0.307244	0.40052	0.49428	0.6762
d.11.9	0.18188	0.273324	0.32112	0.36904	0.4626
sd.d	0.23064	0.260794	0.27862	0.29817	0.3405
beta[1]	-1.69627	-0.950232	-0.55837	-0.17924	0.5560
beta[2]	-0.12544	0.002901	0.07022	0.13974	0.2725
beta[4]	-0.22386	0.143096	0.33846	0.53114	0.9071
beta[5]	-0.25465	-0.065655	0.03292	0.13151	0.3249
beta[6]	-1.30179	-0.100261	0.50983	1.15750	2.5539
beta[7]	-0.37828	-0.136939	-0.01228	0.11088	0.3500
beta[8]	0.22557	0.539949	0.70472	0.86560	1.1752
beta[9]	-5.94118	-1.130541	0.17420	1.82840	9.2089
beta[10]	-1.97813	-1.193797	-0.80660	-0.43623	0.3027
beta[11]	-0.40711	-0.199268	-0.09300	0.01403	0.2253

```
-- Model fit (residual deviance):

    Dbar        pD       DIC
182.5197 170.3760 352.8958

178 data points, ratio 1.025, I^2 = 3%
```

Appendix Fig A.4 Composition of the CT regimen

```
              2.5%      25%        50%     75%   97.5%
d.11.1      0.1617  0.33664  0.4272148 0.51822  0.6958
d.11.10     0.1610  0.32495  0.4096887 0.49455  0.6605
d.11.2      0.2697  0.39786  0.4639976 0.53074  0.6602
d.11.3      0.2509  0.34794  0.3979757 0.44792  0.5463
d.11.4     -3.2558 -0.40372  0.2480287 0.88066  4.9789
d.11.5      0.1780  0.38636  0.4935340 0.60010  0.8072
d.11.6      0.2770  0.45587  0.5469229 0.63755  0.8138
d.11.7     -3.7897 -1.27965  0.0678594 1.50183  5.6761
d.11.8      0.3127  0.46184  0.5381826 0.61568  0.7647
d.11.9      0.1692  0.27644  0.3328296 0.38912  0.4981
sd.d        0.2592  0.29135  0.3104367 0.33102  0.3762
beta[1]    -0.6816 -0.21882  0.0245445 0.26831  0.7514
beta[2]    -0.5414 -0.22895 -0.0685529 0.09338  0.4190
beta[4]    -0.4914 -0.21980 -0.0722196 0.07143  0.3475
beta[5]    -0.1478  0.05524  0.1581668 0.26256  0.4634
beta[6]   -10.1314 -1.77343 -0.0142042 1.69928 13.6549
beta[7]    -0.5625 -0.09532  0.1482687 0.39189  0.8685
beta[8]    -0.6216 -0.24005 -0.0450444 0.14944  0.5130
beta[9]    -8.5123 -1.91366  0.2950182 2.40715  6.2443
beta[10]   -0.4146 -0.14008 -0.0009907 0.13712  0.4030
beta[11]   -0.3095 -0.12272 -0.0328342 0.05504  0.2289

-- Model fit (residual deviance):

    Dbar       pD      DIC
180.4918 171.6885 352.1803

178 data points, ratio 1.014, I^2 = 2%
```

Appendix Fig A.5 Treatment duration

```
               2.5%      25%       50%      75%   97.5%
d.11.1      0.11058  0.30618  0.408671  0.51117  0.7120
d.11.10     0.01052  0.29938  0.391632  0.48308  0.7014
d.11.2      0.32884  0.44470  0.504166  0.56417  0.6813
d.11.3      0.27268  0.36025  0.405852  0.45243  0.5431
d.11.4    -13.16827 -5.80833 -1.028225  0.89149  7.9398
d.11.5     -0.01975  0.35797  0.473163  0.58923  1.0992
d.11.6      0.34493  0.49178  0.567198  0.64326  0.7911
d.11.7     -6.57663 -1.37874  0.748944  3.18943  6.0670
d.11.8      0.16756  0.31160  0.386744  0.46163  0.6075
d.11.9      0.18032  0.27606  0.325260  0.37500  0.4714
sd.d        0.23535  0.26492  0.282363  0.30150  0.3428
beta[1]    -0.46171 -0.12934  0.045017  0.21543  0.5316
beta[2]   -10.42991 -1.37865  0.085125  1.66853 14.7898
beta[4]    -0.59028 -0.37206 -0.260228 -0.12763  0.1378
beta[5]    -0.25183 -0.08810 -0.001752  0.08423  0.2540
beta[6]   -13.73937 -6.19401 -1.271684  0.64909  7.8779
beta[7]   -26.76354 -1.65784 -0.022872  1.55702 19.7991
beta[8]    -0.48546 -0.17709 -0.026445  0.12451  0.4228
beta[9]    -5.62350 -2.86228 -0.480542  1.60913  6.6231
beta[10]   -1.21679 -0.90543 -0.750053 -0.59471 -0.2671
beta[11]   -0.28107 -0.08649  0.014607  0.11413  0.3033

-- Model fit (residual deviance):

    Dbar       pD      DIC
181.0211 170.3417 351.3629

178 data points, ratio 1.017, I^2 = 2%
```

Appendix Fig A.6 Adherence to standard processing of core herbs

```
               2.5%      25%      50%       75%  97.5%
d.11.1       0.1550  0.32959  0.41952  0.510458 0.6877
d.11.10      0.1514  0.31213  0.39526  0.478456 0.6394
d.11.2       0.2821  0.40407  0.46712  0.530233 0.6537
d.11.3       0.2677  0.38370  0.44393  0.504480 0.6225
d.11.4      -0.1703  0.15127  0.31656  0.481655 0.7999
d.11.5       0.1729  0.36782  0.46780  0.569034 0.7645
d.11.6      -1.0901  0.20389  0.60445  1.026145 1.9681
d.11.7      -1.6293 -0.33725  0.15497  0.575319 1.8689
d.11.8      -1.1729  0.06495  0.49031  0.987270 2.3174
d.11.9       0.1364  0.24615  0.30228  0.358602 0.4684
sd.d         0.2557  0.28711  0.30610  0.326820 0.3717
beta[1]     -0.4200 -0.07808  0.06898  0.225885 0.5408
beta[2]     -0.5950 -0.21324 -0.01410  0.183006 0.5503
beta[4]     -0.2473 -0.02311  0.09257  0.209043 0.4283
beta[5]     -0.3399 -0.17232 -0.08704  0.003069 0.1750
beta[6]     -0.9232 -0.46831 -0.22757  0.012033 0.5155
beta[7]     -0.6733 -0.25579 -0.04375  0.170126 0.5882
beta[8]     -5.6038 -1.17396  0.14627  1.575429 4.7374
beta[9]     -6.0280 -1.68507 -0.23350  0.816235 5.3006
beta[10]    -5.8236 -1.58291 -0.16702  1.501157 6.0111
beta[11]    -0.5614 -0.28641 -0.15061 -0.010826 0.2594

-- Model fit (residual deviance):

    Dbar       pD      DIC
180.9469 171.2910 352.2379

178 data points, ratio 1.017, I^2 = 2%
```

Appendix Fig A.7 Herbal formula modification

```
               2.5%       25%      50%     75%   97.5%
d.11.1       0.1675  0.342178  0.43207 0.52261  0.6987
d.11.10      0.1261  0.298292  0.38720 0.47654  0.6475
d.11.2       0.2780  0.404938  0.47021 0.53532  0.6621
d.11.3       0.2679  0.362546  0.41173 0.46100  0.5573
d.11.4      -0.4997 -0.083805  0.12769 0.33851  0.7404
d.11.5       0.1714  0.368438  0.47022 0.57188  0.7730
d.11.6      -1.7110  0.119401  0.79666 2.04733  5.9223
d.11.7      -0.5763  0.001958  0.24295 0.48015  0.9908
d.11.8       0.3266  0.469392  0.54320 0.61622  0.7593
d.11.9       0.1334  0.250058  0.31089 0.37081  0.4886
sd.d         0.2587  0.290659  0.30953 0.33006  0.3754
beta[1]     -0.3416  0.037086  0.22594 0.40725  0.7420
beta[2]     -0.6131 -0.239951 -0.04888 0.13667  0.5019
beta[4]     -0.2953 -0.085949  0.02681 0.14133  0.3586
beta[5]     -0.2054 -0.014953  0.08477 0.18589  0.3748
beta[6]     -1.8095 -0.859300 -0.39185 0.07425  0.9366
beta[7]     -0.6224 -0.204229  0.02072 0.24193  0.6625
beta[8]     -9.8982 -2.679960 -0.43859 0.80189  4.1914
beta[9]    -18.7930 -1.726751 -0.03845 1.56899 14.2044
beta[10]    -0.2503  0.008510  0.14097 0.27307  0.5221
beta[11]    -0.4445 -0.191136 -0.06342 0.06491  0.3035

-- Model fit (residual deviance):

    Dbar       pD      DIC
180.3186 171.5334 351.8520

178 data points, ratio 1.013, I^2 = 2%
```

Appendix Fig A.8 Disease duration

```
                 2.5%      25%       50%      75%   97.5%
d.11.1        0.11058  0.30618  0.408671  0.51117  0.7120
d.11.10       0.01052  0.29938  0.391632  0.48308  0.7014
d.11.2        0.32884  0.44470  0.504166  0.56417  0.6813
d.11.3        0.27268  0.36025  0.405852  0.45243  0.5431
d.11.4      -13.16827 -5.80833 -1.028225  0.89149  7.9398
d.11.5       -0.01975  0.35797  0.473163  0.58923  1.0992
d.11.6        0.34493  0.49178  0.567198  0.64326  0.7911
d.11.7       -6.57663 -1.37874  0.748944  3.18943  6.0670
d.11.8        0.16756  0.31160  0.386744  0.46163  0.6075
d.11.9        0.18032  0.27606  0.325260  0.37500  0.4714
sd.d          0.23535  0.26492  0.282363  0.30150  0.3428
beta[1]      -0.46171 -0.12934  0.045017  0.21543  0.5316
beta[2]     -10.42991 -1.37865  0.085125  1.66853 14.7898
beta[4]      -0.59028 -0.37206 -0.260228 -0.12763  0.1378
beta[5]      -0.25183 -0.08810 -0.001752  0.08423  0.2540
beta[6]     -13.73937 -6.19401 -1.271684  0.64909  7.8779
beta[7]     -26.76354 -1.65784 -0.022872  1.55702 19.7991
beta[8]      -0.48546 -0.17709 -0.026445  0.12451  0.4228
beta[9]      -5.62350 -2.86228 -0.480542  1.60913  6.6231
beta[10]     -1.21679 -0.90543 -0.750053 -0.59471 -0.2671
beta[11]     -0.28107 -0.08649  0.014607  0.11413  0.3033

-- Model fit (residual deviance):

    Dbar       pD      DIC
181.0211 170.3417 351.3629

178 data points, ratio 1.017, I^2 = 2%
```

Appendix Fig A.9–A.14 Funnel plots

Appendix Fig A.9 FEV_1

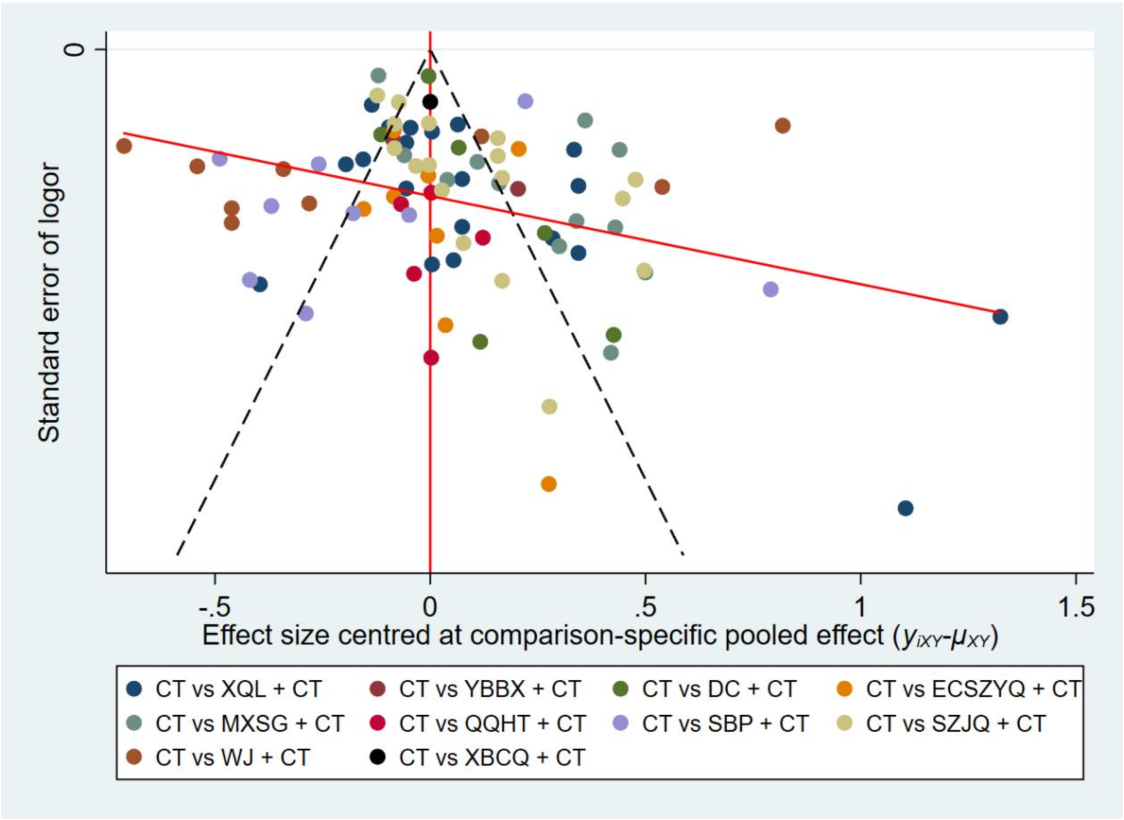

Appendix Fig A.10 FEV_1%pred

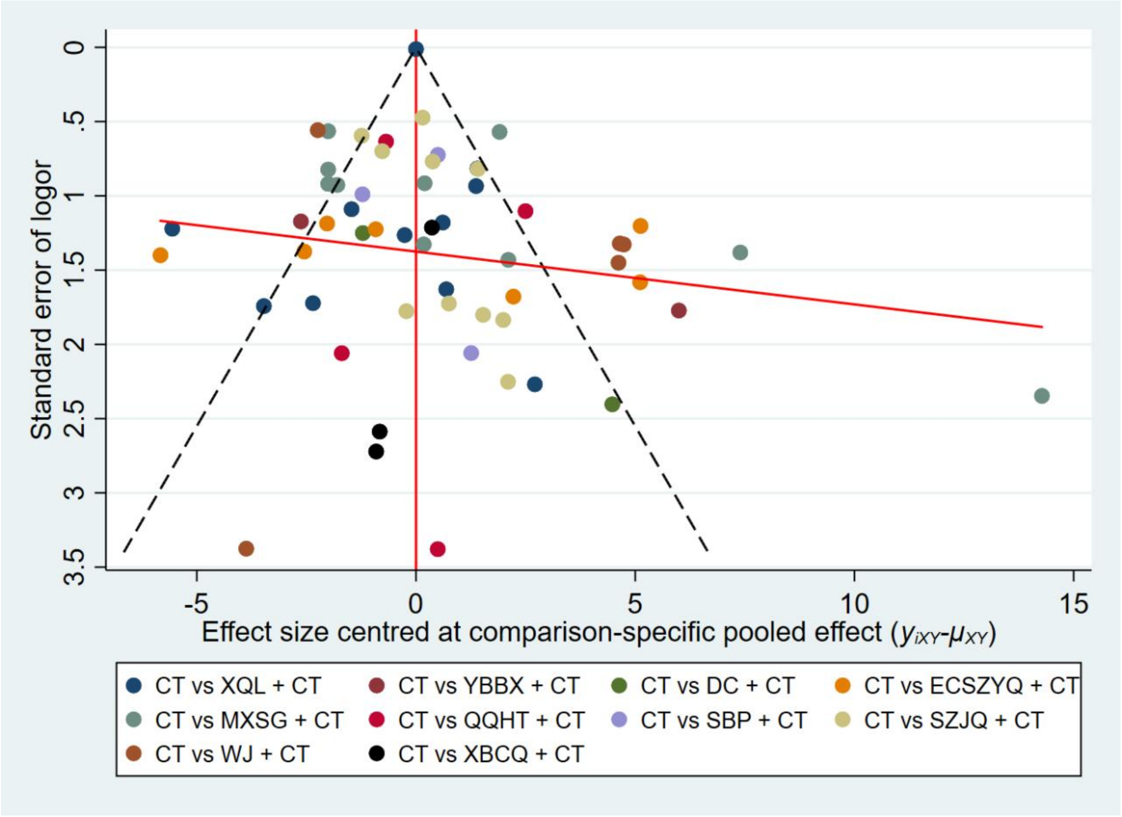

Appendix Fig A.11 FEV_1/FVC

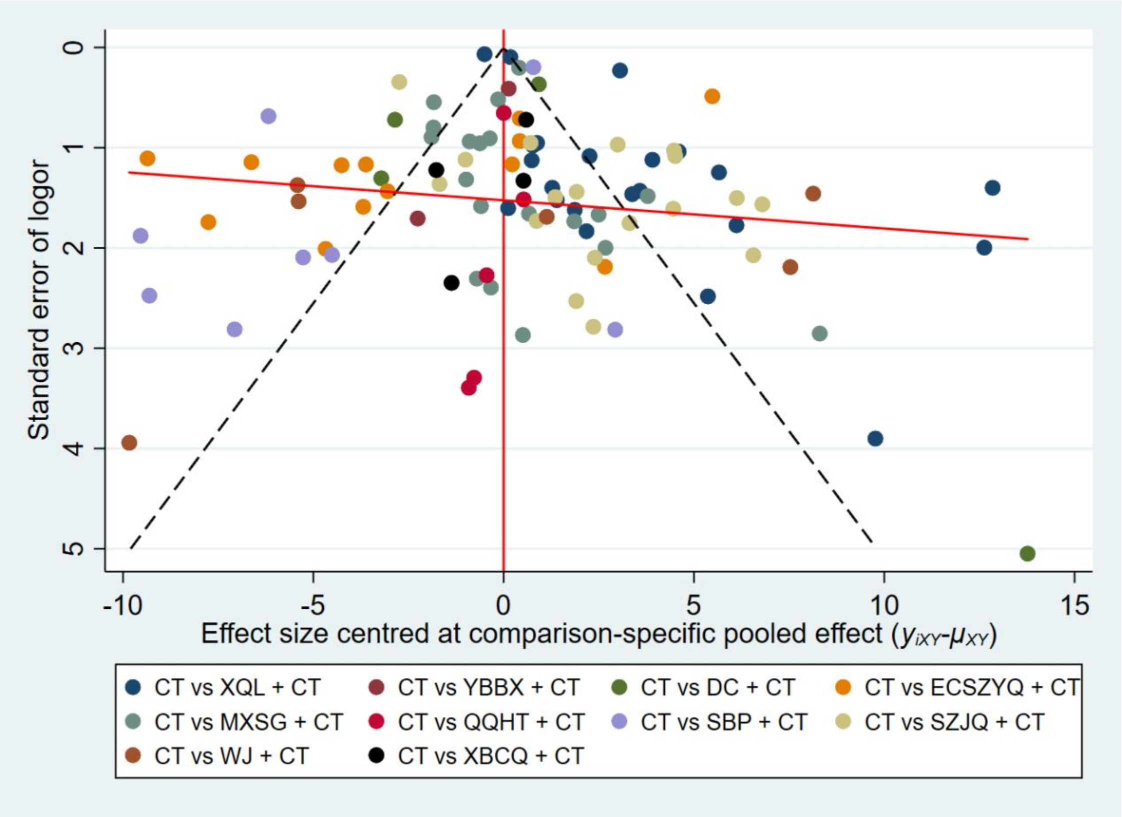

Appendix Fig A.12 PaO_2

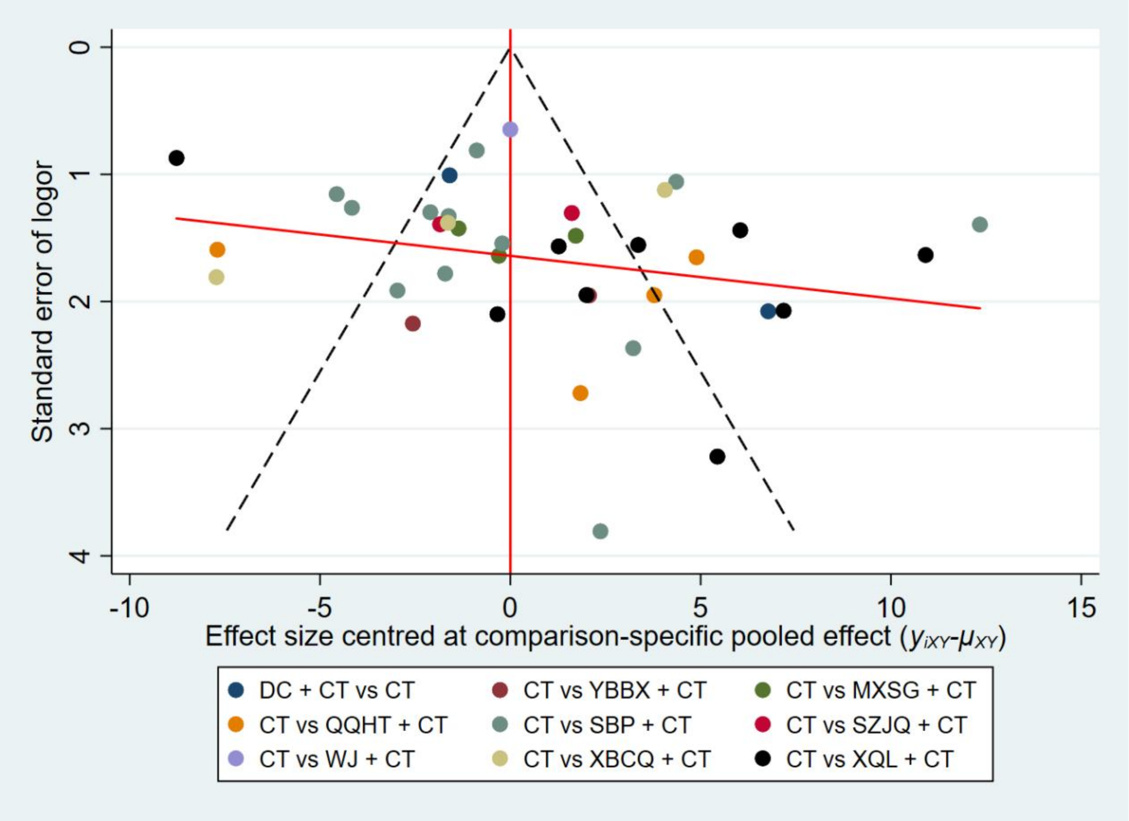

Appendix Fig A.13 $PaCO_2$

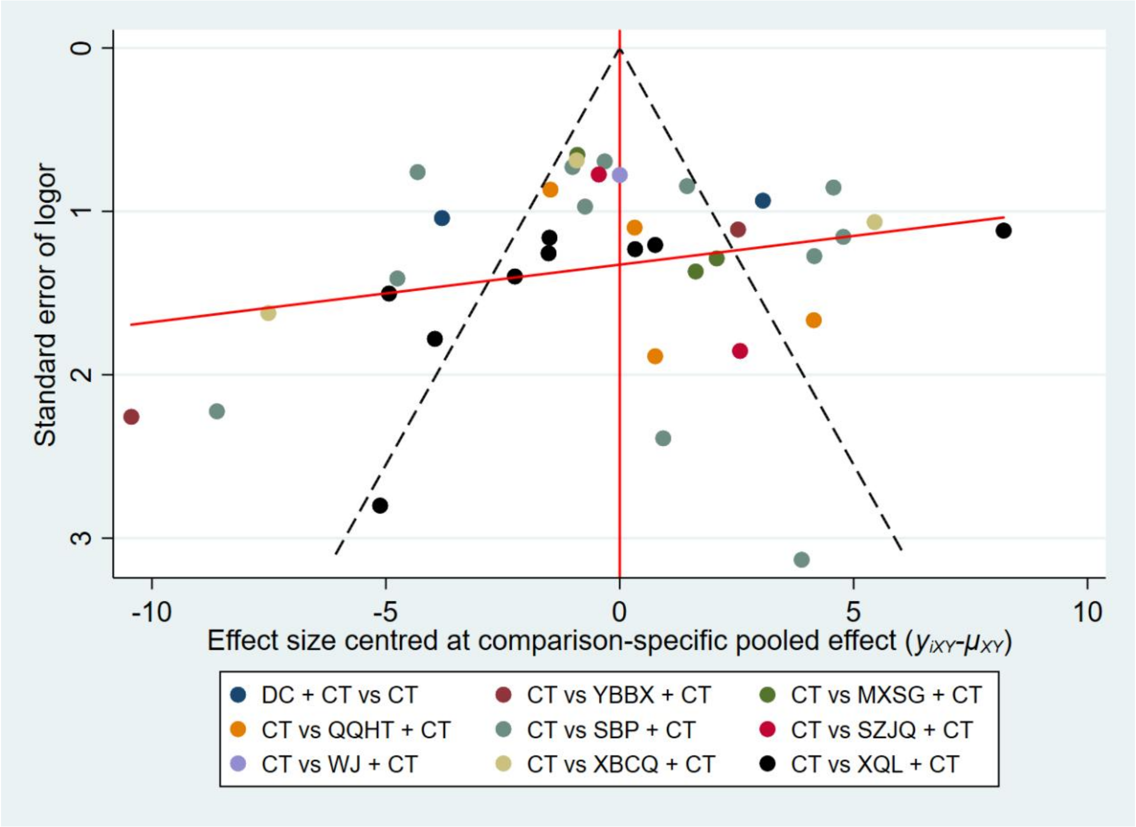

Appendix Fig A.14 Effective rate

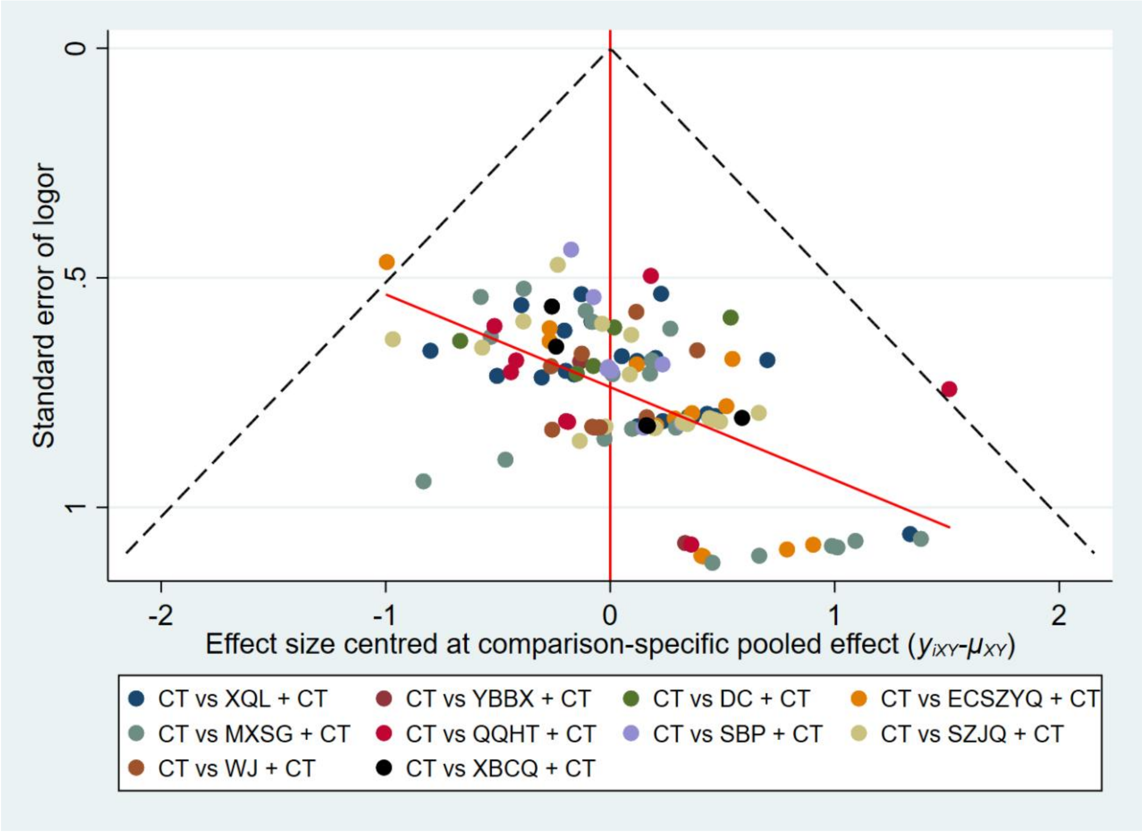

Supplement: Supplementary file 1 [file DataSheet1.pdf]
